# Model interpretability enhances domain generalization in the case of textual complexity modeling

**DOI:** 10.1016/j.patter.2025.101177

**Published:** 2025-02-06

**Authors:** Frans van der Sluis, Egon L. van den Broek

**Affiliations:** 1 Department of Communication, University of Copenhagen, Copenhagen, Denmark; 2 Cybernetics Group, Department of Information and Computing Sciences, Utrecht University, Utrecht, the Netherlands

**Keywords:** model interpretability, domain generalization, out-of-distribution testing/evaluation, accuracy-interpretability trade-off, deep learning, generalized linear models, ChatGPT, data shifts, textual complexity

## Abstract

Balancing prediction accuracy, model interpretability, and domain generalization (also known as [a.k.a.] out-of-distribution testing/evaluation) is a central challenge in machine learning. To assess this challenge, we took 120 interpretable and 166 opaque models from 77,640 tuned configurations, complemented with ChatGPT, 3 probabilistic language models, and Vec2Read. The models first performed text classification to derive principles of textual complexity (task 1) and then generalized these to predict readers’ appraisals of processing difficulty (task 2). The results confirmed the known accuracy-interpretability trade-off on task 1. However, task 2’s domain generalization showed that interpretable models outperform complex, opaque models. Multiplicative interactions further improved interpretable models’ domain generalization incrementally. We advocate for the value of big data for training, complemented by (1) external theories to enhance interpretability and guide machine learning and (2) small, well-crafted out-of-distribution data to validate models—together ensuring domain generalization and robustness against data shifts.

## Highlights

• Interpretable models outperform deep ones in a human generalization task
• Data shifts reveal the limits of deep models on textual complexity tasks
• Linear interactions enhance generalization while maintaining transparency
• Findings challenge the interpretability-accuracy trade-off for out-of-distribution tasks

## The bigger picture

Machine learning has revolutionized text analysis, but this revolution has a price: deep learning sacrifices interpretability, making it hard, and often impossible, to understand how they work. At the same time, their ability to generalize to new tasks and domains remains a challenge, particularly when data shifts occur—changes in text genre, topic, and/or human judgment criteria. Our findings challenge the assumption that interpretability comes at the cost of performance and suggest that interpretable approaches offer unique advantages for modeling human judgments, particularly when training data are limited and/or generalization is required. We provide empirical evidence and a methodological framework to advance interpretable machine learning.

## Teaser

Deep learning dominates modern machine learning, but models often struggle to generalize when data shift, and their decision processes remain opaque. This study shows that interpretable models—enhanced with linear feature interactions—can outperform deep models. These findings highlight the potential of interpretable models to deliver both transparency and robust generalization, particularly in real-world tasks with limited training data, data shifts, and diverse evaluation contexts.

## Introduction

Data-intensive models have become the *de facto* standard for modeling and predicting a wide variety of phenomena. From speech recognition and visual object detection to drug discovery and genomics, machine learning techniques, particularly deep learning, consistently outperform conventional approaches.^1^ In text analysis, neural models excel in classification tasks and significantly reduce perplexity in language modeling. This trend also extends to tasks like sentiment analysis, spam detection, authorship attribution, and—in the present case—textual complexity modeling.^2^ The successes of (deep) machine learning techniques can be attributed to their ability to address phenomena that were previously thought intractable due to their sheer size or complexity, often exceeding the human capacity for comprehension.^3^^,^^4^ This ability is enabled by the availability of near-exhaustive training data, representing all, or a substantial portion, of the conditions relevant to the examined phenomenon.^4^ Crucially, deep neural networks attain these abilities without handcrafted feature representations; they can deduce a relevant representation directly from raw inputs such as pixels, characters, or words.^1^^,^^5^ Their success signifies a broader trend in data-intensive modeling favoring larger datasets and raw inputs or, in other words, bigger data and smaller datums.^6^

Despite their impressive performance, data-intensive models face growing concerns related to interpretability,^7^ explainability,^8^ and generalizability,^9^^,^^10^ especially as machine learning applications extend into real-world, high-stakes environments.^11^^,^^12^ The rise of deep learning has often meant sacrificing model transparency for accuracy, leading to limited insight into model behavior and decision-making processes—a drawback that has fueled the field of explainable artificial intelligence (xAI).^8^^,^^13^ Simultaneously, reliance on vast datasets assumed to be independent and identically distributed (IID) (also known as [a.k.a.] ID, i.i.d., and iid) has overlooked real-world shifts in data distributions, leading to unexpected drops in model performance when tested out of distribution (OoD).^11^^,^^12^ This challenge, also known as domain generalization,^9^^,^^10^ emphasizes the importance of validating model robustness across diverse data domains, shifting the traditional train-validate-test paradigm to prioritize generalization under data shifts. Our study addresses these two critical aspects—interpretability and generalizability—through a comparative, empirical investigation in textual complexity modeling,^14^ a domain where alignment with human understanding is essential and models face diverse data environments.

### Interpretability

Contrary to the trend toward large, deep models is a recent shift toward interpretable models.^15^^,^^16^^,^^17^^,^^18^^,^^19^ Central to this movement is the use of a few, inherently interpretable features combined with a simple, human-understandable model. This combination provides transparency by explaining which feature combinations produce an outcome and control by enabling users to specify which features are deemed (ir)relevant to the modeled phenomenon. The resultant interpretable models are believed to not only grant users the ability to make informed decisions regarding their trustworthiness^13^^,^^16^^,^^20^ but also make it easier to identify and correct errors or biases,^21^ leading to more robust and reliable systems.^22^ For example, through selective feature design, interpretable models can avoid or mitigate biases in training data, such as textual genres or cultural stereotypes.^23^ Theoretical grounding enhances interpretability by ensuring that input features are relevant and that model structures are logically consistent with the phenomenon being modeled. This shift aligns with Occam’s razor, favoring models that are no more complex than necessary to represent a phenomenon effectively.^24^

A key factor enabling the use of simpler models is the Rashomon effect, which suggests that complex data, including human cognitive processes, can often be accurately modeled by many different models that achieve similar performance.^25^ This concept, introduced by Breiman,^24^ highlights that there can be a multitude of valid yet distinct models that fit the data well. However, even though the Rashomon effect suggests that simple, interpretable models can often perform as well as more complex ones, interpretable models frequently fall short in effectiveness. Data-intensive models, particularly those using deep learning, typically achieve state-of-the-art performance,^1^ leading to an ongoing debate about the interpretability-accuracy trade-off.^15^ This debate centers on not only the trade-off itself but also the difficulty of finding accurate, simple models. As Semenova et al.^26^ observes, “It is almost always easier to find an accurate-but-complex model than an accurate-yet-simple model. Finding optimal, sparse, accurate models of various forms … is generally NP-hard. We often do not know whether the search for a simpler model will be worthwhile, and thus we do not go to the trouble of searching for one.” This observation raises the question of which model we should choose and when it is worth pursuing simplicity.

Some studies challenge the interpretability-accuracy trade-off, showing that interpretable models can be competitive in certain conditions. In domains that lend themselves to feature engineering and tasks involving tabular data, comparisons between different classification heads on the same task suggest that interpretable models can achieve a comparable level of performance. For instance, Razavian et al.^27^ showcased the comparable performance of a linear regression model to tuned random forests, gradient-boosted decision trees, and neural networks in a disease prediction task, Tollenaar and van der Heijden^28^ and Zeng et al.^29^ show that linear regression models produce equally accurate predictions of recidivism, and Kung and Yu^30^ likewise show on-par performances of regression models for college success predictions. Conversely, few comparisons exist for non-tabular, textual data. Notable exceptions are Yadav et al.,^31^ who demonstrated the competitive performance of interpretable Tsetlin machines (TMs) against various neural networks in a sentiment analysis task, and Singh et al.,^32^ who showed the competitive performance of interpretable, linear models against large language models (LLMs) on text classifications tasks. A plausible explanation is that textual domains are less suitable for representation through simple models with a limited number of features. As a result, while such head-to-head comparisons are increasingly reported, especially in domains involving tabular data, they remain relatively uncommon in textual, non-tabular domains.

### Generalizability

Performance benchmarks on identical datasets may, furthermore, not provide a comprehensive understanding of model efficacy. The reason is that typical validation methods, such as cross-validation and train-test splits, often fall short when it comes to evaluating a model’s ability to generalize beyond its training data. Since these methods draw from the same distribution as the training set, they offer a limited view of the model’s performance in new, unseen contexts. Without diverse and representative training data, models risk overfitting to noise (i.e., variance error) or lack the necessary information to learn the target function accurately (i.e., estimation error). In some cases, in-sample variations can even lead to shortcut learning, where models predict the right answer for the wrong reason^16^ by relying on dataset artifacts rather than learning meaningful patterns.^33^^,^^34^ To develop models that generalize robustly across different conditions, OoD validation, also known as domain generalization, is essential.^10^ Domain differences between factors such as sampling location,^33^ time, or—particularly in natural language processing (NLP)—variations in genre, writing style, and topic may lead to data shifts in feature distributions (i.e., covariate shifts). Domain generalization studies evaluate models’ ability to handle these shifts, revealing the strengths and limitations of different modeling approaches in OoD situations.

In addition to OoD evaluations across different domains, human generalization evaluates models by directly comparing their outputs against human judgments. This practice, common in fields where model training and evaluation involve different tasks, often introduces both label shifts and covariate shifts, resulting in a full shift in data distributions. Label shifts arise because the measurement scales used for human validation differ from the labels used in training. Covariate shifts occur because the data used for human validation may differ in domain, context, or other properties from the training data. For instance, human generalization studies can capture nuances through graded, multi-faceted rating scales (e.g., sentiment strength^35^), evaluate model adaptability to complex inputs (e.g., adversarial examples^36^), contextualize evaluations for specific use scenarios (e.g., decision-making contexts^37^), and align models with the expertise and needs of diverse user groups (e.g., physicians^38^). Together, these practices broaden model validity across diverse inputs, nuances, contexts, and populations.^39^ Due to the difficulty of obtaining valid and varied data through human studies, human judgments are typically too limited in size to serve directly as training data. Instead, they reveal areas where generic models must generalize effectively from training data without re-learning, demonstrating the model’s ability to handle full shifts in data distributions (cf. van der Sluis et al.^14^).

Generalization studies favor model configurations that not only work well on training data but also extend to cross-domain or human validations. Possible workarounds are through transfer learning and fine-tuning approaches, wherein models re-learn specifics for each new dataset or task given the availability of sufficient data for re-learning. Alternatively, to create generic models that generalize without re-learning, model designs need to navigate two classical modeling trade-offs: the approximation-estimation and bias-variance trade-offs. The approximation-estimation trade-off balances a model’s capacity to represent complex relationships (approximation) against the need for sufficient data to accurately estimate these relationships (estimation). The bias-variance trade-off addresses the inherent assumptions within a model’s design (bias) vs. the model’s sensitivity to variations within the training data (variance). Both the approximation error and bias error are caused by the choice of model. For instance, various techniques have been explored to reduce the sensitivity of (deep) machine learning models, including regularization, ensemble models, and adversarial learning.^40^^,^^41^ Interpretability serves as one of such techniques, where the refined control over the (ir)relevance of features and the ability to inspect the relationships modeled provide a means to manage noise and inherent biases in training data.^21^ This benefit of interpretability has been observed in fields such as physics-based and physician-built models,^42^^,^^43^^,^^44^ where simpler, transparent models are applied reliably across diverse contexts, though it has not been consistently observed in other fields such as cancer transcriptomics.^45^ Even though this provides additional motivation to pursue a generic, simple-yet-accurate model in generalization settings, the extent to which interpretability enhances generalizability for textual tasks and with human validation remains uncertain.

### Textual complexity

Automatic readability detection, also known as textual complexity modeling, aims to assess and predict the difficulty of texts. Common tasks in this domain include classifying texts according to their complexity level or predicting expert and reader ratings of difficulty.^14^ Textual complexity modeling plays an essential role in fields such as education,^46^ content personalization,^47^ and information retrieval,^48^ where aligning text readability with audience capabilities and needs is important. Textual complexity can be considered a complex phenomenon, mirroring the intricacies of human language comprehension.^49^ This complexity makes it unlikely that a purely theoretical causal structure can be derived, instead favoring data-intensive approaches. However, interpretability remains valuable, particularly in “educational contexts, where teachers or students may need to understand the factors that make a text difficult” (Collins-Thompson,^50^ p. 105). As a complex phenomenon, textual complexity offers an ideal case to compare interpretable and advanced models.^14^ A plethora of psycholinguistic studies (e.g., Balota et al.,^51^ New et al.,^52^ and McGinnies et al.^53^) has explored how specific text features, such as word familiarity and semantic priming, impact human processing difficulty, providing a basis for interpretability. Historically (for an overview, see supplemental note A), textual complexity has been modeled using interpretable methods, like linear regression with linguistically or cognitively motivated features.^54^^,^^55^ However, this inherent complexity also favors advanced models capable of capturing nuanced relationships among features. Recent advancements in AI have underscored the success of deep models in classifying texts by complexity,^56^^,^^57^^,^^58^ establishing a basis for comparative analyses.

The availability of ample training data for textual complexity modeling has been considered a challenge.^59^ The intricacies of the phenomenon make it unlikely that exhaustive data will be available from which to induce a causal structure. In addition, these intricacies make it likely that any model needs to uncover higher-order interactions between input features, posing further requirements for the training data to cover all relevant variations within an inflated feature space. This reflects the approximation-estimation trade-off, where a model’s limited representational capacity (approximation) and the finite, likely insufficient, training data (estimation) constrain the model’s accuracy in capturing complex relationships fully. Current readability studies address these sampling requirements by employing data that are representative of populations, genres, document properties (e.g., language and length), and reading level.^50^ Notable data sources include the Common Core Appendix B (168 documents, grade levels 2–12), WeeBit (6,388 documents, 6 grade levels), and Wikipedia (up to 2,62,918 documents, binary levels).^60^ However, the use of a representative dataset results in models specialized according to a specific target.^59^ Generalization studies confirm this specialization, revealing drops in performance for new yet similar datasets.^54^^,^^55^^,^^56^^,^^59^ They underscore the challenge of capturing generic principles of textual complexity from training data, favoring generalization studies that test models across diverse domains of text.

This challenge is further compounded by a subjective component in textual complexity. Subjective differences become apparent from a characteristically low inter-rater agreement, even though typical readability datasets rely on expert assessments. These differences are simultaneously reflected in a reduction in model performance between similar and dissimilar experts^61^ and between more objective and subjective measurements, even after re-training.^62^ Such domain and human generalization studies highlight the variation that exists between and within individuals. Individual traits such as prior knowledge and reading proficiency combine with temporal cognitive and motivational processes to determine a reader’s comprehension of a text^63^ and, equally so, their experienced processing difficulty. This variation necessitates models to differentiate a generic component from an inherently individual, subjective component of textual complexity.^50^ Ideally, a generalizable model of complexity goes beyond expert assessments to subjective judgments of processing difficulty, underscoring the human-centered challenge of aligning with diverse human experiences and understandings.

### Research outline

**Figure 1:**
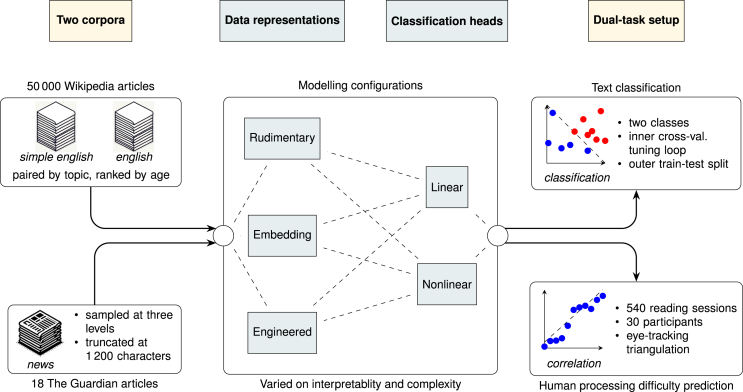
Schematic overview of study methodology Lines illustrate flow of data, with dashed lines indicating evaluated modeling configurations. Paper stacks were generated using Dall-E on January 29, 2024.

Par excellence, textual complexity poses a data-intensive modeling challenge. Its foundation in the intricate processes of human comprehension makes it a complex phenomenon, highlighting the challenge of obtaining near-exhaustive training data. Furthermore, its partial subjectivity highlights the need for generalization studies that evaluate models on a subjective component. This imperative favors a generalization setup, recognized as a hallmark in modeling benchmarks.^64^ To this end, this study introduces a dual-task setup for the comparison of various modeling approaches, detailed in Figure 1 and Table 1. The primary task is a text classification task in which models are trained and tested on their ability to classify texts according to two levels of complexity. The secondary task is a correlation task in which models are evaluated on their ability to predict human judgments of processing difficulty. The aim is for models to derive generic principles of textual complexity from training data that generalize to human judgments. This dual-task setup facilitates a comprehensive comparison, allowing us to assess modeling approaches based on their capacity to induce a generic and human-generalizable model.

**Table 1:** Modeling configurations evaluated within the dual-task setup

	Classification models	
**Feature sets**	**Linear**^a^	**Nonlinear**

Rudimentary^b^	PLM	Vec2Read and ChatGPT^c^
	3 variations	2 models
Embedding	BERT and GLM	BERT and FNN
	61 variations	83 variations
Engineered	PDfeat and GLM	PDfeat and FNN
	59 variations	83 variations

Configurations include the feedforward neural network (FNN), probabilistic language model (PLM), and generalized linear model (GLM), with engineered features (PDfeat) and bidirectional encoder representations from transformers (BERT) embeddings. Within each configuration, diverse model variations are trained, spanning different model depths and parameter counts. In total, variations alongside baseline models account for 291 distinct models of textual complexity.

a Linear models are able to capture some degree of nonlinear relationships from training data; see supplemental note B.

b Baseline models with integral data representation.

c ChatGPT is only applied to the generalization task.

For our training data, a dataset^65^ consisting of 50,000 easy and difficult Wikipedia articles forms the foundation from which to infer generic principles of textual complexity. Articles were matched into pairs to mitigate the potential influence of the topic as a confounding variable. Furthermore, articles were selected based on their age to ensure a mature and likely high-quality sample. This strategic selection and pairing of articles is expected to help reveal relevant conditions and reduce the presence of irrelevant conditions in the training data. For our target data, we used a dataset^66^ from a study involving 30 participants who appraised the processing difficulty of 18 (truncated) news articles across 540 reading sessions. Articles were sampled at the easiest, medium, and highest levels of complexity from a set of 14,856 articles using a preliminary complexity model. Subjective appraisals were obtained in a controlled laboratory environment and triangulated with eye tracking to ensure high-quality data, as reported in van der Sluis and van den Broek.^67^ The relatively small size of the target data, where 30 readers appraise a sample of articles, contrasts with the large training dataset typically used, where few experts label many articles. The controlled environment and the number of participants make it likely that nuances in participants’ reading experiences were captured. This setup introduces a label shift from binary complexity labels in Wikipedia training data to graded, reader-based appraisals of processing difficulty in the target data. Additionally, a covariate shift can be expected from domain differences in genre, topic, and article lengths between Wikipedia’s encyclopedic content and the journalistic style of The Guardian articles.

Table 1 details the modeling configurations assessed in the dual-task setup. The experiment compares three types of data representations: (1) rudimentary representation, which remains close to the raw input (e.g., sequences of characters, words, and part-of-speech [POS] tags^68^), (2) embedding representation, derived from a learned feature representation using a (deep) language model (i.e., bidirectional encoder representations from transformers [BERT]), and (3) feature representation, founded on a formalization of reviewed psycholinguistic findings. All three representations capture some degree of syntactical, structural, and semantic aspects of text useful for modeling textual complexity. Rudimentary and embedding spaces implicitly capture these dimensions, with BERT reflecting a rich array of linguistic features.^69^ The engineered feature set explicitly computes these aspects, grounded in well-established psycholinguistic findings and theoretical paradigms, which adds to the validity and establishes the interpretability of the features. These representations correspond to various configurations of the bias-variance trade-off, defining aspects of an external theory that frames the modeling problem.^4^ The more explicit the representation, the more constrained the subsequent learning process, defining what features a model can learn and restraining the interaction space.

The experiment contrasts two model categories, detailed in Table 1: (1) linear models designed to capture linear relationships and n-degree multiplicative interactions within the data representation and (2) nonlinear models designed to capture nonlinear relationships within the data representation. Two aspects of the models are systematically varied for the embedding and feature representations: interaction depth (three levels) and model parameters (26–153,012 parameters). Interaction depth enables models to uncover higher-order interactions between input features, better equipping them to capture the understood intricate nature of textual complexity. The number of model parameters specifies the configurations of values a model should learn from the training data, which need to be appropriate given the amount of observations available for learning. Together, these aspects form different configurations of the approximation-estimation trade-off. A deep model can capture complex relationships, while a well-regulated model increases the likelihood of distinguishing relevant configurations from spurious ones. Of these configurations, only linear models with engineered features can be considered inherently interpretable, whereas other configurations might be explainable.^15^

The primary objective of this study is to systematically compare various modeling options to establish a generic and generalizable model of textual complexity. Through the systematic variation of feature sets and modeling approaches, we seek to assess the impact of interpretability and model complexity on the generalizability of resultant models. This comparative investigation adds to a limited yet expanding body of evidence on the effectiveness of interpretable models vs. opaque ones.^15^ Additionally, the combination of a large dataset for training with a small, controlled experimental dataset for generalization creates an OoD evaluation. This setup challenges models to apply learned principles to new, unseen scenarios that differ along multiple dimensions. Genre (encyclopedic vs. news articles), tasks (text classification vs. processing difficulty), measurements (objective vs. subjective), and assessors (few experts vs. 30 readers) all differ between dataset I and dataset II. While finding an optimal, interpretable model is considered NP hard,^26^ these differences introduce an additional, challenging full data shift for generalization. By studying interpretability and human generalization in tandem, our work contributes both empirical evidence and a methodological framework to the study of the interpretability-accuracy trade-off.

## Background and related work

Generalization, the ability of a model to perform well on new, unseen data, is a core objective in NLP. However, achieving robust OoD generalization remains challenging due to the diverse and dynamic nature of language data. NLP models often encounter a variety of data shifts—systematic changes in data distributions—that impact their ability to generalize effectively. Let x represent the input features and y denote the output or labels, and then Hupkes et al.^64^ define covariate shifts when the input distribution (p(x)) changes between training and validation data (i.e., $p\left(x_{\text{tst}}\right)\neq p\left(x_{\text{tr}}\right)$), but the conditional relationship p(y|x) remains the same, allowing the evaluation of a model’s ability to learn the underlying phenomenon. Label shift, on the other hand, happens when p(y|x) changes due to variations in output distribution, such as inter-annotator disagreements or changes in tasks, affecting the model’s ability to handle varying interpretations or labeling across contexts.^70^ Full shift is the most extreme type of shift, where both the input and output distributions change simultaneously (i.e., $p\left(x_{\text{tst}}\right)\neq p\left(x_{\text{tr}}\right)$ and $p\left(y_{\text{tst}}|x_{\text{tst}}\right)\neq p\left(y_{\text{tr}}|x_{\text{tr}}\right)$).

Generalization research in NLP has developed several paradigms to evaluate model performance across data shifts: cross-task, cross-domain, and robustness generalization.^64^ Cross-task generalization assesses a model’s ability to apply its knowledge across multiple NLP tasks, typically through pretraining on a broad objective followed by fine-tuning on task-specific parameters. This method tests how well general linguistic knowledge can adapt to diverse tasks, with or without task-specific model adjustments.^71^ Cross-domain generalization measures a model’s adaptability to different types of text. Domains in NLP can vary by genre, formality, or topical focus, such as adapting a model trained on news articles to scientific literature.^72^ It evaluates a model’s robustness to diverse, naturally occurring language contexts. Robustness generalization focuses on a model’s ability to avoid relying on spurious correlations within training data. This type of generalization targets unintended data shifts, such as annotation artifacts or demographic imbalances, ensuring that the model’s predictions align with the modeled phenomenon rather than superficial data patterns.^73^ Of these, the first generalization paradigm mostly targets label shifts, whereas the others mostly cover covariance shifts.

In textual complexity research, several studies have investigated the generalizability of models across different corpora of text difficulty. Cross-domain experiments, which test models on unseen datasets, reveal performance declines due to covariate shifts. For instance, models trained on the WeeBit corpus and tested on Common Core standards showed correlation drops from *r* = 0.74 to 0.49,^55^ from 0.92 to 0.69,^54^ and from 0.900 to 0.730.^56^ In the latter case, performance on the target corpus improved to *r* = 0.905 by expanding and mapping the feature space, though this adjustment requires access to target data for re-training.^56^ Performance is generally more stable for models that include engineered features. For example, the regression model CAREC improved slightly from *r* = 0.571 on crowd-sourced evaluations to *r* = 0.580 on the CommonLit ease of readability (CLEAR) corpus.^74^^,^^75^ Similarly, a hybrid model incorporating engineered features in a neural network showed only minor shifts, with performance varying from 0.685–0.699 to 0.639–0.683.^76^ Interestingly, cross-language experiments within a similar domain also show relatively stable performance. For instance, models trained in English and tested on related corpora in different languages (English-French and English-Spanish) reported performance declines from *r* = 0.99 to 0.90–0.95.^57^ These findings illustrate the varied effects of covariate shifts on readability models. Cross-domain shifts—such as changes in genre or topic—impact performance most significantly. While feature engineering or adaptation techniques, such as feature mapping, can help maintain model accuracy, these methods depend on the features used or the availability of target labels, complicating generalization to new, OoD datasets.

In addition to testing across different corpora, a few studies have examined the generalizability of models across varying measures of text difficulty. Cross-measurement performance typically involves comparing expert judgments and measurement scales. For instance, model performance drops from 73% to 59% when generalizing from experts with aligned judgments to those with more diverse opinions.^61^ Likewise, when training on subjective ratings of text processing difficulty and testing on comprehension difficulty, correlations decrease from *r* = 0.683 to 0.557.^62^ These substantial reductions in cross-measurement performance highlight the subjectivity involved in assessing reading difficulty (see introduction, textual complexity). This subjectivity leads to differing interpretations of text complexity, creating label shifts.

Our study extends the existing landscape of generalization research with a full shift study involving both label and covariate shifts. The covariate shift results from genre and topical differences: Wikipedia’s encyclopedic content contrasts with the journalistic style of The Guardian articles, creating variability in topics, writing style, and text length. The label shift arises from moving from binary complexity labels in training to a graded measure of processing difficulty. While Wikipedia reflects diverse contributor expertise, the processing difficulty measure captures varied cognitive demands experienced by readers, shaped by their backgrounds and interests. Through a controlled experiment, this measure was triangulated with eye-tracking data (see Figure 1), offering a rigorous, OoD human experiment that precisely captures readers’ experiences beyond simple labeling. Together, these shifts provide a robust foundation for evaluating the generalizability of different models of textual complexity.

## Results

A total of 65,640 feedforward neural network (FNN) and 12,000 generalized linear model (GLM) configurations were tuned, resulting in 166 FNN and 120 GLM models. The resulting configurations were subsequently trained and evaluated through cross-validation on dataset I (i.e., training or classification task) and evaluated using Pearson’s correlation with processing difficulty for dataset II (i.e., target or generalization task). After tuning, a total of 61 GLM and 83 FNN models were trained on BERT embedding features and 59 GLM and 83 FNN models on PDfeat-engineered features, complemented with 5 baseline models including ChatGPT, 3 probabilistic language models (PLMs), and Vec2Read.

### Models’ performance

**Figure 2:**
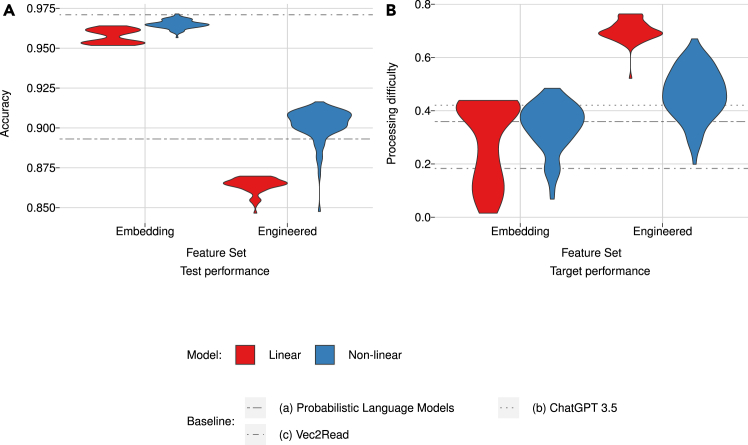
Distribution plots of models’ performance per combination of feature set and model type (A) Classification accuracy (Acc.) on the EnSiWiki2020 corpus. (B) Models’ correlation coefficient with processing difficulty (Proc. Diff.) as measured for The Guardian texts. Baseline results are included as horizontal lines in both graphs when available. The best-performing baseline model is shown for probabilistic language models, ensemble performance for ChatGPT 3.5, and performance after 100 training epochs for Vec2Read.

Figure 2 visually represents the performance distribution of models on both datasets, revealing two notable contrasts with a distinctive mirror-like pattern. First, the figure shows that FNN models outperform GLM models in classification, whereas the reverse pattern appears for predicting processing difficulty. With embedding features, GLM and FNN models show little difference in generalization performance, but with engineered features, GLM models markedly outperform FNN models. Second, it illustrates that embedding features outperform engineered features in the classification task, while the reverse pattern emerges in predicting processing difficulty. In particular, the latter differences are by a large margin. This mirror-like pattern highlights a stark difference in how model and feature types perform across classification and generalization tasks.

Figure 2, furthermore, shows the performance of three baselines. On the text classification task, Vec2Read outperformed all other models, whereas the 3 PLMs shared a level of performance similar to engineered features. ChatGPT was not scalable to this task. On the generalization task, ChatGPT outperformed both the PLMs and Vec2Read. The level of ChatGPT was on par with the best-performing embedding-based models, even though ChatGPT was not specifically trained or tuned toward this task. These baseline models set an exceedingly high target for the text classification task but also confirm the difficulty of the generalization task.

### Model depth and parameters

**Figure 3:**
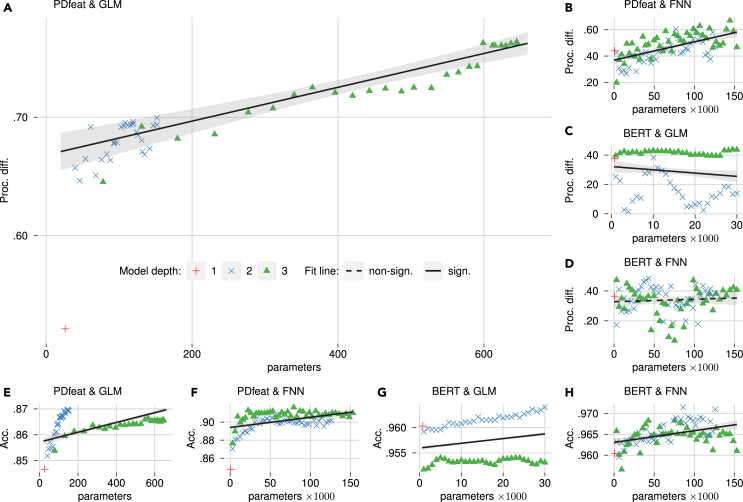
Models’ performance summarized by the number of model parameters and model depth (A–D) Models’ correlation on the processing difficulty prediction task. (E–H) Models’ accuracy on the text classification task. The number of parameters is derived from the number of input features and model complexity (see methods). Model depth is either the degree of multiplicative interactions between features for GLMs or the number of layers for FNNs. The fit lines, along with their 95% confidence intervals and statistical significance, illustrate the estimated marginal effect of parameters on performance within regression models (see Table 2).

A comprehensive analysis of the influence of model depth and parameters on both tasks is presented in Figure 3, complemented by a statistical examination provided in Table 2.

**Table 2:** Regression models that examine the influence of the number of parameters, the model depth, and their interaction on the performance of textual complexity models

	Parameters		Depth		Interaction		Fit	
**Model**	β	*t*	β	*t*	β	*t*	*F*	$R^{2}$

**Test performance**								

SB23 and FNN	1.71	5.04^∗∗∗^	0.97	7.84^∗∗∗^	−1.48	−4.03^∗∗∗^	40.71^∗∗∗^	0.61
BERT and FNN	2.31	5.52^∗∗∗^	0.32	2.08^∗^	−2.05	−4.50^∗∗∗^	17.34^∗∗∗^	0.40
BERT and GLM	1.17	11.95^∗∗∗^	−0.63	−16.01^∗∗∗^	−1.06	−10.04^∗∗∗^	655.60^∗∗∗^	0.97
SB23 and GLM	18.99	22.75^∗∗∗^	1.03	10.73^∗∗∗^	−19.65	−22.17^∗∗∗^	184.61^∗∗∗^	0.91

**Target performance**								

SB23 and FNN	0.61	7.51^∗∗∗^	0.25	3.03^∗∗^	–	–	37.46^∗∗∗^	0.48
BERT and FNN	0.08	0.69	−0.14	−1.23	–	–	0.88	0.02
BERT and GLM	−1.24	−4.06^∗∗∗^	0.44	3.59^∗∗∗^	1.20	3.64^∗∗∗^	49.86^∗∗∗^	0.72
SB23 and GLM	8.42	7.98^∗∗∗^	0.78	6.45^∗∗∗^	−8.24	−7.36^∗∗∗^	108.78^∗∗∗^	0.86

Possible colinearity of the interaction term was addressed via backward stepwise selection, with missing values indicating its removal. The degrees of freedom for the *t* and *F* tests are t(57) and F(3,57) for the GLM regression models and t(79) and F(3,79) for the FNN regression models. Significance levels: ^∗∗∗^p < .001, ^∗^ p < .01, ^∗^p < .05.

Overall, the figure reveals a positive correlation between the number of parameters and performance in the text classification task, with the most significant impact observed for the PDfeat feature set. For the generalization task, parameter count contributes substantially to generalization performance for the PDfeat-GLM and PDfeat-FNN combinations. In contrast, BERT-based models exhibit either no correlation or a negative relationship between parameter count and generalization performance. In fact, parameter count has a small, negative, but significant effect on generalization performance for BERT-GLM, as shown in Table 2. These findings suggest that while more complex models enhance classification performance, they may impede generalization to the generalization task, particularly for BERT-based configurations.

Model depth generally shows a positive influence on training and generalization performance for most configurations in Table 2, indicating that greater depth enables models to better capture and generalize textual complexity. However, exceptions emerged with BERT features: GLM-depth negatively impacted test performance, while FNN-depth failed to enhance generalization performance. For GLM configurations (PDfeat-GLM and BERT-GLM), peak test performance consistently occurred at depth level 2 (Figures 3E and 3G), while peak generalization performance shifted to level 3 (Figures 3A and 3C). This suggests that 3-level multiplicative interactions in GLMs may obscure simpler patterns needed for classification, ultimately benefiting generalization. Conversely, for BERT-FNN configurations, peak performance for both tasks was observed at depth level 2. Additional depth likely focused on corpus-specific patterns, explaining why FNN-depth failed to enhance generalization performance with BERT features. These findings highlight that depth configurations optimizing test performance may not always translate to improved generalization and can sometimes hinder it. The overall effects of depth are modulated by interactions with other factors, particularly parameter count as a covariate to depth level and interaction type (i.e., linearity).

### Feature contributions

**Figure 4:**
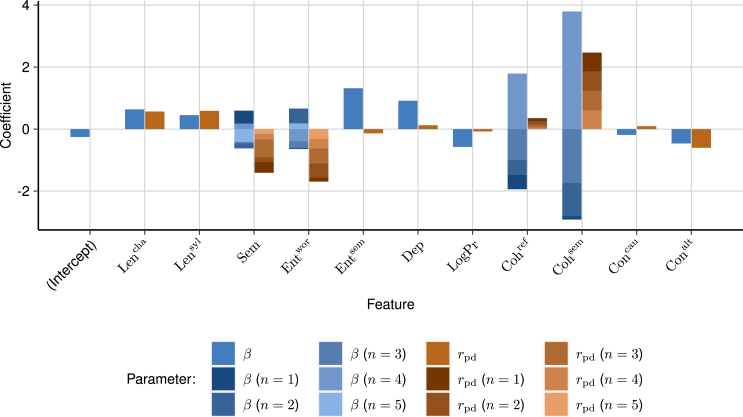
Feature importance as indicated by stacked coefficients for both training and target data Parameterized features, such as cohesion over n=1…4 foregoing sentences, are grouped and stacked. Coefficients are either standardized regression coefficients (β) derived from the final GLM model with feature depth level 1 or Pearson’ correlation with processing difficulty $\left(r_{\text{pd}}\right)$. Note that parameterized feature regression coefficients are likely inflated inflated due to complementary yet opposing influences in the model. Negative values are indicative of simpler texts and positive values of more complex texts.

Figure 4 provides an overview of the PDfeat features and their test and target correlations. Most features align with expectations: word length $\left({\text{Len}}^{\text{cha}},{\text{Len}}^{\text{syl}}\right)$, semantic entropy $\left({\text{Sem}}_{n}\right)$, and dependency length (Dep) correlate positively with complexity, while connectives $\left({\text{Con}}^{\text{cau}},{\text{Con}}^{\text{alt}}\right)$ and word frequency (LogPr) tend to simplify texts. Furthermore, parameterized features exhibit both positive and negative relations with complexity due to their additive effects. For instance, semantic and referential cohesion over 3–4 preceding sentences positively contribute to complexity, while cohesion over 1–2 preceding sentences has a negative impact. This implies that simpler texts employ fewer references over longer distances and more references over shorter distances. These findings indicate that features conform to expectations and are accurately derived from the training data.

Comparing the behavior of features between training and target data in Figure 4 reveals some discrepancies. On the target data, referential and semantic cohesion are associated with more complex texts, while lexical entropy is linked to simpler texts, opposing the test data and initial expectations. These counterintuitive findings emphasize the unique characteristics of the target data drawn from a distinctive corpus of well-edited and refined news articles. This small, carefully selected set of articles presents its own specifics, which may not align with other corpora. While certain patterns may appear when features are considered individually in such a sample, a more accurate interpretation likely emerges when these features are viewed in conjunction with others. This underscores the complexity of accurately modeling textual nuances across diverse datasets, where the specifics of the target corpus amplify the generalization requirements posed by the generalization task.

Table 3 compares a selection of feature interactions along with their test and generalization performance. Compared to first-order features (Figure 4), second-order and third-order interactions exhibit a higher correspondence between test and target metrics. Conversely, model coefficients β for these interactions differ from both test and target metrics. This discrepancy reflects the additive effects resulting from numerous interactions in the model, complicating the direct interpretation of model coefficients for higher-order interactions. The increased alignment between test and generalization performance with multiplicative interactions offers an explanation for the heightened generalization performance observed with higher model depths (see Figure 2). Most top-10 interactions span across different features rather than feature parameters, suggesting that textual complexity arises from a simultaneous combination of factors across various semantic, lexical, and syntactic aspects.

**Table 3:** Top 10 interactions between engineered features, ranked by regression coefficient β

Features	Test $r_{pb}$	Target r	Model β
**Second-order interactions**			

${\text{Sem}}_{1}$, ${\text{Sem}}_{2}$	−0.07	−0.29	0.47
${\text{Len}}^{\text{cha}}$, ${\text{Len}}^{\text{syl}}$	0.43	0.58	0.46
${\text{Sem}}_{4}$, ${\text{Sem}}_{5}$	−0.16	−0.18	0.39
${\text{Coh}}_{4}^{\text{sem}}$, ${\text{Len}}^{\text{syl}}$	0.22	0.63	−0.33
${\text{Coh}}_{3}^{\text{ref}}$, ${\text{Coh}}_{4}^{\text{ref}}$	−0.10	0.04	0.30
${\text{Coh}}_{1}^{\text{sem}}$, ${\text{Len}}^{\text{cha}}$	0.09	0.63	0.29
${\text{Len}}^{\text{cha}}$, ${\text{Sem}}_{4}$	0.27	0.35	−0.25
${\text{Coh}}_{3}^{\text{sem}}$, ${\text{Coh}}_{4}^{\text{sem}}$	0.05	0.60	0.25
${\text{Coh}}_{4}^{\text{ref}}$, ${\text{Ent}}_{1}^{\text{wor}}$	−0.05	0.05	−0.23
${\text{Coh}}_{4}^{\text{ref}}$, ${\text{Sem}}_{5}$	−0.07	0.04	−0.23

**Third-order interactions**			

${\text{Coh}}_{4}^{\text{ref}}$, ${\text{Ent}}_{1}^{\text{wor}}$, ${\text{Sem}}_{1}$	−0.04	−0.03	−0.06
${\text{Ent}}^{\text{sem}}$, ${\text{Len}}^{\text{cha}}$, ${\text{Len}}^{\text{syl}}$	0.45	0.56	0.06
LogPr, ${\text{Coh}}_{4}^{\text{ref}}$, ${\text{Sem}}_{2}$	0.01	0.03	0.04
LogPr, ${\text{Coh}}_{1}^{\text{sem}}$, ${\text{Sem}}_{2}$	−0.06	−0.70	−0.04
${\text{Coh}}_{1}^{\text{ref}}$, ${\text{Coh}}_{2}^{\text{ref}}$, ${\text{Ent}}^{\text{sem}}$	−0.16	0.12	0.04
${\text{Ent}}^{\text{sem}}$, ${\text{Sem}}_{4}$, ${\text{Sem}}_{5}$	−0.12	−0.19	0.04
${\text{Sem}}_{1}$, ${\text{Sem}}_{4}$, ${\text{Sem}}_{5}$	−0.13	−0.29	0.04
${\text{Ent}}^{\text{sem}}$, ${\text{Sem}}_{3}$, ${\text{Sem}}_{4}$	−0.14	−0.41	0.03
${\text{Con}}^{\text{alt}}$, ${\text{Ent}}_{1}^{\text{wor}}$, ${\text{Sem}}_{2}$	−0.11	−0.58	0.03
${\text{Coh}}_{3}^{\text{ref}}$, ${\text{Coh}}_{4}^{\text{ref}}$, ${\text{Sem}}_{2}$	−0.11	0.04	0.03

Regression coefficients are derived from models with second-order interactions and third-order interactions with the highest number of parameters. Test performance is indicated by interactions’ point-biserial correlation $\left(r_{pb}\right)$, and target performance is represented by Pearson’s correlation (r). Regression coefficients are generally lower for higher-order interactions due to the multiplicative nature of resulting features.

## Discussion

We compared old-world and new-world statistics: 65,640 opaque FNNs and 12,000 interpretable GLM configurations were tuned, resulting in 166 FNNs and 120 GLMs, while comparing them with 5 baseline models: ChatGPT, 3 PLMs, and Vec2Read. This allowed us to evaluate the trade-off between accuracy and interpretability and its influence on the models’ OoD generalization performance. Textual complexity classification was chosen as a task because it exemplifies this trade-off, favoring LLMs over interpretable ones to mirror the intricacies of human language processing. The predictive validity of 291 textual complexity models was assessed. Interpretability was enabled through a foundation in concepts and findings on human processing difficulty. Models’ complexity was systematically varied using linear and nonlinear classification “heads,” ranging across three depth levels and between 26 and 153,012 parameters. This array of modeling approaches and distinctive tasks created a comprehensive and challenging benchmark for evaluating the accuracy-interpretability trade-off.

Contrary to prevailing assumptions that interpretable models inherently sacrifice performance,^15^ our results show the highest generalization performance with interpretable features and transparent model architectures. This surprising level of performance extends beyond previous studies with tabular data, where well-tuned interpretable models perform on par with opaque models. This difference is likely due to the increased demand for generalization: unlike tabular datasets that may cover various sampling sites and time frames, our setup required models to generalize across distinct genres and topics as well as tasks, measurements, and assessors, introducing both covariate and label shifts. Moreover, the observed limited generalizability of opaque models contrasts with recent abilities of LLMs to generalize across a variety of tasks, as also exemplified by the best-performing baseline of ChatGPT at *r* = 0.421. These deep, opaque models derive this ability from vast corpora of human-written texts, providing near-exhaustive training data. In contrast, our models, working with more limited data, demonstrate that interpretability enables generalizability in data-scarce environments, notably surpassing the best-performing opaque baseline (ChatGPT). This suggests a reevaluation of the accuracy-interpretability trade-off, especially for tasks requiring extensive generalization across full data shifts.

The addition of feature interactions significantly enhanced generalization performance for both linear and nonlinear interactions with engineered features. This reveals that textual complexity does not solely stem from individual features but rather emerges from their interplay. This interpretation is reinforced by the importance of cross-feature interactions, as evidenced in Table 3, and aligns with the recognized intricacies of human language processing and comprehension.^49^ Linear, multiplicative interactions were particularly beneficial to generalization performance, outperforming nonlinear interactions. This benefit partially extended to BERT features, where additional linear feature depth improved generalization performance, unlike nonlinear interactions. This observed benefit can be attributed to the linear assumption. Introducing multiplicative interactions among up to three features ensures that all three features must co-occur simultaneously to influence predictions. This mechanism effectively shields the model from being influenced by irrelevant fluctuations in individual features. Furthermore, additional multiplicative interaction terms yielded surprisingly consistent incremental improvements in both classification and generalization performance (Figures 3A and 3E), similar to nonlinear interactions (Figures 3B and 3F). Accordingly, multiplicative interactions provide a gradual enhancement of models’ capacity to capture complex causal structures while still being principally interpretable and maintaining robustness against corpus-specific details. This effectively enhances approximation power while controlling model bias and variance error, positioning multiplicative terms as a straightforward yet powerful tool to bolster the generalizability of interpretable models, especially for complex phenomena.

Our analysis reveals a distinctive trade-off in model performance between classification and generalization tasks, where configurations excelling in one often underperform in the other. For instance, the inclusion of nonlinear interactions benefited classification performance but either did not improve or even reduced generalization performance (Figure 2). This suggests that the added complexity from nonlinear interactions, though advantageous for classification, may learn task-specific details that hinder generalization to human judgments. This trade-off underscores the importance of modeling constraints that lead models to slightly underperform or underfit in the classification task. The engineered feature set and the assumption of linear interactions serve as such constraints. By identifying relevant features and constraining the search space, these mechanisms mitigate genre- and corpus-specific biases while guarding against shortcut learning from irrelevant patterns in the training data. Such constraints embody a form of external theory-ladenness that shapes the learning process, but they do not necessitate an internal theory of the relationships that produce a phenomenon through causal mechanisms. Whether rooted in psycholinguistics for textual complexity, in physics for physics-informed models, or in medical practice for physician-build machine learning models,^42^^,^^43^^,^^44^ this type of theory specifies how models navigate the trade-off between complexity and interpretability, ensuring a balance between task-specific performance and overall generalizability.

Our methodology highlights the value of small-scale generalization-evaluation data. For training data, their size is principally important, as it allows for the mitigation of potential spurious and irrelevant relationships that would otherwise take precedence in the data.^21^ Conversely, for target data, size is not a similar requirement, as uncommon relationships can benefit the target task. Our results demonstrate that such corpus specifics in target data rather amplify the generalization requirements posed by the target task. They increasingly necessitate that models capture generic principles that also perform well on specifics from the target data. Crucially, this presupposes the validity of these generalization specifics. This validity can be, as in our study, experimentally obtained in highly controlled settings using triangulated measures, capturing a specific set of configurations and nuances not seen in the training data. Moreover, this can involve the observation of extreme and relatively uncommon yet important cases, such as in fault modeling or adversarial learning.^41^^,^^77^ Achieving this level of control and incorporating adversarial cases is more easily achieved at a small scale than the large scale needed for training. While the resulting datasets might be deemed too small for the specialization achieved through fine-tuning or re-training a model, they reinforce the imperative for a generic model to excel across diverse scenarios.

### Limitations of the study

The question remains whether another, less-interpretable model could challenge the presented findings. With a maximum prediction performance of *r* = 0.484 for BERT, our results also underscore the potential of opaque models. Nevertheless, it is unlikely that newer embedding models will offer substantial improvements. BERT has shown competitive performance in classifying texts at different complexity levels when compared with other LLMs in cross-corpus studies,^60^^,^^78^^,^^79^ even when included in interpretable architectures.^32^ Additionally, our methodology revealed considerable variability in predictions by ChatGPT (see methods, baseline III: ChatGPT), underscoring the inherently stochastic nature of generative LLMs.^80^ Another question remains about whether or not alternative training procedures could challenge the presented findings. Care was taken to prevent overfitting, particularly for FNNs, by including an early stopping mechanism. Furthermore, the training procedure incorporated specific constraints, such as a minimal weight decay of 1e−4 for regularization, aimed at enhancing the models’ generalizability. Nevertheless, this underscores that the results should be interpreted along with the training procedures involved.

While this study provides valuable insights into the accuracy-interpretability trade-off and generalization performance for textual complexity modeling, its findings are inherently tied to the specific case studied. The results are bounded by the models, data, and training procedures employed, including the focus on textual data and specific linear (GLM and PLM) and nonlinear (FNN, Vec2Read, and ChatGPT) model types. Notably, a key characteristic of the case is the lack of near-exhaustive training data, contrasting LLMs. This characteristic is common to real-world applications, where labeling costs are high, observational data are noisy, and scaling is not feasible. In such scenarios, the demonstrated benefit of interpretable models may extend to similar modeling challenges with restricted training data. Another key characteristic is the data shifts encountered: predicting human judgments from binary class labels (label shift) and generalizing across genres, topics, and text lengths (covariate shift). While these shifts reflect common challenges in modeling human judgments and behavior and a degree of subjectivity is inherent even in human-in-the-loop labeling processes, their specifics will vary across domains. These limitations leave open the question of whether and to what extent the findings presented here—on the accuracy-interpretability trade-off, generalization performance, and utility of multiplicative interactions—translate to other domains and tasks.

### Conclusion

The rise of deep learning has revolutionized machine learning, driving breakthroughs across fields from text classification to image recognition and language modeling. By leveraging large datasets and raw inputs, data-intensive models have bypassed extensive feature engineering, making it possible to address previously intractable tasks. Yet, this shift has also amplified long-standing challenges, particularly around interpretability and generalization. Despite over 25 years of research, the interpretability-accuracy trade-off and generalization challenges remain central, underscoring their enduring relevance in machine learning.

This study offers a comprehensive comparison between interpretability and generalizability. By systematically varying interpretability and complexity, it evaluated models’ ability to generalize. Our approach combines a large-scale training dataset with a smaller, controlled dataset, enabling us to assess generalization to human judgments. This setup introduces a full data shift: differences in measures, tasks, and raters lead to label shift, while domain differences in genre, topics, and text length contribute to covariate shift. Unlike transfer learning or fine-tuning approaches that rely on task-specific re-learning, this design necessitates generic models that can capture universal features and relationships, enabling effective generalization without re-training.

Our findings add to a limited yet expanding body of evidence on the comparative effectiveness of human-interpretable models against opaque ones.^15^ Interpretability in this study was achieved by incorporating external theoretical insights on human processing difficulty into the feature set, combined with a generalized linear model. While these interpretable models showed lower test accuracy than their complex counterparts, they significantly outperformed them in generalizing to human judgments. Interestingly, incrementally adding linear, multiplicative interactions consistently improved generalization performance while maintaining principal interpretability. This enhancement may explain the discrepancy with earlier comparisons,^45^ as multiplicative interactions enhance interpretable models’ approximation power while maintaining robustness. These results suggest that while the accuracy-interpretability trade-off may apply to test accuracy, it does not necessarily extend to generalization, underscoring that interpretability and generalizability can instead complement each other.

## Methods

The experimental procedure followed a 2×2+3 setup:

• 2 distinct feature sets (embedding and engineered) × 2 classification heads (linear and nonlinear) +
• 3 baseline models (“rudimentary”; see Table 1 and Figure 1).

The evaluation encompassed the following:

(1) A train-test performance assessment with dataset I, which served as an objective ground truth on which to learn and compare different models.
(2) A generalization evaluation with dataset II, which allowed us to correlate the model’s output with subjective ratings of processing difficulty. This evaluation was the primary objective of this study, aiming at validating the inferred models on their ability to transfer to a distinctive genre and increasingly subjective appraisals.

Within each experimental condition (see Table 1), diverse model variations were trained, spanning a range in model depth and model parameter count. This allowed an in-depth exploration of the models’ behavior across both various dimensions of model complexity and two distinctive tasks.

### Dataset I: EnSiWiki2020

The model of textual complexity was estimated from pairs of English Wikipedia and Simple English Wikipedia articles (see supplemental note C). Both Wikipedia versions have clear guidelines and review processes. This dataset was constructed utilizing the oldest articles on Wikipedia, ensuring the selection of mature pairs, which are more likely to conform to their intended levels of quality and complexity, making this dataset ideal as an objective ground truth for modeling textual complexity in combination with the large selection of matured articles.

### Dataset II: Processing fluency

Participants’ interest was evaluated using data from a self-paced reading study. Before reading, participants rated their topical familiarity for up to 5 topics associated with each article. Articles were subsequently grouped into 3 counter-balanced blocks based on topical familiarity and randomized within each block. This grouping served to control for known effects of familiarity on interest. Following theorizing on interest as governing consumptive behavior (termed “liking”), whether the consumption is and will remain rewarding, rather than approach behavior (termed “wanting”),^81^ the articles were truncated at 1,200 characters, The study design provided a controlled setting with triangulated measurements, ensuring the reliability and validity of our data for assessing the extent to which textual complexity extends to human interest.

### Feature set I: Psycholinguistic effects

The first feature set highlights core effects on processing difficulty, grounded in psycholinguistic findings. Key effects and their definitions are reviewed below, with supporting paradigms detailed in supplemental note D.

#### Word length

Longer words require additional time for perceptual recognition,^53^ leading to extended fixation durations when reading^82^ with concurrent effects on naming tasks^51^ and lexical decision tasks.^52^ Word length is formalized in two indexes of length N, character count and a phonetically relevant syllable count:

(Equation 1) $$\begin{array}{cc} I^{a}= & \;\left\|w\right\|,\text{with}\;w=x_{1}x_{2}\ldots x_{N}\in \Sigma \text{,}\\ I^{b}= & \;\left\|w\right\|,\text{with}\;w=x_{1}x_{2}\ldots x_{N}\in S\text{,} \end{array}$$

with w denoting a word.

#### Semantic neighborhood density

Whereas dense distant neighborhoods facilitate word recognition, dense near neighborhoods can impede it.^83^ We formalize the neighborhood of a word based on the node degree A of a word in a semantic lexicon *W*,^84^ defined as the number of nodes reached within n=1…5 steps of a word x:

(Equation 2) $$\begin{array}{ccc} {\text{II}}_{n} & =\log\left|A_{n}\left(x\right)\right|\text{,}\;\text{with} & \\ A_{n}\left(x\right) & =A_{n-1}\left(x\right)\cup \left\{\phi \in W|r\left(\phi ,\phi^{\prime }\right),\phi^{\prime }\in A_{n-1}\left(x\right)\right\} & \text{for}\;n\geq 1\\ A_{0}\left(x\right) & =\left\{\phi \in W|x\in \phi \right\}\text{,} & \end{array}$$

where ϕ denotes a set of synonyms and $r\left(\phi ,\phi^{\prime }\right)$ is a Boolean function indicating whether there is any relationship between synset ϕ and synset $\phi^{\prime }$. A logarithm applied to $A_{n}\left(x\right)$ and the Hirst-St Onge method (see supplemental note E) for $r\left(\phi ,\phi^{\prime }\right)$ restrain the exponential growth of related synsets.

#### Word entropy

Within a meaningful context, repeated words or word sequences reduce first fixation and gaze duration, reflecting faster word recognition.^85^ Entropy gives an inverse measure of word repetition. Given a text X with words $x_{1}x_{2}\ldots x_{N}$ from vocabulary *V*, we define the conditional entropy for sequences (n-grams) of length n=1…4 defined within a sliding window of length m applied over X:

(Equation 3) $$\begin{array}{cc} {\text{III}}_{n}^{a}= & \frac{1}{N-m}\sum_{i=m}^{N}H_{n}\left(x_{i-m+1}\ldots x_{i}\right)\;\text{with}\;x\in V,\text{with}\;\\ H_{n}\left(X\right)= & -\sum_{j=n}^{N}p\left(x_{j-\left(n-1\right)}\ldots x_{j}\right)\;\log_{2}\;p\left(x_{j}|x_{j-\left(n-1\right)}\ldots x_{j-1}\right)\text{.} \end{array}$$

#### Semantic entropy

Words are better recognized when embedded in a semantically related local context.^85^ We calculate the entropy of topic vectors $\vec{\tau }$ as a means of quantifying semantic context within a sliding window over the terms $X=x_{1},x_{2},\ldots ,x_{N}\in V$:

(Equation 4) $$\begin{array}{cccc} {\text{III}}^{b}= & \;\frac{1}{N-m}\sum_{i=m}^{N}H\left(\sum_{x_{i-m+1}}^{x_{i}}{\vec{\tau }}_{x}\right) & \;\text{with}\; & x_{1}x_{2}\ldots x_{N}\in V\text{,}\\ H\left(\vec{\tau }\right)= & -\sum_{t\in \vec{\tau }}p\left(t\right)\log_{2}\;p\left(t\right) & \;\text{with}\; & p\left(t\right)=\frac{t}{{\left|\vec{\tau }\right|}_{1}},\text{and}\\ {\vec{\tau }}_{x}= & \;\left[t_{x1}t_{x2}\ldots t_{xn}\right]\text{,} & & \end{array}$$

where n denotes the number of topic dimensions, $t_{xd}$ the topic weight for term x on dimension d, and p(t) is the probability of topic $t\in \vec{\tau }$.

#### Dependency length

Within a sentence, words are connected to each other by syntactical links. The length of these links is a primary determinant of the speed and accuracy of their resolution.^86^ Distance effects can be observed through both a slowdown and an increase in regressive saccades at the region of dependency resolution.^87^ Given a sentence with a set of dependencies d∈D each spanning a sequence of words $d=x_{1}x_{2}\ldots x_{N}\in V$, the mean dependency length is given by

(Equation 5) $$\text{IV}=\frac{1}{\left|D_{s}\right|}\sum_{d\in D}\log_{10}\left\|x_{2}\ldots x_{N}\in d\right\|$$

A log transformation is included as a standard way to normalize count data and make it suitable for a linear classifier.

#### Word probability

The more unlikely a (subsequent) word, the higher its surprisal, and the higher the processing effort it incurs. Word predictability affects online processing, most notably self-paced reading time, first-pass gaze duration, and pupil size,^88^ and depends on its n preceding words:

(Equation 6) $$V_{n}=\frac{1}{N}\log\prod_{i=1}^{N}p\left(x_{i}|x_{i-n}\ldots x_{i-1}\right)\;\text{with}\;x_{1}x_{2}\ldots x_{N}\in V\text{,}$$

which gives the geometric mean of log probabilities of n-grams, following conventions,^88^ at the sentence level.

#### Cohesion

Cohesive texts are characterized by expressions being “about the same thing.” Except for highly knowledgeable readers, cohesion heightens comprehension and reduces reading time.^89^ We specify a lexical and semantic index of local cohesion over n=1…4 foregoing sentences in a text of $s_{1}\ldots s_{N}$ sentences:

(Equation 7) $$\begin{array}{l} {\text{VI}}_{n}^{a.b}=\frac{1}{N-1}\sum_{i}^{N}\sum_{j}^{n}{\text{sim}}^{a.b}\left(s_{i},s_{i-j}\right)\;\text{for}\;i-j\geq 1\text{,}\\ {\text{sim}}^{a}\left(s_{1},s_{2}\right)=\left|\left\{r\in R|r\in s_{1}\wedge r\in s_{2}\right\}\right|\;\text{or}\\ {\text{sim}}^{b}\left(s_{1},s_{2}\right)=\frac{\vec{\tau }\left(s_{1}\right)\cdot \vec{\tau }\left(s_{2}\right)}{\left\|\vec{\tau }\left(s_{1}\right)\right\|\left\|\vec{\tau }\left(s_{2}\right)\right\|},\text{with}\\ \vec{\tau }\left(s\right)=\frac{1}{\left\|s\right\|}\sum_{x\in s}\vec{\tau_{x}}\text{,} \end{array}$$

where ${\text{sim}}^{a}$ defines referential relatedness as the existence of a shared referent r∈R between sentences and ${\text{Sim}}^{b}$ defines semantic relatedness as the cosine similarity between sentence topic vectors $\vec{\tau }\left(s\right)$ (see also Equation 4).

#### Connectives

Connectives, such as “moreover,” “after,” and “because,” are linguistic markers that indicate discourse relations. The presence of connectives typically leads to faster processing times for information following a coherence marker^90^ and reduces re-reading times,^91^ especially for difficult texts.^92^ Let A be terms identified as connectives, either causal $A^{c}$ or noncausal $A^{n}$; we specify the frequency of connectives in a text $X=x_{1}x_{2}\ldots x_{N}\in V$:

(Equation 8) $$\begin{array}{cc} {\text{VII}}^{a}= & \frac{\left\|x_{1}x_{2}\ldots x_{N}|x\in A^{c}\right\|}{N}\;\text{with}\;x_{1}x_{2}\ldots x_{N}\in V\;\text{and}\\ {\text{VII}}^{b}= & \frac{\left\|x_{1}x_{2}\ldots x_{N}|x\in A^{n}\right\|}{N}\;\text{with}\;x_{1}x_{2}\ldots x_{N}\in V\text{.} \end{array}$$

The high linguistic variety in connective markers necessitates sophisticated NLP solutions for their detection, which we discuss in supplemental note F.

### Feature set II: Embeddings

The second feature set encompasses word embeddings, a powerful technique in NLP. Word embeddings learn a representation of words through the resolution of surrogate tasks. This task is typically a variant on a Cloze test, where the model learns to fill in missing words in a given window of surrounding words. This seemingly unrelated task prompts the model to discern and encode semantic and linguistic relationships between words. Consequently, words that share similar contexts tend to be positioned closer to each other in the embedding space, reflecting their linguistic and semantic affinity.

Their ability to learn intricate structures of language makes word embeddings ideal for readability modeling. Notably, the BERT contextual embedding model has emerged as one of the most effective approaches for modeling textual complexity.^60^^,^^78^^,^^79^ BERT consists of, among others, 12 embedding layers offering complementary representations of a word. Lower layers are primarily associated with surface-level features such as sentence length, while intermediate to high layers encode a rich hierarchy of syntactic and semantic information.^69^ Upper layers, in particular, perform well on textual complexity classification tasks,^69^ supporting their value as text representation for complexity modeling.

Based on these insights, we establish the subsequent feature set, encompassing all dimensions d=1…768 within the BERT model:

(Equation 9) $$\begin{array}{cc} {\text{emb}}^{d}= & \;\text{the}\;\text{extent}\;\text{of}\;a\;\text{word}\;x^{\prime }s\;\text{influence}\;\text{on}\;\text{dimension}\;d\\ & \;\text{within}\;\text{the}\;11^{\text{th}}\;\text{layer}\;\text{of}\;\text{BERT.} \end{array}$$

The feature set is computed by averaging word embeddings across words within a given text. This customary method of aggregation is enabled by the vector space properties of embedding spaces, which permit algebraic manipulation. Furthermore, a sliding window is applied to circumvent the input window size restrictions of 512 tokens (see supplemental note G).

### Model I: Generalized linear model

The generalized linear model (GLM) assumes a linear association between the response variable and the predictor variables, allowing for the estimation of coefficients that quantify these relationships. This facilitates the interpretation of how changes in predictor variables influence the response. Second- and third-order interactions between input features were included in the model to vary model depth, while the number of terms was restricted to control model complexity.

To yield an unbiased estimation of classification performance on dataset I, a 5-fold cross-validation loop was executed. After performance evaluation, the model was re-trained on the full dataset for subsequent generalization evaluation on dataset II. Within each training iteration, the following processing pipeline was conducted:

(1) Data preprocessing: missing values were removed, and minor class imbalances were rectified to ensure equiprobable classes.
(2) Feature scaling: normalization via root-mean-square deviation ($\text{RMSD})/\bar{x}$, as is common with GLMs.
(3) Feature set expansion: by the inclusion of first- to $d^{\text{th}}$-order interaction terms.
(4) Feature selection: from the expanded feature set, the top p terms were chosen after F-score ranking.
(5) Correlation-based pruning: to counter colinearity issues, features exhibiting correlation coefficients above *r* > 0.9 were eliminated.
(6) Classification: a GlmNET model with Lasso regularization was trained on the pruned feature set.^93^ Lasso regularization is ideal for extensive feature spaces, as it regularizes model terms toward zero, effectively eliminating irrelevant features. Furthermore, a logistic or log-odds transformation was included for binary classification problems.

The cross-validation loop was run for depth levels *d* = 1, 2, and 3. The inclusion of interaction terms led to an expansion of the feature space, culminating in a peak of 7.52e−7 third-order terms for feature set II. To address the ensuing computational challenges and mitigate overfitting risks (see supplemental note H), the top p terms were selected through ranking based on the F score. The F test assesses the discriminatory power of individual features in distinguishing between classes. Its univariate nature and linear time complexity make it particularly well suited for handling large feature sets. For feature Set I, the total numbers of terms p were constrained to 50,60,…350 at depth level 2 and 100, 200, ..., 2,700 at depth level 3. For feature set II, p was constrained to 1,000, 2,000, ..., 30,000 at both depth levels. This methodological step ensures that the most informative interaction terms are incorporated, thereby likely enhancing the effectiveness of the classification process. The combination of depth values d and term limits p resulted in a total of 120 unique configurations that were evaluated. Using the packages glmnet, mlr3, and caret, the processing pipeline was implemented in R.

### Model II: FNN

FNN is a standard deep learning architecture where information flows in one direction: from the input layer, through the hidden layers, to the output layer. Operating through multiple layers of interconnected nodes, or neurons, the FNN integrates information from neurons in preceding layers through a nonlinear activation function, thereby allowing the network to capture complex interactions between input features.

Nested resampling was implemented to tune, train, and evaluate a variety of FNN configurations. The nested resampling comprised (1) an inner loop, executed via 5-fold cross-validation, and (2) an outer loop, involving an 80%−20% train-test split, which were applied for each model under evaluation as follows:

(1) Hyperparameter tuning via the inner loop, applied to the train split, namely the learning rate (lr_rate=1e−5…1e−1), weight decay (weight_decay=1e−4…1e−1), dropout rate (dropout=0,0.1,0.2,0.3,and0.4), and activation function (tanh, ReLU, softmax, and sigmoid) (see supplemental note I).
(2) Performance evaluation on the test split.
(3) Generalization evaluation on the full dataset, resulting in the final model.

Each FNN was trained with a batch size of 128 using binary cross-entropy as loss function combined with the logits (sigmoid) function optimized for binary classification. The Adam optimizer, a robust learning algorithm, was coupled with an early stopping algorithm to proactively enhance model performance and generalization, guarding against overfitting (see supplemental note I). Furthermore, preprocessing addressed minor class imbalances and scaled the input features by their mean and standard deviation. This processing pipeline was constructed on pytorch in Python.

FNN training, following the nested resampling protocol, was applied across a range of architectures. The FNN architecture was varied on depth (d=1,2,and3) and width (n=4,8,…,164 for d>1) of layers. Furthermore, at d>1, dropout layers were included after each hidden layer. In total, 166 distinctive configurations were evaluated across both datasets, ensuring a thorough exploration of model behavior and performance under diverse architectures.

### Baseline I: PLMs

In addition to the 2 feature sets and classification models, 3 baseline models were employed. These baseline techniques integrate features and classification heads into a single model. Baseline I employs PLMs to estimate the likelihood that a text adheres to the modeled language. This estimation relies on the trigram assumption through the use of n-grams, which simplifies estimations by assuming that the probability of a word depends on n preceding words. These models have been instrumental in readability modeling, where n-gram probabilities are learned from texts representing varying reading levels, effectively distinguishing among multi-class readability grades.^47^^,^^94^ Unlike neural language models that utilize hidden layers and nonlinear activation functions, e.g., tanh,^95^ given the additive nature of probabilities, n-gram PLMs can be viewed as linear models. PLMs nevertheless capture interactions between words in n-grams, which allows them to capture $n^{\text{th}}$-order multiplicative dependencies between words from language data.

PLMs were trained using 3-fold cross-validation to ensure unbiased performance estimates. Per iteration, two models were trained: one for English and one for simple English. A class prediction was based on the log likelihood ratio between the two models. After cross-validation, each model was re-trained on the full EnSiWiki2020 dataset. The regular English model was subsequently used for generalization evaluation through correlating the log probability for an observed text against subjective ratings, predicting the likelihood that a text was generated by the complex PLM. The use of logscale is in line with Smith and Levy,^88^ who showed its superiority in predicting word-level reading time. PLMs were trained using the IRSTLM toolkit.^96^ For all PLMs, the Witten-Belt smoothing method was applied. In total, 25 PLMs were evaluated with n-grams (1–5) and varying vocabulary sizes, replacing beyond-vocabulary words with POS tags for enhanced model generalization^68^ (see supplemental note J).

### Baseline II: Vec2Read

For baseline II, the state-of-the-art readability model Vec2Read was used.^58^^,^^97^ Vec2Read employs rudimentary inputs with a deep learning architecture to estimate the readability of a given text. Its inputs are words, POS tags,^68^ character-word skip-gram embeddings by FastText,^98^ and a set of morphological tags extracted using SyntaxNet.^99^ The model’s architecture involves a multi-layer recurrent neural network, where each input is processed through a bidirectional long short-term memory (LSTM) layer. Its multi-layeredness and recurrence make this model able to not only generate feature representations from its inputs but also discern complex interactions both within and across its layers. For modeling, it uses a multi-layer recurrent neural network with bidirectional LTSM layers for each of its inputs and a single recurrence layer. Its ability to cover complex interactions allows for this model to be classified as “deep” (see Table 1).

Vec2Read was trained with a 0.01 learning rate and 100 epochs and tested on EnSiWiki2020. An 80% training-20% testing split was used. The resulting model attained an accuracy of 97.09%, a precision of 0.974, a recall of 0.967, and an F2 of 0.971. Given the resource-intensive nature of the training process, no variations were explored concerning the number of input features.

### Baseline III: ChatGPT

For baseline III, ChatGPT 3.5 was employed. As an LLM, ChatGPT has demonstrated proficiency in generating texts at different reading levels. However, given the extent of training data, it was impractical to fine-tune ChatGPT or similar LLMs. Consequently, ChatGPT was solely tasked, without re-training, to predict the reading level associated with each target text.

The employed prompt is given in supplemental note K. Predictions were elicited for both a labeled and a numeric scale, with the numeric scale demonstrating superior efficacy. 3× per target text, the specified prompt was presented, and the resultant values were averaged across the 3 predictions. The averaging process exhibited enhanced predictive performance compared to any singular prediction.

## Resource availability

### Lead contact

Any additional information required to reanalyze the data reported in this paper is available from the lead contact, Egon L. van den Broek (vandenbroek@acm.org), upon reasonable request.

### Materials availability

This study did not generate new materials. Datasets used for model training and evaluation are taken from existing data sources: Wikipedia and van der Sluis and van den Broek.^67^ These datasets are available on FigShare.^65^^,^^66^

### Data and code availability

Our source code is publicly available in a GitHub repository at https://github.com/fsluis/textual-complexity and has been archived at Zenodo: https://doi.org/10.5281/zenodo.14359835.^100^ This repository includes code for feature extraction and model training. Original data generated in this study, including intermediate and final results related to feature extraction and model training, have been deposited in FigShare: https://doi.org/10.6084/m9.figshare.25676394.^101^

## Acknowledgments

The Dutch Organization for Scientific Research (NWO) is gratefully acknowledged for funding the IPPSI-KIEM project Adaptive Text-Mining (ATM) (project no. 628.005.006). E.L.v.d.B. is also thankful to the UU-HONDA project, a cooperation between the Honda Research Institute in Japan and Utrecht University in the Netherlands.

## Author contributions

Conceptualization, F.v.d.S. and E.L.v.d.B.; data curation, F.v.d.S.; formal analysis, F.v.d.S.; funding acquisition, F.v.d.S. and E.L.v.d.B.; investigation, F.v.d.S. and E.L.v.d.B.; methodology, F.v.d.S. and E.L.v.d.B.; project administration, F.v.d.S. and E.L.v.d.B.; resources, F.v.d.S. and E.L.v.d.B.; software, F.v.d.S.; validation, F.v.d.S. and E.L.v.d.B.; visualization, F.v.d.S.; writing – original draft, F.v.d.S.; writing – review & editing, F.v.d.S. and E.L.v.d.B.

## Declaration of interests

The authors declare that they have no competing interests.

## Declaration of generative AI and AI-assisted technologies in the writing process

The authors used ChatGPT-4o for language refinement and editing assistance during the preparation of this work. The content was subsequently reviewed and edited by the authors, who take full responsibility for its final version.

## Supplemental information

Document S1. Figures S1 and S2, Table S1, and supplemental notes

Document S2. Article plus supplemental information
