# Supplementary material for: Model interpretability enhances domain generalization in the case of textual complexity modeling
Source: Patterns (N Y). 2025 Feb 6;6(2):101177. doi: 10.1016/j.patter.2025.101177 (PMC11873011; doi:10.1016/j.patter.2025.101177)
Supplement: Document S1. Figures S1 and S2, Table S1, and supplemental notes [file mmc1.pdf]

Patterns, Volume *6*

Supplemental information

Model interpretability enhances domain generalization in the case of textual complexity modeling

Frans van der Sluis and Egon L. van den Broek

Supplemental Notes

Interpretability Benefits Generalizability: The Case of Modeling Textual Complexity

Frans van der Sluis and Egon L. van den Broek

Patterns
December 28, 2024

This document includes supplemental notes, providing detailed insights, methods, and background information beyond the article's main text. The notes contain the following sections:

A AI readability

B Models' linearity

C EnSiWiki2020 details

D Experimental paradigms of processing difficulty

E Hirst-StOnge neighborhood size

F Feature implementation details

G BERT stepwise traversal

H Feature space inflation

I Hyperparameters and control mechanisms

J Probablistic language models' hyperparameters

K ChatGPT prompt

Followed by 56 references used in this supplemental notes.

A AI readability

Various tasks and datasets are employed in automatic readability detection. Typical tasks are text classification, rating prediction, and Cloze comprehension tests. other corpora, though not all available in the public domain. Notable data sources include the Common Core Appendix B (168 documents, grade levels 2 – 12) [S1], WeeBit (6 388 documents, 6 grade levels) [S2], CommonLit Ease of Readability (CLEAR) corpus (4 724 excerpts, linear ranking) [S3], and Wikipedia (up to 262 918 documents, binary levels) [S4, S5]. The diversity among these tasks and datasets poses a challenge in determining the overall state-of-the-art approaches. However, recent task- and data-specific performance may still be examined. Following a similar categorization as offered by Francois (2015) [S6] of "AI readability", three approaches to readability detection will be reviewed: *i)* lexical-syntactic models; *ii)* structure-cognitivist models with either linguistically-motivated or cognitively-motivated features, and; *iii)* non-expert models.

Lexical-syntactic models capture word frequencies as well as syntactic features, such as through word or Part-Of-Speech (POS) n-grams or sentence parse tree height. In particular the mapping of word frequencies to grade levels necessitates the availability of a representative and sufficiently large training corpus. Statistical language models have proven successful in classifying texts of varying degrees of complexity. In Collins-Thompson and Callan [S7] performance ranged from 63% to 67% when trained on as many as 12 grade levels and tested on 6 grade levels using smoothed unigrams. In Schwarm and Ostendorf [S8] classification accuracy peaked at 79% for distinguishing 5 grade levels whilst adding four syntactical features in addition to lexical language models using a Support Vector Machine (SVM) classifier. In Petersen and Ostendorf [S9], classification accuracy reached 63.18% using lexical-syntactic features on 4 grade levels using a SVM classifier. The dependency of a lexical model on a representative training corpus has either been mitigated by more loosely tying the (language) models to its raw inputs [S7] or by generating genre-specific language models [S10]. Within genres, the maximum correlation with a 5-point readability scale was $r = .817$ [S10]. Either way, this dependency necessitates to specialize lexical-syntactic models for particular genres and populations [S6].

Structuro-cognitivist models typically use linguistically-motivated features, benefiting from the availability of contemporary systems for Natural Language Processing (NLP). These models consider all dimensions of a text: lexical, syntactic, semantic, discourse, and even pragmatic aspects [S6]. Feng et al. [S11] combined a total 273 linguistically-motivated features to achieve a classification accuracy of 74.01% on a Weekly Reader data set with 4 grade levels using Logistic Regression Model (LRM) and SVM classifiers. Ding et al. [S12] achieved 83.72% accuracy over 5 imbalanced classes from the Common Standard Corpus with a Random Forest classifier. The CAREC model [S13] achieved a correlation of $r = .537$ with crowd-sourced readability judgements for 600 excerpts from Wikipedia using stepwise regression from an initial 63 to a final 13 features. Finally, Xia et al. [S14] achieved a classification accuracy of 80.3% and correlation of $r = .900$ on a modified version of the WeeBit corpus using a wide range of linguistically-motivated features with a SVM classifier. These models typically start out with several hundreds of features before feature selection. Their results suggests a strong performance for such all-encompassing, linguistically-motivated models of readability.

Whereas most structuro-cognitivist models are primarily linguistically motivated, several systems appeal to a cognitive motivation. Coh-Metrix is a well-known system, applying cognitively-inspired indexes to indicate the cohesion of a text [S15]. Applied to readability research, Crossley et al. [S16] shows how a regression model based on three criteria of the Coh-Metrix system correlates highly ($r = .925$) with Cloze test results for 32 academic reading texts, in comparison to $r = .691$ for the Dale-Chall formula. Another system is DeLite [S17]. It uses 48 morphological, lexical, syntactic, and semantic cognitively-motivated indicators to increase its content validity.

Compared to the traditional Flesch–Kincaid formula, DeLite's readability predictions correlated more highly with participants' difficulty ratings ($r = .43$ vs. $r = .53$, respectively) for German administrative texts. Another system, TextEvaluator, employs 43 cognitively-motivated features and achieves correlations of up to $r = .81$ for readability of informational texts and of $r = .78$ for literary texts [S18] with a linear regression model. And, Vajjala and Meurers [S2] employed a total of 46 features, derived from second language acquisition research, achieving 93.3% accuracy on five classes from the WeeBit corpus using a multi-layer perceptron. These results suggest a possible performance gain for psychologically valid models that employ fewer, generic features of readability.

A recent development is by non-expert models which employ little feature engineering. Whereas feature engineering requires some level of (linguistic or cognitive) expertise and applies some form of filter on the data, non-expert models use network or graph-based machine learning to infer a data representation from raw inputs. Cha et al. [S19] achieves correlation of $r = .825$ on 5 grade levels for the Common Core Standards corpus using character n-gram word embeddings in combination with K-Means clustering and a Support Vector Regression (SVR) model. Vec2Read [S20] achieves 91.8% binary accuracy on a Wikipedia dataset using character n-gram word embeddings and morphological and syntactic (POS tags) embeddings with a recurrent neural network (Vec2Read). And, Jiang et al. [S21] achieves $F1 = 92.38$ average binary accuracy for 4 grade levels of an English New Concept textbook corpus using tf-idf features with a graph propagation method. These models typically use auxillary tasks and corpora to train their data representations. For example, word embeddings are typically trained using a skipgram task in which a neural network predicts a withheld word. Such word embeddings and neural networks, possibly in combination with linguistically-motivated features [S22, S23, S24, S25], often achieve state-of-the-art performances making them a promising avenue for readability modelling.

The current overview of "AI readability" showed an evolution in modelling approaches, ranging from language models based on rudimentary representations (word n-grams), via extensive feature engineering, to recent word embeddings and neural networks. Nonetheless, strong variations in performance exist within each approach, likely due to variations between data sets and measurements used for evaluation. Even though many studies share the same source of documents (e.g. Weekly Reader, Wikipedias) their selections typically differ. Moreover, concerns about the validity of these corpora have been raised, as they result from the judgements of a small set of experts with often low levels of inter-rater agreement [S6]. This limits the generalisability of resulting models, both to other expert-assessed corpora and to subjective, user-generated ratings. It highlights a tradeoff between domain specificity and generalisability, where the former relies on representative and sufficient training data and the latter on generic principles of readers' processing difficulty.

B Models' linearity

Probablistic Language Models and Generalized Linear Models can be classified as linear models due to their reliance on the additive combination of model terms. In these models, predictions are generated through the linear summation of features weighted by coefficients. Even though these models, through the inclusion of multiplicative terms, can introduce some level of non-linearity, their ability to capture more complex non-linear patterns remains limited. In contrast, neural networks transcend these limitations using their multi-layer architecture and non-linear activation functions. These components allow neural networks to capture a wide range of complex nonlinear patterns in addition to multiplicative interactions. The division between nonlinear and linear methodologies in Table 1 reflects this distinction between the linear and non-linear nature of these models, whilst acknowledging that linear models can capture some degree of

nonlinear relationships.

C EnSiWiki2020 details

The EnSiWiki2020 is based on the Wikipedia dump dated April $1, 2020$, processed to plain text using JWPL [S26], with the removal of templates and links to files and images. The final selection of articles is described in Table S1. Articles were filtered on having a minimal length of 30 words and 5 sentences as determined by a Lucene tokenizer [S27] and Stanford CoreNLP sentence splitter [S28]. Article pairs were established through following language links, which linked Simple English articles to their corresponding English Wikipedia counterparts. This linkage occasionally resulted in multiple Simple English articles being connected to a single English Wikipedia article, contributing to the slight difference in set sizes in Table S1. The oldest $50\,000$ articles were selected that were neither a special, redirect, or disambiguation page. This selection was based on the age rank of the pairs. The pair age rank was determined by the mean age rank of a pair's articles, as determined by ranking both the English and Simple English articles on their first revision date. A non-parametric ranking was preferred over an arithmetic mean of first revision date given the different growth rates of the Wikipedia versions.

		Counts			
Data set	Selection*	Documents	Sentences**	Words**	Characters**
EnWiki	Articles	24 943	176.61	3 943,23	19 762,73
	Sections	138 668	31.17	697,21	3 493,79
	Paragraphs	352 606	7.49	159,92	799,27
SiWiki	Articles	25 057	21.79	335,74	1 582,74
	Sections	39 078	12.86	197,37	930,96
	Paragraphs	40 208	7.04	102,22	479,81
The Guardian		18	11.11	203.61	968,56

Note. *After filtering on texts with a minimum of 5 sentences and 30 words. *After truncation at 1 200 characters. **Average count per document.

Table S1: Descriptive statistics per data set and selection.

D Experimental paradigms of processing difficulty

Building generic models of textual complexity requires capturing fundamental principles of reading. These principles are actively investigated in controlled settings through diverse experimental tasks, each using distinct measures of processing difficulty [S29]. This involves tasks in which word, sentence, and discourse features are compared to readers' ability to process and comprehend the presented information. Analogous to auxiliary tasks for generating embedding models, these tasks can be considered auxiliary to the task of predicting textual complexity. This section will detail the most relevant tasks employed, providing a framework for an external theory of textual complexity.

Typical word-level tasks are the lexical decision and naming tasks [for a review, see S30]. In the lexical decision task participants choose whether a word is an actual word or rather a nonword. The decision speed on this task is affected by a range of word-level characteristics and has contributed to theory development on visual word recognition. In the naming task the rapid

pronunciation of words and pseudowords is tested. Variants on these tasks include (masked) priming, where the relation between a (subconscious) prime and its target are explored. These tasks have been successful in evaluating features of and relations between words on decision speed, such as word length and frequency and semantic relatedness [e.g., S31, S32, S33], and given rise to various models of visual word recognition [S30].

Typical sentence-level tasks involve self-paced reading tasks with sentences that involve some level of ambiguity or complexity, such as the case with garden path sentences [for a review, see S34]. During reading, online measures of reading times and fixation durations are able to reveal subtle changes in language processing using eye-tracking. An alternative task is for participants to complete sentence fragments. When combined with offline measures, such as final comprehension questions or judgments, these tasks explore 'linking hypotheses' between processing and comprehension difficulty. These tasks have successfully shown effects of within-sentence dependencies and word-sequence probabilities on sentence processing difficulty [e.g., S35, S36, S37] and led to related, contrasting theories of sentence processing [S38, S39].

Discourse-level tasks typically involve comprehension tests after self-paced reading of a text [for a review, see S40]. Comprehension is assessed using various comprehension tests, including immediate recall tests, Cloze tests, and tests with multiple choice or open-ended questions. In combination with online measures including reading time and eye-tracking measures, these measures allow the investigation of inferences made during processing. Optimal understanding is thought to be achieved when a coherent mental representation of a text is formed by making inferencing from and integrating individual discourse elements and sentences. The creation of this mental representation is an interplay between the information provided by the text and the background knowledge of a user [S41]. Text comprehension tasks have revealed, among other findings, the interplay between prior knowledge, textual cohesion, and the use of connectives on comprehension [e.g., S42, S43, S44].

The relationship between processing difficulty and comprehension is somewhat equivocal: less-than-optimal word processing may already be sufficient for good discourse comprehension [S45]. This indicates a distinction between processing difficulty and comprehension, suggesting that word-level effects may contribute to processing difficulty without necessarily affecting comprehensibility. As demonstrated by the broad applications of a metric of textual complexity, comprehensibility is just one possible consequence of processing difficulty. Learning outcomes, interest [S46], and relevance judgments [S47] all relate to the ease or difficulty with which a text can be processed. This underscores the need for generalizable models of textual complexity that extend to other corpora and tasks [e.g., interest prediction S46]. To this end, the described experimental paradigms and tasks can be viewed as 'auxiliary tasks' to the target task of predicting textual complexity. Utilizing the findings from these tasks as a foundation for a feature set effectively provides an explicit form of external theory-ladenness, ensuring the relevance of textual features to the phenomenon of textual complexity.

E Hirst-StOnge neighborhood size

With function $r(\varphi, \varphi')$ in Equation 2 we limit the growth of related synsets following the Hirst-StOnge measure of semantic relatedness. This method constrains the paths that can be traversed between nodes to a set of allowable patterns, such as first upward (e.g., hypernymy) followed by horizontal (e.g., similar to) steps [see Fig 13.2a; S48]. The intuition behind these constraints is that both path length and direction changes influence semantic relatedness, the value of which is confirmed through comparisons with human judgements [S49].

F Feature implementation details

For features Dep (dependency length) and $\mathrm{Coh}^{\mathrm{ref}}$ (referential cohesion), the Stanford CoreNLP annotator was used with the tokenize, ssplit, pos, and depparse components for dependency parsing and an additional lemma, ner, and coref component for statistical coreference resolution [S50].

For feature LogPr_n, the CommonCrawl 5-gram language model is used [S51]. Derived from over 9 billion Web pages of the CommonCrawl corpus, making it one of the largest conventional models currently available.

For features $\mathrm{Ent}^{\mathrm{sem}}$ (semantic entropy) and $\mathrm{Coh}^{\mathrm{sem}}$ (semantic cohesion), an Explicit Semantic Analyses (ESA) topic model was created from a Lucene [S27] index of English Wikipedia. Stopwords were removed and all terms were lemmatized using the Snowball stemmer [S52]. This led to a total of 3 734 199 articles or topic dimensions after L2-normalization and pruning [see S53, p. 453]. Each dimension in the ESA model corresponds to a Wikipedia article, rendering the dimensions readily interpretable as topics. This high dimensionality, coupled with the interpretability of its dimensions, sets ESA apart from traditional embedding spaces. Specifically, the calculation of entropy benefits from the granularity afforded by the vast number of dimensions in this high-dimensional topic space. Topical entropy is influenced by both the number of dimensions activated and the heterogeneity of their activation. In a high-dimensional topic space, there's a greater variance in the number of dimensions activated, enabling more precise and fine-grained activation of specific topics. This characteristic offers a particular advantage for calculating topical activation and dispersion for feature $\mathrm{Coh}^{\mathrm{sem}}$ (semantic entropy).

For feature Con (connectives), the AltLex parser [S54] was employed. This parser identifies a wide variety of discourse markers from a parallel sentence Wikipedia corpus by matching phrases with known explicit connectives to parallel phrases that instead contain an alternative lexicalization (AltLex). This method achieves an automatic detection accuracy of 79.58%, navigating the linguistic variation in markers. Explicit markers, though rare, can be identified with high precision, whereas implicit relations, being more prevalent, are harder to automatically recognize. AltLexes fall between these extremes, offering a solution for automatic detection due to their commonality and significant linguistic variety.

G BERT stepwise traversal

A constraint inherent in embedding models, such as BERT, pertains to the scope of context they can encompass. This scope is confined to a span of 512 words, less than the typical length of our training texts (Wikipedia articles). Truncating articles at 512 tokens would amount to a substantial loss of information, which has been related to performance losses [S5]. This limitation is resolved by a sliding window approach involving overlapping windows. As depicted in Figure S2, this approach involves the stepwise traversal of an area of interest consisting of 256 tokens. Preceding and subsequent tokens are added to form a contiguous input window of size 512. This approach guarantees that each token in a text is included in an area of interest once. The inclusion of appropriate leading and trailing context enables BERT to generate accurate and contextually informed word embeddings.

H Feature space inflation

The inclusion of interaction terms results in the expansion of the feature space. The number of feature combinations reaches 325 second-order and 2600 third-order combinations for Feature

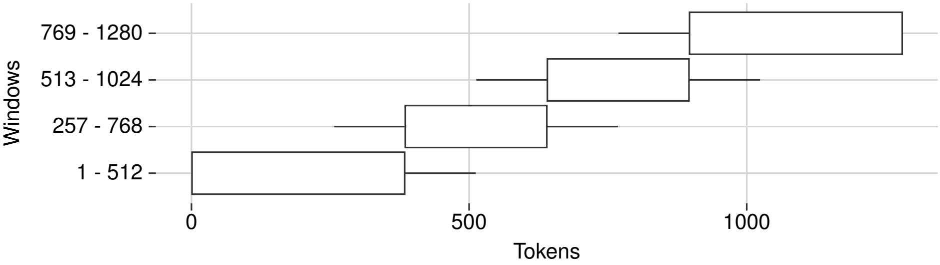

Figure S2: Sliding window approach applied to accommodate for BERT's input size restriction, illustrated for a text of 1280 tokens. Boxes indicate the area of interest used for further processing. Whiskers indicate leading and trailing tokens ensuring properly contextualized word embeddings.

Set I, and 294 528 and $7.52\mathrm{e}{-7}$ combinations for Feature Set II. The latter upper limit surpasses the total number of observations (50 000), raising concerns about the potential for spurious relationships. Additionally, the computational complexity $\mathcal{O}(np^2+p^3)$ of regression modeling, where n represents the number of observations and p signifies the number of features, escalates rapidly beyond reasonable computational capacity.

I Hyperparameters and control mechanisms

For FNN (Feedforward Neural Network) training, the following hyperparameters and control mechanisms were used:

Adam optimizer The Adam optimizer was used as an adaptive learning algorithm that self-tunes during training. It combines the first moment (mean) and second moment (uncentered variance) of gradients to adaptively adjust the learning rate during training, promoting swift convergence and robustness [S55]. These characteristics make it a standard choice for optimization tasks.

Early stopping Test performance was validated every 10 training iterations (epochs) starting from epoch 20. If, in a window of 50 epochs, test performance did not increase, the best performing model from that window was selected and training was stopped. These stopping criteria were chosen after initial inspection of validation error curves, suggesting a patience of 50 epochs was needed for consistent and robust results. Early stopping serves to prevent overfitting by terminating training when a divergence between improvements in training and validation errors emerges [S56].

Learning rate The initial learning rate set for the Adam optimizer was considered a hyperparameter. The values of *lr_rate* $= 1\mathrm{e}{-5} \ldots 1\mathrm{e}{-1}$ were evaluated. A range of learning rates was employed to empirically strike a balance between training speed and smooth and consistent convergence of the training process.

Weight decay The weight decay hyperparameter controls the magnitude of the L2 regularization term within the Adam optimizer. L2 regularlization constrains model parameters ensuring that all network nodes contribute to some extent. Contrary to the L1 (Lasso) regularlization term customary with GLM, this regularlization does not eliminate terms from the model. The values of *weight_decay* $= 1\mathrm{e}{-4} \ldots 1\mathrm{e}{-1}$ were evaluated.

Dropout rate The dropout rate dictates the proportion of neurons that are temporarily deactivated during training iterations. This technique introduces a degree of randomness, preventing specific neurons from becoming overly dependent on others and promoting more robust and generalized network representations. To enhance the capacity to generalize to unseen data, dropout values of *dropout* $= 0, 0.1, 0.2, 0.3, 0.4$ were tuned.

Activation function A neural network derives its nonlinear capacity from its activation function. Various non-linear activation functions, including the hyperbolic tangent (tanh), rectified linear unit (ReLU), softmax, and sigmoid were explored for this hyperparameter. Each function imparts distinct assumptions regarding the relations between neurons in the model, enabling it to capture intricate patterns within the data.

J Probablistic language models' hyperparameters

For probablistic language models, n-grams provide an intuitive way to control the 'depth' of the model, whilst the number of included words offers further control of the number of parameters. A total of 25 distinct PLMs were evaluated, spanning n-gram ranges from 1 to 5, and vocabulary extents of 250, 500, 1000, 10000, and 100000. The vocabulary selection process followed an information-gain ranking methodology as outlined in [S9]. This ranking strategy assigns higher priority to terms that exhibit a high frequency within a specific class while maintaining relative rarity in other classes. This approach is designed to capture terms that offer significant discriminatory power across readability classes. Terms that exceeded the prescribed vocabulary limits were replaced with their corresponding POS tags. This supplementary procedure incorporates syntactic features into the Probablistic Language Model (PLM) against only a marginal expansion of the model's parameter count. Early developments on Language Model (LM)-based readability classification showed that this integration of syntactic features has a positive impact on classification performance and enhances the model's ability to generalize to previously unseen texts [S8, S9].

K ChatGPT prompt

The prompt given to ChatGPT was:

> "*Below is an instruction that describes a task, paired with an input that provides further context. Write a response that appropriately completes the request. Instruction: Detect the reading level of the text. Write the reading level on a scale of "very difficult", "difficult", "fairly difficult", "standard", "fairly easy", "easy", "very easy". Also, give the reading level on a continuous scale from 0-1, where 0 denotes very easy and 1 very difficult.*
> *Input: "[text]"*
> *Reading level (label): ?*
> *Reading level (0-1): ?*"

The placeholder "[text]" was substituted by a target text.

References

S1. Vajjala, S., and Meurers, D. (2014). Assessing the relative reading level of sentence pairs for text simplification. In: Proceedings of the 14th Conference of the European Chapter of the Association for Computational Linguistics. Association for Computational Linguistics (288–297). doi:10.3115/v1/E14-1031.

S2. Vajjala, S., and Meurers, D. (2012). On improving the accuracy of readability classification using insights from second language acquisition. In: Proceedings of the Seventh Workshop on Building Educational Applications Using NLP. NAACL HLT '12 Association for Computational Linguistics (163–173).

S3. Crossley, S., Heintz, A., Choi, J. S., Batchelor, J., Karimi, M., and Malatinszky, A. (2023). A large-scaled corpus for assessing text readability. Behav. Res. Methods *55*, 491–507. doi:10.3758/s13428-022-01802-x.

S4. Madrazo Azpiazu, I., and Pera, M. S. (2020). Is cross-lingual readability assessment possible? J. Assoc. Inf. Sci. Technol. *71*, 644–656. doi:10.1002/asi.24293.

S5. Martinc, M., Pollak, S., and Robnik-Šikonja, M. (2021). Supervised and unsupervised neural approaches to text readability. Comput. Linguist. *47*, 141–179. doi:10.1162/coli_a_00398.

S6. François, T. (2015). When readability meets computational linguistics: A new paradigm in readability. Revue Française de Linguistique Appliquée *20*, 79–97. doi:10.3917/rfla.202.0079.

S7. Collins-Thompson, K., and Callan, J. (2005). Predicting reading difficulty with statistical language models. J. Assoc. Inf. Sci. Technol. *56*, 1448–1462. doi:10.1002/asi.20243.

S8. Schwarm, S. E., and Ostendorf, M. (2005). Reading level assessment using support vector machines and statistical language models. In: Proceedings of the 43rd Annual Meeting on Association for Computational Linguistics. ACL '05 Association for Computational Linguistics (523–530).

S9. Petersen, S. E., and Ostendorf, M. (2009). A machine learning approach to reading level assessment. Computer Speech & Language *23*, 89–106. doi:10.1016/j.csl.2008.04.003.

S10. Kate, R. J., Luo, X., Patwardhan, S., Franz, M., Florian, R., Mooney, R. J., Roukos, S., and Welty, C. (2010). Learning to predict readability using diverse linguistic features. In: Proceedings of the 23rd International Conference on Computational Linguistics. COLING '10 Association for Computational Linguistics (546–554).

S11. Feng, L., Jansche, M., Huenerfauth, M., and Elhadad, N. (2010). A comparison of features for automatic readability assessment. In: Proceedings of the 23rd International Conference on Computational Linguistics: Posters. Association for Computational Linguistics (276–284).

S12. Ding, H., Zhong, Q., Zhang, S., and Yang, L. (2022). Text difficulty classification by combining machine learning and language features. In: Xie, Q., Zhao, L., Li, K., Yadav, A., and Wang, L., eds. Advances in Natural Computation, Fuzzy Systems and Knowledge Discovery: Proceedings of the ICNC-FSKD 2021 vol. 89 of *Lecture Notes on Data Engineering and Communications Technologies*. Springer International Publishing (1055–1063). doi:10.1007/978-3-030-89698-0_108.

S13. Crossley, S. A., Skalicky, S., and Dascalu, M. (2019). Moving beyond classic readability formulas: new methods and new models. J. Res. Read. *42*, 541–561. doi:10.1111/1467-9817.12283.

S14. Xia, M., Kochmar, E., and Briscoe, T. (2016). Text readability assessment for second language learners. In: Proceedings of the 11th Workshop on Innovative Use of NLP for Building Educational Applications. Association for Computational Linguistics (12–22). doi:10.18653/v1/W16-0502.

S15. Graesser, A., McNamara, D., Louwerse, M., and Cai, Z. (2004). Coh-Metrix: Analysis of text on cohesion and language. Behav. Res. Methods *36*, 193–202.

S16. Crossley, S., Greenfield, J., and McNamara, D. (2008). Assessing text readability using cognitively based indices. TESOL Quarterly *42*, 475–493.

S17. Vor der Brück, T., Hartrumpf, S., and Helbig, H. (2008). A readability checker with supervised learning using deep indicators. Informatica *32*, 429–435.

S18. Sheehan, K. M., Kostin, I., Napolitano, D., and Flor, M. (2014). The TextEvaluator tool. Elementary School Journal *115*, 184–209. doi:10.1086/678294.

S19. Cha, M., Gwon, Y., and Kung, H. T. (2017). Language modeling by clustering with word embeddings for text readability assessment. In: Proceedings of the 2017 ACM on Conference on Information and Knowledge Management - CIKM '17. ACM Press (2003–2006). doi:10.1145/3132847.3133104.

S20. Azpiazu, I. M., and Pera, M. S. (2019). Multiattentive recurrent neural network architecture for multilingual readability assessment. Trans. Assoc. Comput. Linguist. *7*, 421–436. doi:10.1162/tacl_a_00278.

S21. Jiang, Z., Gu, Q., Yin, Y., Wang, J., and Chen, D. (2019). GRAW+: A two-view graph propagation method with word coupling for readability assessment. J. Assoc. Inf. Sci. Technol. *70*, 433–447. doi:10.1002/asi.24123.

S22. Lee, B. W., Jang, Y. S., and Lee, J. (2021). Pushing on text readability assessment: A transformer meets handcrafted linguistic features. In: Moens, M.-F., Huang, X., Specia, L., and Yih, S. W.-t., eds. Proceedings of the 2021 Conference on Empirical Methods in Natural Language Processing. Association for Computational Linguistics (10669–10686). doi:10.18653/v1/2021.emnlp-main.834.

S23. Imperial, J. M. (2021). BERT embeddings for automatic readability assessment. In: Mitkov, R., and Angelova, G., eds. Proceedings of the International Conference on Recent Advances in Natural Language Processing (RANLP 2021). INCOMA Ltd. (611–618).

S24. Lee, J., and Vajjala, S. (2022). A neural pairwise ranking model for readability assessment. In: Muresan, S., Nakov, P., and Villavicencio, A., eds. Findings of the Association for Computational Linguistics: ACL 2022. Association for Computational Linguistics (3802–3813). doi:10.18653/v1/2022.findings-acl.300.

S25. Liu, F., and Lee, J. (2023). Hybrid models for sentence readability assessment. In: Kochmar, E., Burstein, J., Horbach, A., Laarmann-Quante, R., Madnani, N., Tack, A., Yaneva, V., Yuan, Z., and Zesch, T., eds. Proceedings of the 18th Workshop on Innovative Use of NLP for Building Educational Applications (BEA 2023). Association for Computational Linguistics (448–454). doi:10.18653/v1/2023.bea-1.37.

S26. Zesch, T., Müller, C., and Gurevych, I. (2008). Extracting lexical semantic knowledge from Wikipedia and Wiktionary. In: Proceedings of the Sixth International Conference on Language Resources and Evaluation (LREC'08). European Language Resources Association (ELRA).

S27. Hatcher, E., Gospodnetic, O., and McCandless, M. Lucene in Action. Second revised ed. Manning Publications Co. (2010).

S28. Manning, C., Surdeanu, M., Bauer, J., Finkel, J., Bethard, S., and McClosky, D. (2014). The Stanford CoreNLP natural language processing toolkit. In: Bontcheva, K., and Zhu, J., eds. Proceedings of 52nd Annual Meeting of the Association for Computational Linguistics: System Demonstrations. Baltimore, Maryland: Association for Computational Linguistics (55–60). doi:10.3115/v1/P14-5010.

S29. Rueschemeyer, S.-A., and Gaskell, M. G., eds. The Oxford handbook of psycholinguistics. Oxford University Press (2018). doi:10.1093/oxfordhb/9780198786825.001.0001.

S30. Rastle, K. (2018). Visual word recognition. In: Rueschemeyer, S.-A., and Gaskell, M. G., eds. The Oxford handbook of psycholinguistics. Oxford University Press (47–70). doi:10.1093/oxfordhb/9780198786825.013.3.

S31. McGinnies, E., Comer, P., and Lacey, O. (1952). Visual-recognition thresholds as a function of word length and word frequency. J. Exp. Psychol. *44*, 65–69. doi:10.1037/h0063142.

S32. Balota, D. A., Cortese, M. J., Sergent-Marshall, S. D., Spieler, D. H., and Yap, M. J. (2004). Visual word recognition of single-syllable words. J. Exp. Psychol. Gen. *133*, 283–316. doi:10.1037/0096-3445.133.2.283.

S33. New, B., Ferrand, L., Pallier, C., and Brysbaert, M. (2006). Reexamining the word length effect in visual word recognition: New evidence from the English lexicon project. Psychon. B. Rev. *13*, 45–52. URL: 10.3758/BF03193811. doi:10.3758/BF03193811.

S34. MacDonald, M. C., and Hsiao, Y. (2018). Sentence comprehension. In: Rueschemeyer, S.-A., and Gaskell, M. G., eds. The Oxford handbook of psycholinguistics. Oxford University Press (170–196). doi:10.1093/oxfordhb/9780198786825.013.8.

S35. Boston, M. F., Hale, J., Kliegl, R., Patil, U., and Vasishth, S. (2008). Parsing costs as predictors of reading difficulty: An evaluation using the Potsdam sentence corpus. Journal of Eye Movement Research *2*. doi:10.16910/jemr.2.1.1.

S36. Liu, H., Xu, C., and Liang, J. (2017). Dependency distance: A new perspective on syntactic patterns in natural languages. Phys. Life Rev. *21*, 171–193. doi:10.1016/j.plrev.2017.03.002.

S37. Rajkumar, R., van Schijndel, M., White, M., and Schuler, W. (2016). Investigating locality effects and surprisal in written English syntactic choice phenomena. Cognition *155*, 204–232. doi:10.1016/j.cognition.2016.06.008.

S38. Smith, N. J., and Levy, R. (2013). The effect of word predictability on reading time is logarithmic. Cognition *128*, 302–319. doi:10.1016/j.cognition.2013.02.013.

S39. Gibson, E., Futrell, R., Piantadosi, S. P., Dautriche, I., Mahowald, K., Bergen, L., and Levy, R. (2019). How efficiency shapes human language. Trends Cogn. Sci. *23*, 389–407. doi:10.1016/j.tics.2019.02.003.

S40. Ferstl, E. C. (2018). Text comprehension. In: Rueschemeyer, S.-A., and Gaskell, M. G., eds. The Oxford handbook of psycholinguistics. Oxford University Press (196–216). doi:10.1093/oxfordhb/9780198786825.013.9.

S41. Kintsch, W., and van Dijk, T. A. (1978). Toward a model of text comprehension and production. Psychol. Rev. *85*, 363 – 394. doi:10.1037/0033-295X.85.5.363.

S42. Haberlandt, K. (1982). Reader expectations in text comprehension. In: Language and Comprehension vol. 9 of *Advances in Psychology*. Elsevier (239–249). doi:10.1016/S0166-4115(09)60055-8.

S43. Graesser, A. C., Singer, M., and Trabasso, T. (1994). Constructing inferences during narrative text comprehension. Psychol. Rev. *101*, 371–395. doi:10.1037/0033-295x.101.3.371.

S44. Kleijn, S., Pander Maat, H. L., and Sanders, T. J. (2019). Comprehension effects of connectives across texts, readers, and coherence relations. Discourse Process. *56*, 447–464. doi:10.1080/{0163853X}.2019.1605257.

S45. Long, D. L., Wilson, J., Hurley, R., and Prat, C. S. (2006). Assessing text representations with recognition: The interaction of domain knowledge and text coherence. J. Exp. Psychol. Learn. Mem. Cogn. *32*, 816–827.

S46. van der Sluis, F., and van den Broek, E. L. (2023). Feedback beyond accuracy: Using eye-tracking to detect comprehensibility and interest during reading. J. Assoc. Inf. Sci. Technol. *74*, 3–16. doi:10.1002/asi.24657.

S47. Collins-Thompson, K., Bennett, P. N., White, R. W., de la Chica, S., and Sontag, D. (2011). Personalizing web search results by reading level. In: Berendt, B., de Vries, A., Fan, W., Macdonald, C., Ounis, I., and Ruthven, I., eds. Proceedings of the 20th ACM International Conference on Information and Knowledge Management. CIKM '11 ACM (403–412). doi:http://doi.acm.org/10.1145/2063576.2063639.

S48. Miller, G. A. (1995). WordNet: A lexical database for English. Commun. ACM *38*, 39–41. doi:http://doi.acm.org/10.1145/219717.219748.

S49. Budanitsky, A., and Hirst, G. (2006). Evaluating WordNet-based measures of lexical semantic relatedness. Comput. Linguist. *32*, 13–47. doi:10.1162/coli.2006.32.1.13.

S50. Clark, K., and Manning, C. D. (2015). Entity-centric coreference resolution with model stacking. In: Zong, C., and Strube, M., eds. Proceedings of the 53rd Annual Meeting of the Association for Computational Linguistics and the 7th International Joint Conference on Natural Language Processing (Volume 1: Long Papers). Association for Computational Linguistics (1405–1415). doi:10.3115/v1/P15-1136.

S51. Buck, C., Heafield, K., and van Ooyen, B. (2014). N-gram counts and language models from the Common Crawl. In: Calzolari, N., Choukri, K., Declerck, T., Loftsson, H., Maegaard, B., Mariani, J., Moreno, A., Odijk, J., and Piperidis, S., eds. Proceedings of the Ninth International Conference on Language Resources and Evaluation (LREC'14). European Language Resources Association (ELRA) (3579–3584).

S52. Porter, M. F. (2001). Snowball: A language for stemming algorithms. Available online at http://snowball.tartarus.org/texts/introduction.html [Last accessed on December 17, 2024].

S53. Gabrilovich, E., and Markovitch, S. (2009). Wikipedia-based semantic interpretation for natural language processing. J. Artif. Intell. Res. *34*, 443–498.

S54. Hidey, C., and McKeown, K. (2016). Identifying causal relations using parallel Wikipedia articles. In: Proceedings of the 54th Annual Meeting of the Association for Computational Linguistics (Volume 1: Long Papers). Association for Computational Linguistics (1424–1433). doi:10.18653/v1/P16-1135.

S55. Kingma, D. P., and Ba, J. (2014). Adam: A method for stochastic optimization. Preprint at arXiv. doi:10.48550/arxiv.1412.6980.

S56. Prechelt, L. (2012). Early stopping —but when? In: Montavon, G., Orr, G. B., and Müller, K.-R., eds. Neural networks: tricks of the trade vol. 7700 of *Lecture notes in computer science*. Springer Berlin Heidelberg (53–67). doi:10.1007/978-3-642-35289-8_5.
