# Supplementary material for: Model interpretability enhances domain generalization in the case of textual complexity modeling
Source: Patterns (N Y). 2025 Feb 6;6(2):101177. doi: 10.1016/j.patter.2025.101177 (PMC11873011; doi:10.1016/j.patter.2025.101177)
Supplement: Document S2. Article plus supplemental information [file mmc2.pdf]

Article

Patterns

Model interpretability enhances domain generalization in the case of textual complexity modeling

Highlights

- Interpretable models outperform deep ones in a human generalization task
- Data shifts reveal the limits of deep models on textual complexity tasks
- Linear interactions enhance generalization while maintaining transparency
- Findings challenge the interpretability-accuracy trade-off for out-of-distribution tasks

Authors

Frans van der Sluis,
Egon L. van den Broek

Correspondence

vandenbroek@acm.org

In brief

Deep learning dominates modern machine learning, but models often struggle to generalize when data shift, and their decision processes remain opaque. This study shows that interpretable models—enhanced with linear feature interactions—can outperform deep models. These findings highlight the potential of interpretable models to deliver both transparency and robust generalization, particularly in real-world tasks with limited training data, data shifts, and diverse evaluation contexts.

van der Sluis & van den Broek, 2025, Patterns *6*, 101177
February 14, 2025 © 2025 The Author(s). Published by Elsevier Inc.
https://doi.org/10.1016/j.patter.2025.101177

CellPress

Patterns

CellPress
OPEN ACCESS

Article

Model interpretability enhances domain generalization in the case of textual complexity modeling

Frans van der Sluis[1] and Egon L. van den Broek[2,3,*]
[1]Department of Communication, University of Copenhagen, Copenhagen, Denmark
[2]Cybernetics Group, Department of Information and Computing Sciences, Utrecht University, Utrecht, the Netherlands
[3]Lead contact
*Correspondence: vandenbroek@acm.org
https://doi.org/10.1016/j.patter.2025.101177

THE BIGGER PICTURE Machine learning has revolutionized text analysis, but this revolution has a price: deep learning sacrifices interpretability, making it hard, and often impossible, to understand how they work. At the same time, their ability to generalize to new tasks and domains remains a challenge, particularly when data shifts occur—changes in text genre, topic, and/or human judgment criteria. Our findings challenge the assumption that interpretability comes at the cost of performance and suggest that interpretable approaches offer unique advantages for modeling human judgments, particularly when training data are limited and/or generalization is required. We provide empirical evidence and a methodological framework to advance interpretable machine learning.

SUMMARY

Balancing prediction accuracy, model interpretability, and domain generalization (also known as [a.k.a.] out-of-distribution testing/evaluation) is a central challenge in machine learning. To assess this challenge, we took 120 interpretable and 166 opaque models from 77,640 tuned configurations, complemented with ChatGPT, 3 probabilistic language models, and Vec2Read. The models first performed text classification to derive principles of textual complexity (task 1) and then generalized these to predict readers' appraisals of processing difficulty (task 2). The results confirmed the known accuracy-interpretability trade-off on task 1. However, task 2's domain generalization showed that interpretable models outperform complex, opaque models. Multiplicative interactions further improved interpretable models' domain generalization incrementally. We advocate for the value of big data for training, complemented by (1) external theories to enhance interpretability and guide machine learning and (2) small, well-crafted out-of-distribution data to validate models—together ensuring domain generalization and robustness against data shifts.

INTRODUCTION

Data-intensive models have become the *de facto* standard for modeling and predicting a wide variety of phenomena. From speech recognition and visual object detection to drug discovery and genomics, machine learning techniques, particularly deep learning, consistently outperform conventional approaches.[1] In text analysis, neural models excel in classification tasks and significantly reduce perplexity in language modeling. This trend also extends to tasks like sentiment analysis, spam detection, authorship attribution, and—in the present case—textual complexity modeling.[2] The successes of (deep) machine learning techniques can be attributed to their ability to address phenomena that were previously thought intractable due to their sheer size or complexity, often exceeding the human capacity for comprehension.[3,4] This ability is enabled by the availability of near-exhaustive training data, representing all, or a substantial portion, of the conditions relevant to the examined phenomenon.[4] Crucially, deep neural networks attain these abilities without handcrafted feature representations; they can deduce a relevant representation directly from raw inputs such as pixels, characters, or words.[1,5] Their success signifies a broader trend in data-intensive modeling favoring larger datasets and raw inputs or, in other words, bigger data and smaller datums.[6]

Despite their impressive performance, data-intensive models face growing concerns related to interpretability,[7] explainability,[8] and generalizability,[9,10] especially as machine learning applications extend into real-world, high-stakes environments.[11,12]

© 2025 The Author(s). Published by Elsevier Inc.
This is an open access article under the CC BY license (http://creativecommons.org/licenses/by/4.0/).

The rise of deep learning has often meant sacrificing model transparency for accuracy, leading to limited insight into model behavior and decision-making processes—a drawback that has fueled the field of explainable artificial intelligence (xAI).[8,13] Simultaneously, reliance on vast datasets assumed to be independent and identically distributed (IID) (also known as [a.k.a.] ID, i.i.d., and iid) has overlooked real-world shifts in data distributions, leading to unexpected drops in model performance when tested out of distribution (OoD).[11,12] This challenge, also known as domain generalization,[9,10] emphasizes the importance of validating model robustness across diverse data domains, shifting the traditional train-validate-test paradigm to prioritize generalization under data shifts. Our study addresses these two critical aspects—interpretability and generalizability—through a comparative, empirical investigation in textual complexity modeling,[14] a domain where alignment with human understanding is essential and models face diverse data environments.

Interpretability

Contrary to the trend toward large, deep models is a recent shift toward interpretable models.[15–19] Central to this movement is the use of a few, inherently interpretable features combined with a simple, human-understandable model. This combination provides transparency by explaining which feature combinations produce an outcome and control by enabling users to specify which features are deemed (ir)relevant to the modeled phenomenon. The resultant interpretable models are believed to not only grant users the ability to make informed decisions regarding their trustworthiness[13,16,20] but also make it easier to identify and correct errors or biases,[21] leading to more robust and reliable systems.[22] For example, through selective feature design, interpretable models can avoid or mitigate biases in training data, such as textual genres or cultural stereotypes.[23] Theoretical grounding enhances interpretability by ensuring that input features are relevant and that model structures are logically consistent with the phenomenon being modeled. This shift aligns with Occam's razor, favoring models that are no more complex than necessary to represent a phenomenon effectively.[24]

A key factor enabling the use of simpler models is the Rashomon effect, which suggests that complex data, including human cognitive processes, can often be accurately modeled by many different models that achieve similar performance.[25] This concept, introduced by Breiman,[24] highlights that there can be a multitude of valid yet distinct models that fit the data well. However, even though the Rashomon effect suggests that simple, interpretable models can often perform as well as more complex ones, interpretable models frequently fall short in effectiveness. Data-intensive models, particularly those using deep learning, typically achieve state-of-the-art performance,[1] leading to an ongoing debate about the interpretability-accuracy trade-off.[15] This debate centers on not only the trade-off itself but also the difficulty of finding accurate, simple models. As Semenova et al.[26] observes, "It is almost always easier to find an accurate-but-complex model than an accurate-yet-simple model. Finding optimal, sparse, accurate models of various forms … is generally NP-hard. We often do not know whether the search for a simpler model will be worthwhile, and thus we do not go to the trouble of searching for one." This observation raises the question of which model we should choose and when it is worth pursuing simplicity.

Some studies challenge the interpretability-accuracy trade-off, showing that interpretable models can be competitive in certain conditions. In domains that lend themselves to feature engineering and tasks involving tabular data, comparisons between different classification heads on the same task suggest that interpretable models can achieve a comparable level of performance. For instance, Razavian et al.[27] showcased the comparable performance of a linear regression model to tuned random forests, gradient-boosted decision trees, and neural networks in a disease prediction task, Tollenaar and van der Heijden[28] and Zeng et al.[29] show that linear regression models produce equally accurate predictions of recidivism, and Kung and Yu[30] likewise show on-par performances of regression models for college success predictions. Conversely, few comparisons exist for non-tabular, textual data. Notable exceptions are Yadav et al.,[31] who demonstrated the competitive performance of interpretable Tsetlin machines (TMs) against various neural networks in a sentiment analysis task, and Singh et al.,[32] who showed the competitive performance of interpretable, linear models against large language models (LLMs) on text classifications tasks. A plausible explanation is that textual domains are less suitable for representation through simple models with a limited number of features. As a result, while such head-to-head comparisons are increasingly reported, especially in domains involving tabular data, they remain relatively uncommon in textual, non-tabular domains.

Generalizability

Performance benchmarks on identical datasets may, furthermore, not provide a comprehensive understanding of model efficacy. The reason is that typical validation methods, such as cross-validation and train-test splits, often fall short when it comes to evaluating a model's ability to generalize beyond its training data. Since these methods draw from the same distribution as the training set, they offer a limited view of the model's performance in new, unseen contexts. Without diverse and representative training data, models risk overfitting to noise (i.e., variance error) or lack the necessary information to learn the target function accurately (i.e., estimation error). In some cases, in-sample variations can even lead to shortcut learning, where models predict the right answer for the wrong reason[16] by relying on dataset artifacts rather than learning meaningful patterns.[33,34] To develop models that generalize robustly across different conditions, OoD validation, also known as domain generalization, is essential.[10] Domain differences between factors such as sampling location,[33] time, or—particularly in natural language processing (NLP)—variations in genre, writing style, and topic may lead to data shifts in feature distributions (i.e., covariate shifts). Domain generalization studies evaluate models' ability to handle these shifts, revealing the strengths and limitations of different modeling approaches in OoD situations.

In addition to OoD evaluations across different domains, human generalization evaluates models by directly comparing their outputs against human judgments. This practice, common in fields where model training and evaluation involve different tasks, often introduces both label shifts and covariate shifts, resulting in a full shift in data distributions. Label shifts arise because the measurement scales used for human validation differ from the labels used in training. Covariate shifts occur

CellPress
OPEN ACCESS

because the data used for human validation may differ in domain, context, or other properties from the training data. For instance, human generalization studies can capture nuances through graded, multi-faceted rating scales (e.g., sentiment strength[35]), evaluate model adaptability to complex inputs (e.g., adversarial examples[36]), contextualize evaluations for specific use scenarios (e.g., decision-making contexts[37]), and align models with the expertise and needs of diverse user groups (e.g., physicians[38]). Together, these practices broaden model validity across diverse inputs, nuances, contexts, and populations.[39] Due to the difficulty of obtaining valid and varied data through human studies, human judgments are typically too limited in size to serve directly as training data. Instead, they reveal areas where generic models must generalize effectively from training data without re-learning, demonstrating the model's ability to handle full shifts in data distributions (cf. van der Sluis et al.[14]).

Generalization studies favor model configurations that not only work well on training data but also extend to cross-domain or human validations. Possible workarounds are through transfer learning and fine-tuning approaches, wherein models re-learn specifics for each new dataset or task given the availability of sufficient data for re-learning. Alternatively, to create generic models that generalize without re-learning, model designs need to navigate two classical modeling trade-offs: the approximation-estimation and bias-variance trade-offs. The approximation-estimation trade-off balances a model's capacity to represent complex relationships (approximation) against the need for sufficient data to accurately estimate these relationships (estimation). The bias-variance trade-off addresses the inherent assumptions within a model's design (bias) vs. the model's sensitivity to variations within the training data (variance). Both the approximation error and bias error are caused by the choice of model. For instance, various techniques have been explored to reduce the sensitivity of (deep) machine learning models, including regularization, ensemble models, and adversarial learning.[40,41] Interpretability serves as one of such techniques, where the refined control over the (ir)relevance of features and the ability to inspect the relationships modeled provide a means to manage noise and inherent biases in training data.[21] This benefit of interpretability has been observed in fields such as physics-based and physician-built models,[42–44] where simpler, transparent models are applied reliably across diverse contexts, though it has not been consistently observed in other fields such as cancer transcriptomics.[45] Even though this provides additional motivation to pursue a generic, simple-yet-accurate model in generalization settings, the extent to which interpretability enhances generalizability for textual tasks and with human validation remains uncertain.

Textual complexity

Automatic readability detection, also known as textual complexity modeling, aims to assess and predict the difficulty of texts. Common tasks in this domain include classifying texts according to their complexity level or predicting expert and reader ratings of difficulty.[14] Textual complexity modeling plays an essential role in fields such as education,[46] content personalization,[47] and information retrieval,[48] where aligning text readability with audience capabilities and needs is important. Textual complexity can be considered a complex phenomenon, mirroring the intricacies of human language comprehension.[49] This complexity makes it unlikely that a purely theoretical causal structure can be derived, instead favoring data-intensive approaches. However, interpretability remains valuable, particularly in "educational contexts, where teachers or students may need to understand the factors that make a text difficult" (Collins-Thompson,[50] p. 105). As a complex phenomenon, textual complexity offers an ideal case to compare interpretable and advanced models.[14] A plethora of psycholinguistic studies (e.g., Balota et al.,[51] New et al.,[52] and McGinnies et al.[53]) has explored how specific text features, such as word familiarity and semantic priming, impact human processing difficulty, providing a basis for interpretability. Historically (for an overview, see supplemental note A), textual complexity has been modeled using interpretable methods, like linear regression with linguistically or cognitively motivated features.[54,55] However, this inherent complexity also favors advanced models capable of capturing nuanced relationships among features. Recent advancements in AI have underscored the success of deep models in classifying texts by complexity,[56–58] establishing a basis for comparative analyses.

The availability of ample training data for textual complexity modeling has been considered a challenge.[59] The intricacies of the phenomenon make it unlikely that exhaustive data will be available from which to induce a causal structure. In addition, these intricacies make it likely that any model needs to uncover higher-order interactions between input features, posing further requirements for the training data to cover all relevant variations within an inflated feature space. This reflects the approximation-estimation trade-off, where a model's limited representational capacity (approximation) and the finite, likely insufficient, training data (estimation) constrain the model's accuracy in capturing complex relationships fully. Current readability studies address these sampling requirements by employing data that are representative of populations, genres, document properties (e.g., language and length), and reading level.[50] Notable data sources include the Common Core Appendix B (168 documents, grade levels 2–12), WeeBit (6,388 documents, 6 grade levels), and Wikipedia (up to 2,62,918 documents, binary levels).[60] However, the use of a representative dataset results in models specialized according to a specific target.[59] Generalization studies confirm this specialization, revealing drops in performance for new yet similar datasets.[54–56,59] They underscore the challenge of capturing generic principles of textual complexity from training data, favoring generalization studies that test models across diverse domains of text.

This challenge is further compounded by a subjective component in textual complexity. Subjective differences become apparent from a characteristically low inter-rater agreement, even though typical readability datasets rely on expert assessments. These differences are simultaneously reflected in a reduction in model performance between similar and dissimilar experts[61] and between more objective and subjective measurements, even after re-training.[62] Such domain and human generalization studies highlight the variation that exists between and within individuals. Individual traits such as prior knowledge and reading proficiency combine with temporal cognitive and motivational processes to determine a reader's comprehension of a text[63] and, equally so, their experienced processing difficulty.

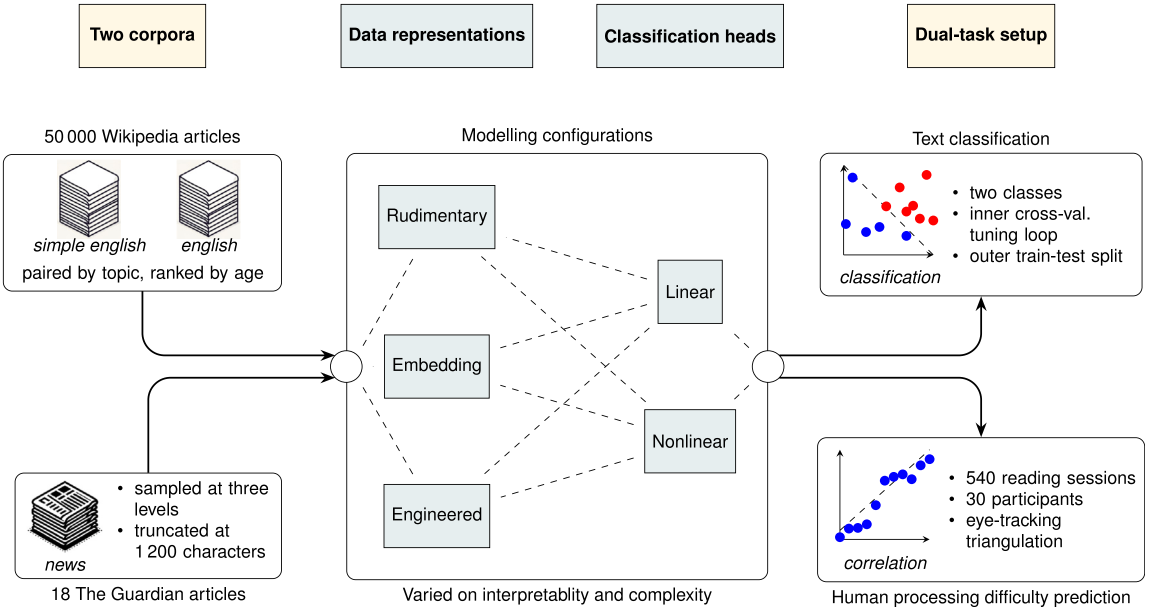

Figure 1. Schematic overview of study methodology
Lines illustrate flow of data, with dashed lines indicating evaluated modeling configurations. Paper stacks were generated using Dall-E on January 29, 2024.

This variation necessitates models to differentiate a generic component from an inherently individual, subjective component of textual complexity.[50] Ideally, a generalizable model of complexity goes beyond expert assessments to subjective judgments of processing difficulty, underscoring the human-centered challenge of aligning with diverse human experiences and understandings.

Research outline

Par excellence, textual complexity poses a data-intensive modeling challenge. Its foundation in the intricate processes of human comprehension makes it a complex phenomenon, highlighting the challenge of obtaining near-exhaustive training data. Furthermore, its partial subjectivity highlights the need for generalization studies that evaluate models on a subjective component. This imperative favors a generalization setup, recognized as a hallmark in modeling benchmarks.[64] To this end, this study introduces a dual-task setup for the comparison of various modeling approaches, detailed in Figure 1 and Table 1. The primary task is a text classification task in which models are trained and tested on their ability to classify texts according to two levels of complexity. The secondary task is a correlation task in which models are evaluated on their ability to predict human judgments of processing difficulty. The aim is for models to derive generic principles of textual complexity from training data that generalize to human judgments. This dual-task setup facilitates a comprehensive comparison, allowing us to assess modeling approaches based on their capacity to induce a generic and human-generalizable model.

For our training data, a dataset[65] consisting of 50,000 easy and difficult Wikipedia articles forms the foundation from which to infer generic principles of textual complexity. Articles were matched into pairs to mitigate the potential influence of the topic as a confounding variable. Furthermore, articles were selected based on their age to ensure a mature and likely high-quality sample. This strategic selection and pairing of articles is expected to help reveal relevant conditions and reduce the presence of irrelevant conditions in the training data. For our target data, we used a dataset[66] from a study involving 30 participants who appraised the processing difficulty of 18 (truncated) news articles across 540 reading sessions. Articles were sampled at the easiest, medium, and highest levels of complexity from a set of 14,856 articles using a preliminary complexity model. Subjective appraisals were obtained in a controlled laboratory environment and triangulated with eye tracking to ensure high-quality data, as reported in van der Sluis and van den Broek.[67] The relatively small size of the target data, where 30 readers appraise a sample of articles, contrasts with the large training dataset typically used, where few experts label many articles. The controlled environment and the number of participants make it likely that nuances in participants' reading experiences were captured. This setup introduces a label shift from binary complexity labels in Wikipedia training data to graded, reader-based appraisals of processing difficulty in the target data. Additionally, a covariate shift can be expected from domain differences in genre, topic, and article lengths between Wikipedia's encyclopedic content and the journalistic style of The Guardian articles.

Table 1 details the modeling configurations assessed in the dual-task setup. The experiment compares three types of data representations: (1) rudimentary representation, which remains close to the raw input (e.g., sequences of characters, words, and part-of-speech [POS] tags[68]), (2) embedding representation, derived from a learned feature representation using a (deep) language model (i.e., bidirectional encoder representations from transformers [BERT]), and (3) feature representation, founded on a formalization of reviewed psycholinguistic findings. All three representations capture some degree of syntactical, structural, and semantic aspects of text useful for modeling textual

Table 1. Modeling configurations evaluated within the dual-task setup

	Classification models	
Feature sets	Linear[a]	Nonlinear
Rudimentary[b]	PLM<br>3 variations	Vec2Read and ChatGPT[c]<br>2 models
Embedding	BERT and GLM<br>61 variations	BERT and FNN<br>83 variations
Engineered	PDfeat and GLM<br>59 variations	PDfeat and FNN<br>83 variations

Configurations include the feedforward neural network (FNN), probabilistic language model (PLM), and generalized linear model (GLM), with engineered features (PDfeat) and bidirectional encoder representations from transformers (BERT) embeddings. Within each configuration, diverse model variations are trained, spanning different model depths and parameter counts. In total, variations alongside baseline models account for 291 distinct models of textual complexity.

[a]Linear models are able to capture some degree of nonlinear relationships from training data; see supplemental note B.

[b]Baseline models with integral data representation.

[c]ChatGPT is only applied to the generalization task.

complexity. Rudimentary and embedding spaces implicitly capture these dimensions, with BERT reflecting a rich array of linguistic features.[69] The engineered feature set explicitly computes these aspects, grounded in well-established psycholinguistic findings and theoretical paradigms, which adds to the validity and establishes the interpretability of the features. These representations correspond to various configurations of the bias-variance trade-off, defining aspects of an external theory that frames the modeling problem.[4] The more explicit the representation, the more constrained the subsequent learning process, defining what features a model can learn and restraining the interaction space.

The experiment contrasts two model categories, detailed in Table 1: (1) linear models designed to capture linear relationships and n-degree multiplicative interactions within the data representation and (2) nonlinear models designed to capture nonlinear relationships within the data representation. Two aspects of the models are systematically varied for the embedding and feature representations: interaction depth (three levels) and model parameters (26–153,012 parameters). Interaction depth enables models to uncover higher-order interactions between input features, better equipping them to capture the understood intricate nature of textual complexity. The number of model parameters specifies the configurations of values a model should learn from the training data, which need to be appropriate given the amount of observations available for learning. Together, these aspects form different configurations of the approximation-estimation trade-off. A deep model can capture complex relationships, while a well-regulated model increases the likelihood of distinguishing relevant configurations from spurious ones. Of these configurations, only linear models with engineered features can be considered inherently interpretable, whereas other configurations might be explainable.[15]

The primary objective of this study is to systematically compare various modeling options to establish a generic and generalizable model of textual complexity. Through the systematic variation of feature sets and modeling approaches, we seek to assess the impact of interpretability and model complexity on the generalizability of resultant models. This comparative investigation adds to a limited yet expanding body of evidence on the effectiveness of interpretable models vs. opaque ones.[15] Additionally, the combination of a large dataset for training with a small, controlled experimental dataset for generalization creates an OoD evaluation. This setup challenges models to apply learned principles to new, unseen scenarios that differ along multiple dimensions. Genre (encyclopedic vs. news articles), tasks (text classification vs. processing difficulty), measurements (objective vs. subjective), and assessors (few experts vs. 30 readers) all differ between dataset I and dataset II. While finding an optimal, interpretable model is considered NP hard,[26] these differences introduce an additional, challenging full data shift for generalization. By studying interpretability and human generalization in tandem, our work contributes both empirical evidence and a methodological framework to the study of the interpretability-accuracy trade-off.

BACKGROUND AND RELATED WORK

Generalization, the ability of a model to perform well on new, unseen data, is a core objective in NLP. However, achieving robust OoD generalization remains challenging due to the diverse and dynamic nature of language data. NLP models often encounter a variety of data shifts—systematic changes in data distributions—that impact their ability to generalize effectively. Let x represent the input features and y denote the output or labels, and then Hupkes et al.[64] define covariate shifts when the input distribution ($p(x)$) changes between training and validation data (i.e., $p(x_{tst}) \neq p(x_{tr})$), but the conditional relationship $p(y|x)$ remains the same, allowing the evaluation of a model's ability to learn the underlying phenomenon. Label shift, on the other hand, happens when $p(y|x)$ changes due to variations in output distribution, such as inter-annotator disagreements or changes in tasks, affecting the model's ability to handle varying interpretations or labeling across contexts.[70] Full shift is the most extreme type of shift, where both the input and output distributions change simultaneously (i.e., $p(x_{tst}) \neq p(x_{tr})$ and $p(y_{tst}|x_{tst}) \neq p(y_{tr}|x_{tr})$).

Generalization research in NLP has developed several paradigms to evaluate model performance across data shifts: cross-task, cross-domain, and robustness generalization.[64] Cross-task generalization assesses a model's ability to apply its knowledge across multiple NLP tasks, typically through pretraining on a broad objective followed by fine-tuning on task-specific parameters. This method tests how well general linguistic knowledge can adapt to diverse tasks, with or without task-specific model adjustments.[71] Cross-domain generalization measures a model's adaptability to different types of text. Domains in NLP can vary by genre, formality, or topical focus, such as adapting a model trained on news articles to scientific literature.[72] It evaluates a model's robustness to diverse, naturally occurring language contexts. Robustness generalization focuses on a model's ability to avoid relying on spurious correlations within training data. This type of generalization targets unintended data shifts, such as annotation artifacts or demographic imbalances, ensuring that the model's predictions

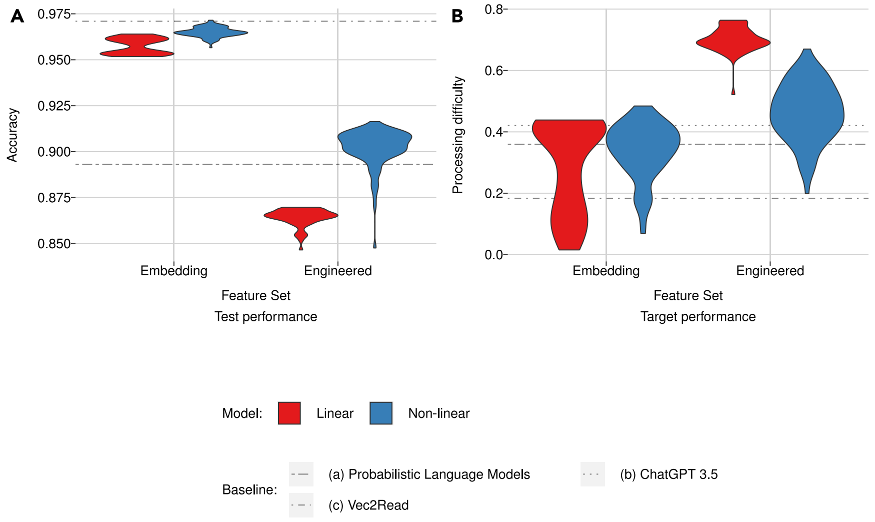

Figure 2. Distribution plots of models' performance per combination of feature set and model type

(A) Classification accuracy (Acc.) on the EnSiWiki2020 corpus.

(B) Models' correlation coefficient with processing difficulty (Proc. Diff.) as measured for The Guardian texts.

Baseline results are included as horizontal lines in both graphs when available. The best-performing baseline model is shown for probabilistic language models, ensemble performance for ChatGPT 3.5, and performance after 100 training epochs for Vec2Read.

align with the modeled phenomenon rather than superficial data patterns.[73] Of these, the first generalization paradigm mostly targets label shifts, whereas the others mostly cover covariance shifts.

In textual complexity research, several studies have investigated the generalizability of models across different corpora of text difficulty. Cross-domain experiments, which test models on unseen datasets, reveal performance declines due to covariate shifts. For instance, models trained on the WeeBit corpus and tested on Common Core standards showed correlation drops from r = 0.74 to 0.49,[55] from 0.92 to 0.69,[54] and from 0.900 to 0.730.[56] In the latter case, performance on the target corpus improved to r = 0.905 by expanding and mapping the feature space, though this adjustment requires access to target data for re-training.[56] Performance is generally more stable for models that include engineered features. For example, the regression model CAREC improved slightly from r = 0.571 on crowd-sourced evaluations to r = 0.580 on the CommonLit ease of readability (CLEAR) corpus.[74,75] Similarly, a hybrid model incorporating engineered features in a neural network showed only minor shifts, with performance varying from 0.685–0.699 to 0.639–0.683.[76] Interestingly, cross-language experiments within a similar domain also show relatively stable performance. For instance, models trained in English and tested on related corpora in different languages (English-French and English-Spanish) reported performance declines from r = 0.99 to 0.90–0.95.[57] These findings illustrate the varied effects of covariate shifts on readability models. Cross-domain shifts—such as changes in genre or topic—impact performance most significantly. While feature engineering or adaptation techniques, such as feature mapping, can help maintain model accuracy, these methods depend on the features used or the availability of target labels, complicating generalization to new, OoD datasets.

In addition to testing across different corpora, a few studies have examined the generalizability of models across varying measures of text difficulty. Cross-measurement performance typically involves comparing expert judgments and measurement scales. For instance, model performance drops from 73% to 59% when generalizing from experts with aligned judgments to those with more diverse opinions.[61] Likewise, when training on subjective ratings of text processing difficulty and testing on comprehension difficulty, correlations decrease from r = 0.683 to 0.557.[62] These substantial reductions in cross-measurement performance highlight the subjectivity involved in assessing reading difficulty (see introduction, textual complexity). This subjectivity leads to differing interpretations of text complexity, creating label shifts.

Our study extends the existing landscape of generalization research with a full shift study involving both label and covariate shifts. The covariate shift results from genre and topical differences: Wikipedia's encyclopedic content contrasts with the journalistic style of The Guardian articles, creating variability in topics, writing style, and text length. The label shift arises from moving from binary complexity labels in training to a graded measure of processing difficulty. While Wikipedia reflects diverse contributor expertise, the processing difficulty measure captures varied cognitive demands experienced by readers, shaped by their backgrounds and interests. Through a controlled experiment, this measure was triangulated with eye-tracking data (see Figure 1), offering a rigorous, OoD human experiment that precisely captures readers' experiences beyond simple labeling. Together, these shifts provide a robust foundation for evaluating the generalizability of different models of textual complexity.

RESULTS

A total of 65,640 feedforward neural network (FNN) and 12,000 generalized linear model (GLM) configurations were tuned, resulting in 166 FNN and 120 GLM models. The resulting configurations were subsequently trained and evaluated through cross-validation on dataset I (i.e., training or classification task) and evaluated using Pearson's correlation with processing difficulty for dataset II (i.e., target or generalization task). After tuning, a total of 61 GLM and 83 FNN models were trained on BERT embedding features and 59 GLM and 83 FNN models on PDfeat-engineered features, complemented with 5 baseline models including ChatGPT, 3 probabilistic language models (PLMs), and Vec2Read.

Models' performance

Figure 2 visually represents the performance distribution of models on both datasets, revealing two notable contrasts

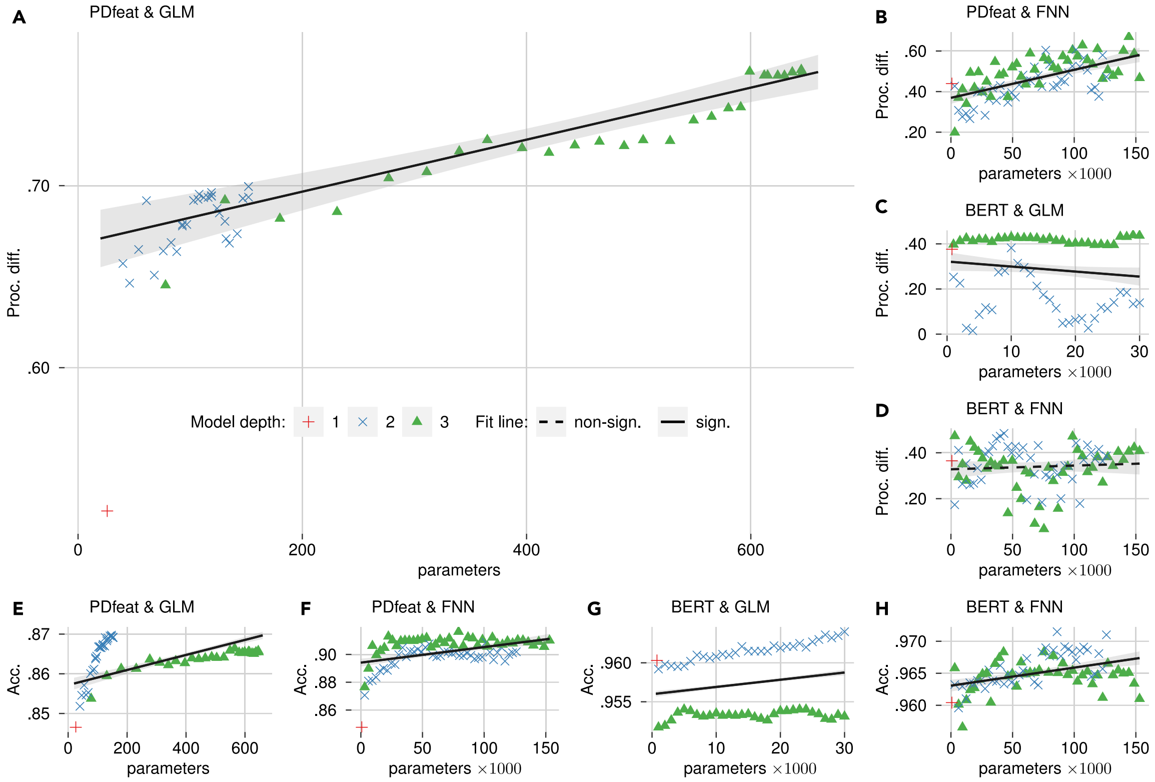

Figure 3. Models' performance summarized by the number of model parameters and model depth
(A–D) Models' correlation on the processing difficulty prediction task.
(E–H) Models' accuracy on the text classification task.
The number of parameters is derived from the number of input features and model complexity (see methods). Model depth is either the degree of multiplicative interactions between features for GLMs or the number of layers for FNNs. The fit lines, along with their 95% confidence intervals and statistical significance, illustrate the estimated marginal effect of parameters on performance within regression models (see Table 2).

with a distinctive mirror-like pattern. First, the figure shows that FNN models outperform GLM models in classification, whereas the reverse pattern appears for predicting processing difficulty. With embedding features, GLM and FNN models show little difference in generalization performance, but with engineered features, GLM models markedly outperform FNN models. Second, it illustrates that embedding features outperform engineered features in the classification task, while the reverse pattern emerges in predicting processing difficulty. In particular, the latter differences are by a large margin. This mirror-like pattern highlights a stark difference in how model and feature types perform across classification and generalization tasks.

Figure 2, furthermore, shows the performance of three baselines. On the text classification task, Vec2Read outperformed all other models, whereas the 3 PLMs shared a level of performance similar to engineered features. ChatGPT was not scalable to this task. On the generalization task, ChatGPT outperformed both the PLMs and Vec2Read. The level of ChatGPT was on par with the best-performing embedding-based models, even though ChatGPT was not specifically trained or tuned toward this task. These baseline models set an exceedingly high target for the text classification task but also confirm the difficulty of the generalization task.

Model depth and parameters

A comprehensive analysis of the influence of model depth and parameters on both tasks is presented in Figure 3, complemented by a statistical examination provided in Table 2.

Overall, the figure reveals a positive correlation between the number of parameters and performance in the text classification task, with the most significant impact observed for the PDfeat feature set. For the generalization task, parameter count contributes substantially to generalization performance for the PDfeat-GLM and PDfeat-FNN combinations. In contrast, BERT-based models exhibit either no correlation or a negative relationship between parameter count and generalization performance. In fact, parameter count has a small, negative, but significant effect on generalization performance for BERT-GLM, as shown in Table 2. These findings suggest that while more complex models enhance classification performance, they may impede generalization to the generalization task, particularly for BERT-based configurations.

Model depth generally shows a positive influence on training and generalization performance for most configurations in Table 2, indicating that greater depth enables models to better capture and generalize textual complexity. However, exceptions emerged with BERT features: GLM-depth negatively impacted test performance, while FNN-depth failed to enhance generalization performance. For GLM configurations (PDfeat-GLM and

Table 2. Regression models that examine the influence of the number of parameters, the model depth, and their interaction on the performance of textual complexity models

	Parameters		Depth		Interaction		Fit	
Model	β	t	β	t	β	t	F	R^2
Test performance								
SB23 and FNN	1.71	5.04***	0.97	7.84***	−1.48	−4.03***	40.71***	0.61
BERT and FNN	2.31	5.52***	0.32	2.08*	−2.05	−4.50***	17.34***	0.40
BERT and GLM	1.17	11.95***	−0.63	−16.01***	−1.06	−10.04***	655.60***	0.97
SB23 and GLM	18.99	22.75***	1.03	10.73***	−19.65	−22.17***	184.61***	0.91
Target performance								
SB23 and FNN	0.61	7.51***	0.25	3.03**	–	–	37.46***	0.48
BERT and FNN	0.08	0.69	−0.14	−1.23	–	–	0.88	0.02
BERT and GLM	−1.24	−4.06***	0.44	3.59***	1.20	3.64***	49.86***	0.72
SB23 and GLM	8.42	7.98***	0.78	6.45***	−8.24	−7.36***	108.78***	0.86

Possible colinearity of the interaction term was addressed via backward stepwise selection, with missing values indicating its removal. The degrees of freedom for the t and F tests are $t(57)$ and $F(3,57)$ for the GLM regression models and $t(79)$ and $F(3,79)$ for the FNN regression models. Significance levels: ***p < .001, * p < .01, *p < .05.

BERT-GLM), peak test performance consistently occurred at depth level 2 (Figures 3E and 3G), while peak generalization performance shifted to level 3 (Figures 3A and 3C). This suggests that 3-level multiplicative interactions in GLMs may obscure simpler patterns needed for classification, ultimately benefiting generalization. Conversely, for BERT-FNN configurations, peak performance for both tasks was observed at depth level 2. Additional depth likely focused on corpus-specific patterns, explaining why FNN-depth failed to enhance generalization performance with BERT features. These findings highlight that depth configurations optimizing test performance may not always translate to improved generalization and can sometimes hinder it. The overall effects of depth are modulated by interactions with other factors, particularly parameter count as a covariate to depth level and interaction type (i.e., linearity).

Feature contributions

Figure 4 provides an overview of the PDfeat features and their test and target correlations. Most features align with expectations: word length $(\text{Len}^{\text{cha}}, \text{Len}^{\text{syl}})$, semantic entropy (Sem_n), and dependency length (Dep) correlate positively with complexity, while connectives $(\text{Con}^{\text{cau}}, \text{Con}^{\text{alt}})$ and word frequency (LogPr) tend to simplify texts. Furthermore, parameterized features exhibit both positive and negative relations with complexity due to their additive effects. For instance, semantic and referential cohesion over 3–4 preceding sentences positively contribute to complexity, while cohesion over 1–2 preceding sentences has a negative impact. This implies that simpler texts employ fewer references over longer distances and more references over shorter distances. These findings indicate that features conform to expectations and are accurately derived from the training data.

Comparing the behavior of features between training and target data in Figure 4 reveals some discrepancies. On the target data, referential and semantic cohesion are associated with more complex texts, while lexical entropy is linked to simpler texts, opposing the test data and initial expectations. These counterintuitive findings emphasize the unique characteristics of the target data drawn from a distinctive corpus of well-edited and refined news articles. This small, carefully selected set of articles presents its own specifics, which may not align with other corpora. While certain patterns may appear when features are considered individually in such a sample, a more accurate interpretation likely emerges when these features are viewed in conjunction with others. This underscores the complexity of accurately modeling textual nuances across diverse datasets, where the specifics of the target corpus amplify the generalization requirements posed by the generalization task.

Table 3 compares a selection of feature interactions along with their test and generalization performance. Compared to first-order features (Figure 4), second-order and third-order interactions exhibit a higher correspondence between test and target metrics. Conversely, model coefficients β for these interactions differ from both test and target metrics. This discrepancy reflects the additive effects resulting from numerous interactions in the model, complicating the direct interpretation of model coefficients for higher-order interactions. The increased alignment between test and generalization performance with multiplicative interactions offers an explanation for the heightened generalization performance observed with higher model depths (see Figure 2). Most top-10 interactions span across different features rather than feature parameters, suggesting that textual complexity arises from a simultaneous combination of factors across various semantic, lexical, and syntactic aspects.

DISCUSSION

We compared old-world and new-world statistics: 65,640 opaque FNNs and 12,000 interpretable GLM configurations were tuned, resulting in 166 FNNs and 120 GLMs, while comparing them with 5 baseline models: ChatGPT, 3 PLMs, and Vec2Read. This allowed us to evaluate the trade-off between accuracy and interpretability and its influence on the models' OoD generalization performance. Textual complexity classification was chosen as a task because it exemplifies this trade-off, favoring LLMs over interpretable ones to mirror the

CellPress
OPEN ACCESS

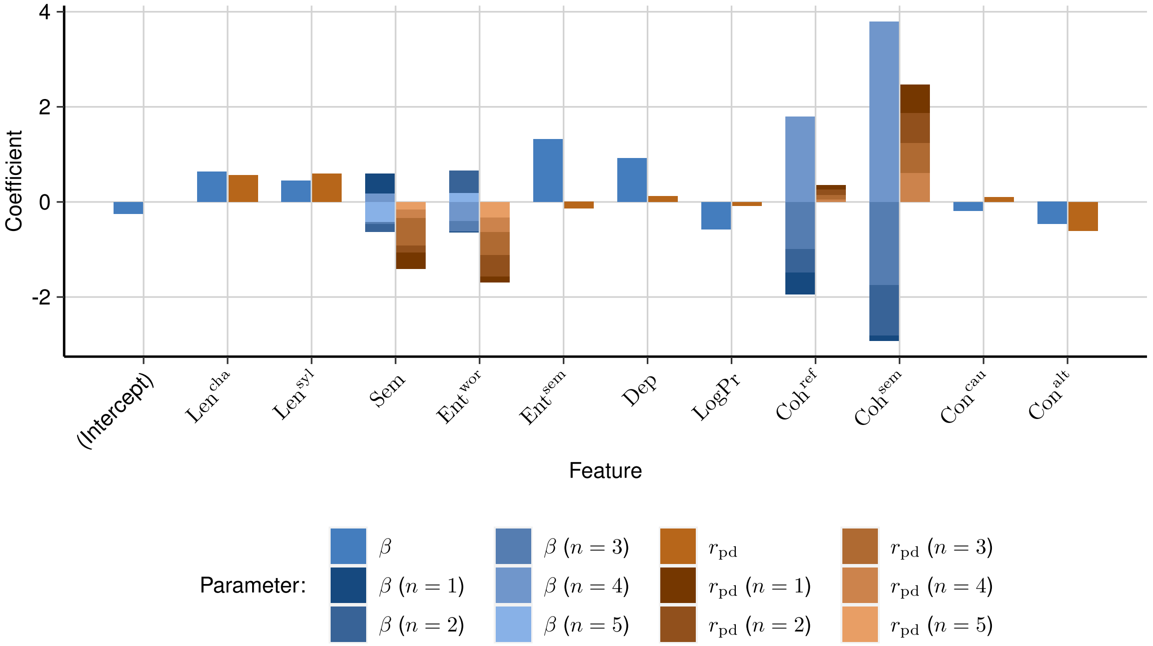

Figure 4. Feature importance as indicated by stacked coefficients for both training and target data

Parameterized features, such as cohesion over $n = 1\ldots4$ foregoing sentences, are grouped and stacked. Coefficients are either standardized regression coefficients (β) derived from the final GLM model with feature depth level 1 or Pearson' correlation with processing difficulty (r_{pd}). Note that parameterized feature regression coefficients are likely inflated inflated due to complementary yet opposing influences in the model. Negative values are indicative of simpler texts and positive values of more complex texts.

intricacies of human language processing. The predictive validity of 291 textual complexity models was assessed. Interpretability was enabled through a foundation in concepts and findings on human processing difficulty. Models' complexity was systematically varied using linear and nonlinear classification "heads," ranging across three depth levels and between 26 and 153,012 parameters. This array of modeling approaches and distinctive tasks created a comprehensive and challenging benchmark for evaluating the accuracy-interpretability trade-off.

Contrary to prevailing assumptions that interpretable models inherently sacrifice performance,[15] our results show the highest generalization performance with interpretable features and transparent model architectures. This surprising level of performance extends beyond previous studies with tabular data, where well-tuned interpretable models perform on par with opaque models. This difference is likely due to the increased demand for generalization: unlike tabular datasets that may cover various sampling sites and time frames, our setup required models to generalize across distinct genres and topics as well as tasks, measurements, and assessors, introducing both covariate and label shifts. Moreover, the observed limited generalizability of opaque models contrasts with recent abilities of LLMs to generalize across a variety of tasks, as also exemplified by the best-performing baseline of ChatGPT at $r = 0.421$. These deep, opaque models derive this ability from vast corpora of human-written texts, providing near-exhaustive training data. In contrast, our models, working with more limited data, demonstrate that interpretability enables generalizability in data-scarce environments, notably surpassing the best-performing opaque baseline (ChatGPT). This suggests a reevaluation of the accuracy-interpretability trade-off, especially for tasks requiring extensive generalization across full data shifts.

The addition of feature interactions significantly enhanced generalization performance for both linear and nonlinear interactions with engineered features. This reveals that textual complexity does not solely stem from individual features but rather emerges from their interplay. This interpretation is reinforced by the importance of cross-feature interactions, as evidenced in Table 3, and aligns with the recognized intricacies of human language processing and comprehension.[49] Linear, multiplicative interactions were particularly beneficial to generalization performance, outperforming nonlinear interactions. This benefit partially extended to BERT features, where additional linear feature depth improved generalization performance, unlike nonlinear interactions. This observed benefit can be attributed to the linear assumption. Introducing multiplicative interactions among up to three features ensures that all three features must co-occur simultaneously to influence predictions. This mechanism effectively shields the model from being influenced by irrelevant fluctuations in individual features. Furthermore, additional multiplicative interaction terms yielded surprisingly consistent incremental improvements in both classification and generalization performance (Figures 3A and 3E), similar to nonlinear interactions (Figures 3B and 3F). Accordingly, multiplicative interactions provide a gradual enhancement of models' capacity to capture complex causal structures while still being principally interpretable and maintaining robustness against corpus-specific details. This effectively enhances approximation power while controlling model bias and variance error, positioning multiplicative terms as a straightforward yet powerful tool to bolster

Table 3. Top 10 interactions between engineered features, ranked by regression coefficient β

Features	Test r_{pb}	Target r	Model β
Second-order interactions			
Sem_1, Sem_2	−0.07	−0.29	0.47
Len^{cha}, Len^{syl}	0.43	0.58	0.46
Sem_4, Sem_5	−0.16	−0.18	0.39
Coh_4^{sem}, Len^{syl}	0.22	0.63	−0.33
Coh_3^{ref}, Coh_4^{ref}	−0.10	0.04	0.30
Coh_1^{sem}, Len^{cha}	0.09	0.63	0.29
Len^{cha}, Sem_4	0.27	0.35	−0.25
Coh_3^{sem}, Coh_4^{sem}	0.05	0.60	0.25
Coh_4^{ref}, Ent_1^{wor}	−0.05	0.05	−0.23
Coh_4^{ref}, Sem_5	−0.07	0.04	−0.23
Third-order interactions			
Coh_4^{ref}, Ent_1^{wor}, Sem_1	−0.04	−0.03	−0.06
Ent^{sem}, Len^{cha}, Len^{syl}	0.45	0.56	0.06
LogPr, Coh_4^{ref}, Sem_2	0.01	0.03	0.04
LogPr, Coh_1^{sem}, Sem_2	−0.06	−0.70	−0.04
Coh_1^{ref}, Coh_2^{ref}, Ent^{sem}	−0.16	0.12	0.04
Ent^{sem}, Sem_4, Sem_5	−0.12	−0.19	0.04
Sem_1, Sem_4, Sem_5	−0.13	−0.29	0.04
Ent^{sem}, Sem_3, Sem_4	−0.14	−0.41	0.03
Con^{alt}, Ent_1^{wor}, Sem_2	−0.11	−0.58	0.03
Coh_3^{ref}, Coh_4^{ref}, Sem_2	−0.11	0.04	0.03

Regression coefficients are derived from models with second-order interactions and third-order interactions with the highest number of parameters. Test performance is indicated by interactions' point-biserial correlation (r_{pb}), and target performance is represented by Pearson's correlation (r). Regression coefficients are generally lower for higher-order interactions due to the multiplicative nature of resulting features.

the generalizability of interpretable models, especially for complex phenomena.

Our analysis reveals a distinctive trade-off in model performance between classification and generalization tasks, where configurations excelling in one often underperform in the other. For instance, the inclusion of nonlinear interactions benefited classification performance but either did not improve or even reduced generalization performance (Figure 2). This suggests that the added complexity from nonlinear interactions, though advantageous for classification, may learn task-specific details that hinder generalization to human judgments. This trade-off underscores the importance of modeling constraints that lead models to slightly underperform or underfit in the classification task. The engineered feature set and the assumption of linear interactions serve as such constraints. By identifying relevant features and constraining the search space, these mechanisms mitigate genre- and corpus-specific biases while guarding against shortcut learning from irrelevant patterns in the training data. Such constraints embody a form of external theory-ladenness that shapes the learning process, but they do not necessitate an internal theory of the relationships that produce a phenomenon through causal mechanisms. Whether rooted in psycholinguistics for textual complexity, in physics for physics-informed models, or in medical practice for physician-build machine learning models,[42–44] this type of theory specifies how models navigate the trade-off between complexity and interpretability, ensuring a balance between task-specific performance and overall generalizability.

Our methodology highlights the value of small-scale generalization-evaluation data. For training data, their size is principally important, as it allows for the mitigation of potential spurious and irrelevant relationships that would otherwise take precedence in the data.[21] Conversely, for target data, size is not a similar requirement, as uncommon relationships can benefit the target task. Our results demonstrate that such corpus specifics in target data rather amplify the generalization requirements posed by the target task. They increasingly necessitate that models capture generic principles that also perform well on specifics from the target data. Crucially, this presupposes the validity of these generalization specifics. This validity can be, as in our study, experimentally obtained in highly controlled settings using triangulated measures, capturing a specific set of configurations and nuances not seen in the training data. Moreover, this can involve the observation of extreme and relatively uncommon yet important cases, such as in fault modeling or adversarial learning.[41,77] Achieving this level of control and incorporating adversarial cases is more easily achieved at a small scale than the large scale needed for training. While the resulting datasets might be deemed too small for the specialization achieved through fine-tuning or re-training a model, they reinforce the imperative for a generic model to excel across diverse scenarios.

Limitations of the study

The question remains whether another, less-interpretable model could challenge the presented findings. With a maximum prediction performance of $r = 0.484$ for BERT, our results also underscore the potential of opaque models. Nevertheless, it is unlikely that newer embedding models will offer substantial improvements. BERT has shown competitive performance in classifying texts at different complexity levels when compared with other LLMs in cross-corpus studies,[60,78,79] even when included in interpretable architectures.[32] Additionally, our methodology revealed considerable variability in predictions by ChatGPT (see methods, baseline III: ChatGPT), underscoring the inherently stochastic nature of generative LLMs.[80] Another question remains about whether or not alternative training procedures could challenge the presented findings. Care was taken to prevent overfitting, particularly for FNNs, by including an early stopping mechanism. Furthermore, the training procedure incorporated specific constraints, such as a minimal weight decay of $1e-4$ for regularization, aimed at enhancing the models' generalizability. Nevertheless, this underscores that the results should be interpreted along with the training procedures involved.

While this study provides valuable insights into the accuracy-interpretability trade-off and generalization performance for textual complexity modeling, its findings are inherently tied to the specific case studied. The results are bounded by the models, data, and training procedures employed, including the focus on textual data and specific linear (GLM and PLM) and nonlinear (FNN, Vec2Read, and ChatGPT) model types. Notably, a key characteristic of the case is the lack of near-exhaustive training

CellPress
OPEN ACCESS

data, contrasting LLMs. This characteristic is common to real-world applications, where labeling costs are high, observational data are noisy, and scaling is not feasible. In such scenarios, the demonstrated benefit of interpretable models may extend to similar modeling challenges with restricted training data. Another key characteristic is the data shifts encountered: predicting human judgments from binary class labels (label shift) and generalizing across genres, topics, and text lengths (covariate shift). While these shifts reflect common challenges in modeling human judgments and behavior and a degree of subjectivity is inherent even in human-in-the-loop labeling processes, their specifics will vary across domains. These limitations leave open the question of whether and to what extent the findings presented here—on the accuracy-interpretability trade-off, generalization performance, and utility of multiplicative interactions—translate to other domains and tasks.

Conclusion

The rise of deep learning has revolutionized machine learning, driving breakthroughs across fields from text classification to image recognition and language modeling. By leveraging large datasets and raw inputs, data-intensive models have bypassed extensive feature engineering, making it possible to address previously intractable tasks. Yet, this shift has also amplified long-standing challenges, particularly around interpretability and generalization. Despite over 25 years of research, the interpretability-accuracy trade-off and generalization challenges remain central, underscoring their enduring relevance in machine learning.

This study offers a comprehensive comparison between interpretability and generalizability. By systematically varying interpretability and complexity, it evaluated models' ability to generalize. Our approach combines a large-scale training dataset with a smaller, controlled dataset, enabling us to assess generalization to human judgments. This setup introduces a full data shift: differences in measures, tasks, and raters lead to label shift, while domain differences in genre, topics, and text length contribute to covariate shift. Unlike transfer learning or fine-tuning approaches that rely on task-specific re-learning, this design necessitates generic models that can capture universal features and relationships, enabling effective generalization without re-training.

Our findings add to a limited yet expanding body of evidence on the comparative effectiveness of human-interpretable models against opaque ones.[15] Interpretability in this study was achieved by incorporating external theoretical insights on human processing difficulty into the feature set, combined with a generalized linear model. While these interpretable models showed lower test accuracy than their complex counterparts, they significantly outperformed them in generalizing to human judgments. Interestingly, incrementally adding linear, multiplicative interactions consistently improved generalization performance while maintaining principal interpretability. This enhancement may explain the discrepancy with earlier comparisons,[45] as multiplicative interactions enhance interpretable models' approximation power while maintaining robustness. These results suggest that while the accuracy-interpretability trade-off may apply to test accuracy, it does not necessarily extend to generalization, underscoring that interpretability and generalizability can instead complement each other.

METHODS

The experimental procedure followed a $2\times2+3$ setup:

- 2 distinct feature sets (embedding and engineered) × 2 classification heads (linear and nonlinear) +
- 3 baseline models ("rudimentary"; see Table 1 and Figure 1).

The evaluation encompassed the following:

(1) A train-test performance assessment with dataset I, which served as an objective ground truth on which to learn and compare different models.
(2) A generalization evaluation with dataset II, which allowed us to correlate the model's output with subjective ratings of processing difficulty. This evaluation was the primary objective of this study, aiming at validating the inferred models on their ability to transfer to a distinctive genre and increasingly subjective appraisals.

Within each experimental condition (see Table 1), diverse model variations were trained, spanning a range in model depth and model parameter count. This allowed an in-depth exploration of the models' behavior across both various dimensions of model complexity and two distinctive tasks.

Dataset I: EnSiWiki2020

The model of textual complexity was estimated from pairs of English Wikipedia and Simple English Wikipedia articles (see supplemental note C). Both Wikipedia versions have clear guidelines and review processes. This dataset was constructed utilizing the oldest articles on Wikipedia, ensuring the selection of mature pairs, which are more likely to conform to their intended levels of quality and complexity, making this dataset ideal as an objective ground truth for modeling textual complexity in combination with the large selection of matured articles.

Dataset II: Processing fluency

Participants' interest was evaluated using data from a self-paced reading study. Before reading, participants rated their topical familiarity for up to 5 topics associated with each article. Articles were subsequently grouped into 3 counterbalanced blocks based on topical familiarity and randomized within each block. This grouping served to control for known effects of familiarity on interest. Following theorizing on interest as governing consumptive behavior (termed "liking"), whether the consumption is and will remain rewarding, rather than approach behavior (termed "wanting"),[81] the articles were truncated at 1,200 characters, The study design provided a controlled setting with triangulated measurements, ensuring the reliability and validity of our data for assessing the extent to which textual complexity extends to human interest.

Feature set I: Psycholinguistic effects

The first feature set highlights core effects on processing difficulty, grounded in psycholinguistic findings. Key effects and their definitions are reviewed below, with supporting paradigms detailed in supplemental note D.

Word length

Longer words require additional time for perceptual recognition,[53] leading to extended fixation durations when reading[82] with concurrent effects on naming tasks[51] and lexical decision tasks.[52] Word length is formalized in two indexes of length N, character count and a phonetically relevant syllable count:

$$\begin{aligned} \mathrm{I}^a &= \|w\|, \text{with } w = x_1x_2\ldots x_N \in \Sigma, \\ \mathrm{I}^b &= \|w\|, \text{with } w = x_1x_2\ldots x_N \in S, \end{aligned} \qquad \text{(Equation 1)}$$

with w denoting a word.

Semantic neighborhood density

Whereas dense distant neighborhoods facilitate word recognition, dense near neighborhoods can impede it.[83] We formalize the neighborhood of a word based on the node degree A of a word in a semantic lexicon W,[84] defined as the number of nodes reached within $n = 1\ldots5$ steps of a word x:

$$\begin{aligned} \mathrm{II}_n &= \log|A_n(x)|, \quad \text{with} \\ A_n(x) &= A_{n-1}(x) \cup \{\phi \in W | r(\phi, \phi'), \phi' \in A_{n-1}(x)\} \quad \text{for } n \geq 1 \\ A_0(x) &= \{\phi \in W | x \in \phi\}, \end{aligned} \qquad \text{(Equation 2)}$$

where ϕ denotes a set of synonyms and $r(\phi, \phi')$ is a Boolean function indicating whether there is any relationship between synset ϕ and synset ϕ'. A logarithm applied to $A_n(x)$ and the Hirst-St Onge method (see supplemental note E) for $r(\phi, \phi')$ restrain the exponential growth of related synsets.

Word entropy

Within a meaningful context, repeated words or word sequences reduce first fixation and gaze duration, reflecting faster word recognition.[85] Entropy gives an inverse measure of word repetition. Given a text X with words $x_1x_2\ldots x_N$ from vocabulary V, we define the conditional entropy for sequences (n-grams) of length $n = 1\ldots4$ defined within a sliding window of length m applied over X:

$$\begin{aligned} \mathrm{III}_n^a &= \frac{1}{N-m}\sum_{i=m}^{N} H_n(x_{i-m+1}\ldots x_i) \text{ with } x \in V, \text{with} \\ \mathrm{H}_n(X) &= -\sum_{j=n}^{N} p(x_{j-(n-1)}\ldots x_j) \log_2 p(x_j | x_{j-(n-1)}\ldots x_{j-1}). \end{aligned} \qquad \text{(Equation 3)}$$

Semantic entropy

Words are better recognized when embedded in a semantically related local context.[85] We calculate the entropy of topic vectors $\vec{\tau}$ as a means of quantifying semantic context within a sliding window over the terms $X = x_1, x_2, \ldots, x_N \in V$:

$$\begin{aligned} \mathrm{III}^b &= \frac{1}{N-m}\sum_{i=m}^{N} H\left(\sum_{x_{i-m+1}}^{x_i} \vec{\tau}_x\right) \quad \text{with} \quad x_1x_2\ldots x_N \in V, \\ H(\vec{\tau}) &= -\sum_{t \in \vec{\tau}} p(t)\log_2 p(t) \quad \text{with} \quad p(t) = \frac{t}{|\vec{\tau}|_1}, \text{and} \\ \vec{\tau}_x &= [t_{x1}t_{x2}\ldots t_{xn}], \end{aligned} \qquad \text{(Equation 4)}$$

where n denotes the number of topic dimensions, t_{xd} the topic weight for term x on dimension d, and $p(t)$ is the probability of topic $t \in \vec{\tau}$.

Dependency length

Within a sentence, words are connected to each other by syntactical links. The length of these links is a primary determinant of the speed and accuracy of their resolution.[86] Distance effects can be observed through both a slowdown and an increase in regressive saccades at the region of dependency resolution.[87] Given a sentence with a set of dependencies $d \in D$ each spanning a sequence of words $d = x_1x_2\ldots x_N \in V$, the mean dependency length is given by

$$\mathrm{IV} = \frac{1}{|D_s|}\sum_{d \in D}\log_{10}\|x_2\ldots x_N \in d\| \qquad \text{(Equation 5)}$$

A log transformation is included as a standard way to normalize count data and make it suitable for a linear classifier.

Word probability

The more unlikely a (subsequent) word, the higher its surprisal, and the higher the processing effort it incurs. Word predictability affects online processing, most notably self-paced reading time, first-pass gaze duration, and pupil size,[88] and depends on its n preceding words:

$$\mathrm{V}_n = \frac{1}{N}\log\prod_{i=1}^{N} p(x_i | x_{i-n}\ldots x_{i-1}) \text{ with } x_1x_2\ldots x_N \in V, \qquad \text{(Equation 6)}$$

which gives the geometric mean of log probabilities of n-grams, following conventions,[88] at the sentence level.

Cohesion

Cohesive texts are characterized by expressions being "about the same thing." Except for highly knowledgeable readers, cohesion heightens comprehension and reduces reading time.[89] We specify a lexical and semantic index of local cohesion over $n = 1\ldots4$ foregoing sentences in a text of $s_1\ldots s_N$ sentences:

$$\begin{aligned} \mathrm{VI}_n^{a.b} &= \frac{1}{N-1}\sum_{i}^{N}\sum_{j}^{n} \mathrm{sim}^{a.b}(s_i, s_{i-j}) \quad \text{for } i - j \geq 1, \\ \mathrm{sim}^a(s_1, s_2) &= |\{r \in R | r \in s_1 \wedge r \in s_2\}| \quad \text{or} \\ \mathrm{sim}^b(s_1, s_2) &= \frac{\vec{\tau}(s_1)\cdot\vec{\tau}(s_2)}{\|\vec{\tau}(s_1)\|\|\vec{\tau}(s_2)\|}, \text{with} \\ \vec{\tau}(s) &= \frac{1}{\|s\|}\sum_{x \in s}\vec{\tau}_x, \end{aligned} \qquad \text{(Equation 7)}$$

where sim^a defines referential relatedness as the existence of a shared referent $r \in R$ between sentences and Sim^b defines semantic relatedness as the cosine similarity between sentence topic vectors $\vec{\tau}(s)$ (see also Equation 4).

Connectives

Connectives, such as "moreover," "after," and "because," are linguistic markers that indicate discourse relations. The presence of connectives typically leads to faster processing

times for information following a coherence marker[90] and reduces re-reading times,[91] especially for difficult texts.[92] Let A be terms identified as connectives, either causal A^c or noncausal A^n; we specify the frequency of connectives in a text $X = x_1x_2\ldots x_N \in V$:

$$\mathrm{VII}^a = \frac{\|x_1x_2\ldots x_N | x \in A^c\|}{N} \text{ with } x_1x_2\ldots x_N \in V \text{ and}$$
$$\mathrm{VII}^b = \frac{\|x_1x_2\ldots x_N | x \in A^n\|}{N} \text{ with } x_1x_2\ldots x_N \in V. \quad \text{(Equation 8)}$$

The high linguistic variety in connective markers necessitates sophisticated NLP solutions for their detection, which we discuss in supplemental note F.

Feature set II: Embeddings

The second feature set encompasses word embeddings, a powerful technique in NLP. Word embeddings learn a representation of words through the resolution of surrogate tasks. This task is typically a variant on a Cloze test, where the model learns to fill in missing words in a given window of surrounding words. This seemingly unrelated task prompts the model to discern and encode semantic and linguistic relationships between words. Consequently, words that share similar contexts tend to be positioned closer to each other in the embedding space, reflecting their linguistic and semantic affinity.

Their ability to learn intricate structures of language makes word embeddings ideal for readability modeling. Notably, the BERT contextual embedding model has emerged as one of the most effective approaches for modeling textual complexity.[60,78,79] BERT consists of, among others, 12 embedding layers offering complementary representations of a word. Lower layers are primarily associated with surface-level features such as sentence length, while intermediate to high layers encode a rich hierarchy of syntactic and semantic information.[69] Upper layers, in particular, perform well on textual complexity classification tasks,[69] supporting their value as text representation for complexity modeling.

Based on these insights, we establish the subsequent feature set, encompassing all dimensions $d = 1\ldots768$ within the BERT model:

$$\mathrm{emb}^d = \text{the extent of a word } x\text{'s influence on dimension } d \text{ within the } 11^{\text{th}} \text{ layer of BERT.} \quad \text{(Equation 9)}$$

The feature set is computed by averaging word embeddings across words within a given text. This customary method of aggregation is enabled by the vector space properties of embedding spaces, which permit algebraic manipulation. Furthermore, a sliding window is applied to circumvent the input window size restrictions of 512 tokens (see supplemental note G).

Model I: Generalized linear model

The generalized linear model (GLM) assumes a linear association between the response variable and the predictor variables, allowing for the estimation of coefficients that quantify these relationships. This facilitates the interpretation of how changes in predictor variables influence the response. Second- and third-order interactions between input features were included in the model to vary model depth, while the number of terms was restricted to control model complexity.

To yield an unbiased estimation of classification performance on dataset I, a 5-fold cross-validation loop was executed. After performance evaluation, the model was re-trained on the full dataset for subsequent generalization evaluation on dataset II. Within each training iteration, the following processing pipeline was conducted:

(1) Data preprocessing: missing values were removed, and minor class imbalances were rectified to ensure equiprobable classes.
(2) Feature scaling: normalization via root-mean-square deviation $(\mathrm{RMSD})/\bar{x}$, as is common with GLMs.
(3) Feature set expansion: by the inclusion of first- to d^{th}-order interaction terms.
(4) Feature selection: from the expanded feature set, the top p terms were chosen after F-score ranking.
(5) Correlation-based pruning: to counter colinearity issues, features exhibiting correlation coefficients above $r > 0.9$ were eliminated.
(6) Classification: a GlmNET model with Lasso regularization was trained on the pruned feature set.[93] Lasso regularization is ideal for extensive feature spaces, as it regularizes model terms toward zero, effectively eliminating irrelevant features. Furthermore, a logistic or log-odds transformation was included for binary classification problems.

The cross-validation loop was run for depth levels $d = 1$, 2, and 3. The inclusion of interaction terms led to an expansion of the feature space, culminating in a peak of $7.52e - 7$ third-order terms for feature set II. To address the ensuing computational challenges and mitigate overfitting risks (see supplemental note H), the top p terms were selected through ranking based on the F score. The F test assesses the discriminatory power of individual features in distinguishing between classes. Its univariate nature and linear time complexity make it particularly well suited for handling large feature sets. For feature Set I, the total numbers of terms p were constrained to $50, 60, \ldots 350$ at depth level 2 and 100, 200, ..., 2,700 at depth level 3. For feature set II, p was constrained to 1,000, 2,000, ..., 30,000 at both depth levels. This methodological step ensures that the most informative interaction terms are incorporated, thereby likely enhancing the effectiveness of the classification process. The combination of depth values d and term limits p resulted in a total of 120 unique configurations that were evaluated. Using the packages glmnet, mlr3, and caret, the processing pipeline was implemented in R.

Model II: FNN

FNN is a standard deep learning architecture where information flows in one direction: from the input layer, through the hidden layers, to the output layer. Operating through multiple layers of interconnected nodes, or neurons, the FNN integrates information from neurons in preceding layers through a nonlinear activation function, thereby allowing the network to capture complex interactions between input features.

Nested resampling was implemented to tune, train, and evaluate a variety of FNN configurations. The nested resampling

comprised (1) an inner loop, executed via 5-fold cross-validation, and (2) an outer loop, involving an 80% − 20% train-test split, which were applied for each model under evaluation as follows:

(1) Hyperparameter tuning via the inner loop, applied to the train split, namely the learning rate (lr_rate = 1e − 5…1e − 1), weight decay (weight_decay = 1e − 4…1e − 1), dropout rate (dropout = 0, 0.1, 0.2, 0.3, and 0.4), and activation function (tanh, ReLU, softmax, and sigmoid) (see supplemental note I).
(2) Performance evaluation on the test split.
(3) Generalization evaluation on the full dataset, resulting in the final model.

Each FNN was trained with a batch size of 128 using binary cross-entropy as loss function combined with the logits (sigmoid) function optimized for binary classification. The Adam optimizer, a robust learning algorithm, was coupled with an early stopping algorithm to proactively enhance model performance and generalization, guarding against overfitting (see supplemental note I). Furthermore, preprocessing addressed minor class imbalances and scaled the input features by their mean and standard deviation. This processing pipeline was constructed on pytorch in Python.

FNN training, following the nested resampling protocol, was applied across a range of architectures. The FNN architecture was varied on depth ($d = 1, 2, \text{and } 3$) and width ($n = 4, 8, \dots, 164$ for $d > 1$) of layers. Furthermore, at $d > 1$, dropout layers were included after each hidden layer. In total, 166 distinctive configurations were evaluated across both datasets, ensuring a thorough exploration of model behavior and performance under diverse architectures.

Baseline I: PLMs

In addition to the 2 feature sets and classification models, 3 baseline models were employed. These baseline techniques integrate features and classification heads into a single model. Baseline I employs PLMs to estimate the likelihood that a text adheres to the modeled language. This estimation relies on the trigram assumption through the use of n-grams, which simplifies estimations by assuming that the probability of a word depends on n preceding words. These models have been instrumental in readability modeling, where n-gram probabilities are learned from texts representing varying reading levels, effectively distinguishing among multi-class readability grades.[47,94] Unlike neural language models that utilize hidden layers and nonlinear activation functions, e.g., tanh,[95] given the additive nature of probabilities, n-gram PLMs can be viewed as linear models. PLMs nevertheless capture interactions between words in n-grams, which allows them to capture n^{th}-order multiplicative dependencies between words from language data.

PLMs were trained using 3-fold cross-validation to ensure unbiased performance estimates. Per iteration, two models were trained: one for English and one for simple English. A class prediction was based on the log likelihood ratio between the two models. After cross-validation, each model was re-trained on the full EnSiWiki2020 dataset. The regular English model was subsequently used for generalization evaluation through correlating the log probability for an observed text against subjective ratings, predicting the likelihood that a text was generated by the complex PLM. The use of logscale is in line with Smith and Levy,[88] who showed its superiority in predicting word-level reading time. PLMs were trained using the IRSTLM toolkit.[96] For all PLMs, the Witten-Belt smoothing method was applied. In total, 25 PLMs were evaluated with n-grams (1–5) and varying vocabulary sizes, replacing beyond-vocabulary words with POS tags for enhanced model generalization[68] (see supplemental note J).

Baseline II: Vec2Read

For baseline II, the state-of-the-art readability model Vec2Read was used.[58,97] Vec2Read employs rudimentary inputs with a deep learning architecture to estimate the readability of a given text. Its inputs are words, POS tags,[68] character-word skip-gram embeddings by FastText,[98] and a set of morphological tags extracted using SyntaxNet.[99] The model's architecture involves a multi-layer recurrent neural network, where each input is processed through a bidirectional long short-term memory (LSTM) layer. Its multi-layeredness and recurrence make this model able to not only generate feature representations from its inputs but also discern complex interactions both within and across its layers. For modeling, it uses a multi-layer recurrent neural network with bidirectional LTSM layers for each of its inputs and a single recurrence layer. Its ability to cover complex interactions allows for this model to be classified as "deep" (see Table 1).

Vec2Read was trained with a 0.01 learning rate and 100 epochs and tested on EnSiWiki2020. An 80% training-20% testing split was used. The resulting model attained an accuracy of 97.09%, a precision of 0.974, a recall of 0.967, and an F2 of 0.971. Given the resource-intensive nature of the training process, no variations were explored concerning the number of input features.

Baseline III: ChatGPT

For baseline III, ChatGPT 3.5 was employed. As an LLM, ChatGPT has demonstrated proficiency in generating texts at different reading levels. However, given the extent of training data, it was impractical to fine-tune ChatGPT or similar LLMs. Consequently, ChatGPT was solely tasked, without re-training, to predict the reading level associated with each target text.

The employed prompt is given in supplemental note K. Predictions were elicited for both a labeled and a numeric scale, with the numeric scale demonstrating superior efficacy. 3× per target text, the specified prompt was presented, and the resultant values were averaged across the 3 predictions. The averaging process exhibited enhanced predictive performance compared to any singular prediction.

RESOURCE AVAILABILITY

Lead contact

Any additional information required to reanalyze the data reported in this paper is available from the lead contact, Egon L. van den Broek (vandenbroek@acm.org), upon reasonable request.

Materials availability

This study did not generate new materials. Datasets used for model training and evaluation are taken from existing data sources: Wikipedia and van der Sluis and van den Broek.[67] These datasets are available on FigShare.[65,66]

Data and code availability

Our source code is publicly available in a GitHub repository at https://github.com/fsluis/textual-complexity and has been archived at Zenodo: https://doi.org/10.5281/zenodo.14359835.[100] This repository includes code for feature extraction and model training. Original data generated in this study, including intermediate and final results related to feature extraction and model training, have been deposited in FigShare: https://doi.org/10.6084/m9.figshare.25676394.[101]

ACKNOWLEDGMENTS

The Dutch Organization for Scientific Research (NWO) is gratefully acknowledged for funding the IPPSI-KIEM project Adaptive Text-Mining (ATM) (project no. 628.005.006). E.L.v.d.B. is also thankful to the UU-HONDA project, a cooperation between the Honda Research Institute in Japan and Utrecht University in the Netherlands.

AUTHOR CONTRIBUTIONS

Conceptualization, F.v.d.S. and E.L.v.d.B.; data curation, F.v.d.S.; formal analysis, F.v.d.S.; funding acquisition, F.v.d.S. and E.L.v.d.B.; investigation, F.v.d.S. and E.L.v.d.B.; methodology, F.v.d.S. and E.L.v.d.B.; project administration, F.v.d.S. and E.L.v.d.B.; resources, F.v.d.S. and E.L.v.d.B.; software, F.v.d.S.; validation, F.v.d.S. and E.L.v.d.B.; visualization, F.v.d.S.; writing – original draft, F.v.d.S.; writing – review & editing, F.v.d.S. and E.L.v.d.B.

DECLARATION OF INTERESTS

The authors declare that they have no competing interests.

DECLARATION OF GENERATIVE AI AND AI-ASSISTED TECHNOLOGIES IN THE WRITING PROCESS

The authors used ChatGPT-4o for language refinement and editing assistance during the preparation of this work. The content was subsequently reviewed and edited by the authors, who take full responsibility for its final version.

SUPPLEMENTAL INFORMATION

Supplemental information can be found online at https://doi.org/10.1016/j.patter.2025.101177.

Received: April 8, 2024
Revised: August 30, 2024
Accepted: January 14, 2025
Published: February 6, 2025

REFERENCES

1. LeCun, Y., Bengio, Y., and Hinton, G. (2015). Deep learning. Nature *521*, 436–444. https://doi.org/10.1038/nature14539.
2. Benjamin, R.G. (2012). Reconstructing readability: Recent developments and recommendations in the analysis of text difficulty. Educ. Psychol. Rev. *24*, 63–88. https://doi.org/10.1007/s10648-011-9181-8.
3. Ekbia, H., Mattioli, M., Kouper, I., Arave, G., Ghazinejad, A., Bowman, T., Suri, V.R., Tsou, A., Weingart, S., and Sugimoto, C.R. (2015). Big data, bigger dilemmas: A critical review. J. Assoc. Inf. Sci. Technol. *66*, 1523–1545. https://doi.org/10.1002/asi.23294.
4. Pietsch, W. (2016). The causal nature of modeling with big data. Philos. Technol. *29*, 137–171. https://doi.org/10.1007/s13347-015-0202-2.
5. Fan, J., Ma, C., and Zhong, Y. (2021). A selective overview of deep learning. Stat. Sci. *36*, 264–290. https://doi.org/10.1214/20-STS783.
6. Ananthaswamy, A. (2023). In AI, is bigger always better? Nature *615*, 202–205. https://doi.org/10.1038/d41586-023-00641-w.
7. Dunson, D.B. (2018). Statistics in the big data era: Failures of the machine. Stat. Probab. Lett. *136*, 4–9. https://doi.org/10.1016/j.spl.2018.02.028.
8. Miró-Nicolau, M., i Capó, A.J., and Moyà-Alcover, G. (2025). A comprehensive study on fidelity metrics for XAI. Inf. Process. Manag. *62*, 103900. https://doi.org/10.1016/j.ipm.2024.103900.
9. Wang, J., Lan, C., Liu, C., Ouyang, Y., Qin, T., Lu, W., Chen, Y., Zeng, W., and Yu, P. (2022). Generalizing to unseen domains: A survey on domain generalization. IEEE Trans. Knowl. Data Eng. *35*, 1–8072. https://doi.org/10.1109/TKDE.2022.3178128.
10. Zhou, K., Liu, Z., Qiao, Y., Xiang, T., and Loy, C.C. (2023). Domain generalization: A survey. IEEE Trans. Pattern Anal. Mach. Intell. *45*, 4396–4415. https://doi.org/10.1109/TPAMI.2022.3195549.
11. Teney, D., Lin, Y., Oh, S.J., and Abbasnejad, E. (2023). ID and OOD performance are sometimes inversely correlated on real-world datasets. In Proceedings of the 36th Conference on Neural Information Processing Systems (NeurIPS 2023) vol. 36 of *Advances in Neural Information Processing Systems*, A. Oh, T. Naumann, A. Globerson, K. Saenko, M. Hardt, and S. Levine, eds. (Curran Associates, Inc.), pp. 71703–71722.
12. Zhao, Z., and Cao, L. (2024). Weighting non-IID batches for out-of-distribution detection. Mach. Learn. *113*, 7371–7391. https://doi.org/10.1007/s10994-024-06605-z.
13. Graziani, M., Dutkiewicz, L., Calvaresi, D., Amorim, J.P., Yordanova, K., Vered, M., Nair, R., Abreu, P.H., Blanke, T., Pulignano, V., et al. (2023). A global taxonomy of interpretable AI: Unifying the terminology for the technical and social sciences. Artif. Intell. Rev. *56*, 3473–3504. https://doi.org/10.1007/s10462-022-10256-8.
14. van der Sluis, F., van den Broek, E.L., Glassey, R.J., van Dijk, E.M.A.G., and de Jong, F.M.G. (2014). When complexity becomes interesting. J. Assoc. Inf. Sci. Technol. *65*, 1478–1500. https://doi.org/10.1002/asi.23095.
15. Rudin, C. (2019). Stop explaining black box machine learning models for high stakes decisions and use interpretable models instead. Nat. Mach. Intell. *1*, 206–215. https://doi.org/10.1038/s42256-019-0048-x.
16. Rudin, C., Chen, C., Chen, Z., Huang, H., Semenova, L., and Zhong, C. (2022). Interpretable machine learning: Fundamental principles and 10 grand challenges. Stat. Surv. *16*, 1–85. https://doi.org/10.1214/21-\SS133.
17. Chancellor, S. (2023). Toward practices for human-centered machine learning. Commun. ACM *66*, 78–85. https://doi.org/10.1145/3530987.
18. Gillies, M., Lee, B., d'Alessandro, N., Tilmanne, J., Kulesza, T., Caramiaux, B., Fiebrink, R., Tanaka, A., Garcia, J., Bevilacqua, F., et al. (2016). Human-centred machine learning. In Proceedings of the 2016 CHI Conference Extended Abstracts on Human Factors in Computing Systems - CHI EA '16, C. Lampe, D. Morris, and J.P. Hourcade, eds. (ACM), pp. 3558–3565. https://doi.org/10.1145/2851581.2856492.
19. Aragon, C., Guha, S., Kogan, M., Muller, M., and Neff, G. (2022). Human-Centered Data Science: An Introduction (The MIT Press).
20. Kern, C., Gerdon, F., Bach, R.L., Keusch, F., and Kreuter, F. (2022). Humans versus machines: Who is perceived to decide fairer? Experimental evidence on attitudes toward automated decision-making. Patterns *3*, 100591. https://doi.org/10.1016/j.patter.2022.100591.
21. Desai, M.A., Pasquetto, I.V., Jacobs, A.Z., and Card, D. (2024). An archival perspective on pretraining data. Patterns *5*, 100966. https://doi.org/10.1016/j.patter.2024.100966.
22. Lipton, Z.C. (2018). The mythos of model interpretability. Queue *16*, 31–57. https://doi.org/10.1145/3236386.3241340.
23. Caliskan, A., Bryson, J.J., and Narayanan, A. (2017). Semantics derived automatically from language corpora contain human-like biases. Science *356*, 183–186. https://doi.org/10.1126/science.aal4230.
24. Breiman, L. (2001). Statistical modeling: The two cultures. Stat. Sci. *16*, 199–231. https://doi.org/10.1214/ss/1009213726.
25. Rudin, C., Zhong, C., Semenova, L., Seltzer, M., Parr, R., Liu, J., Katta, S., Donnelly, J., Chen, H., and Boner, Z. (2024). Amazing things come from

having many good models. Proceedings of Machine Learning Research *235*, 42783–42795.

26. Semenova, L., Rudin, C., and Parr, R. (2022). On the existence of simpler machine learning models. In Proceedings of the 5th ACM Conference on Fairness, Accountability, and Transparency (FAccT'22), C. Isbell, S. Lazar, A. Oh, A. Xiang, K. Lum, M. Kearns, A.X. Wu, and F.Z. Borgesius, eds. (ACM), pp. 1827–1858. https://doi.org/10.1145/3531146.3533232.
27. Razavian, N., Blecker, S., Schmidt, A.M., Smith-McLallen, A., Nigam, S., and Sontag, D. (2015). Population-level prediction of type 2 diabetes from claims data and analysis of risk factors. Big Data *3*, 277–287. https://doi.org/10.1089/big.2015.0020.
28. Tollenaar, N., and van der Heijden, P.G.M. (2012). Which method predicts recidivism best? a comparison of statistical, machine learning and data mining predictive models. J. R. Stat. Soc. Ser. A Stat. Soc. *176*, 565–584. https://doi.org/10.1111/j.1467-985X.2012.01056.x.
29. Zeng, J., Ustun, B., and Rudin, C. (2017). Interpretable classification models for recidivism prediction. J. R. Stat. Soc. Ser. A Stat. Soc. *180*, 689–722. https://doi.org/10.1111/rssa.12227.
30. Kung, C., and Yu, R. (2020). Interpretable models do not compromise accuracy or fairness in predicting college success. In Proceedings of the Seventh ACM Conference on Learning @ Scale (L@S), R. Kizilcec and S. Singer, eds. (ACM), pp. 413–416. https://doi.org/10.1145/3386527.3406755.
31. Yadav, R.K., Jiao, L., Granmo, O.-C., and Goodwin, M. (2021). Human-level interpretable learning for aspect-based sentiment analysis. Proc. AAAI Conf. Artif. Intell. *35*, 14203–14212. https://doi.org/10.1609/aaai.v35i16.17671.
32. Singh, C., Askari, A., Caruana, R., and Gao, J. (2023). Augmenting interpretable models with large language models during training. Nat. Commun. *14*, #7913. https://doi.org/10.1038/s41467-023-43713-1.
33. Zech, J.R., Badgeley, M.A., Liu, M., Costa, A.B., Titano, J.J., and Oermann, E.K. (2018). Variable generalization performance of a deep learning model to detect pneumonia in chest radiographs: A cross-sectional study. PLoS Med. *15*, e1002683. https://doi.org/10.1371/journal.pmed.1002683.
34. Lapuschkin, S., Wäldchen, S., Binder, A., Montavon, G., Samek, W., and Müller, K.-R. (2019). Unmasking Clever Hans predictors and assessing what machines really learn. Nat. Commun. *10*, 1096. https://doi.org/10.1038/s41467-019-08987-4.
35. Bavaresco, A., Bernardi, R., Bertolazzi, L., Elliott, D., Fernández, R., Gatt, A., Ghaleb, E., Giulianelli, M., Hanna, M., Koller, A., et al. (2024). LLMs instead of human judges? a large scale empirical study across 20 NLP evaluation tasks. Preprint at arXiv. https://doi.org/10.48550/arxiv.2406.18403.
36. Golan, T., Siegelman, M., Kriegeskorte, N., and Baldassano, C. (2023). Testing the limits of natural language models for predicting human language judgements. Nat. Mach. Intell. *5*, 952–964. https://doi.org/10.1038/s42256-023-00718-1.
37. Crowson, M.G., Bates, D.W., Suresh, K., Cohen, M.S., and Hartnick, C.J. (2023). Survey on explainable AI: From approaches, limitations and applications aspects. Hum.-Cent. Intell. Syst. *3*, 161–188. https://doi.org/10.1007/s44230-023-00038-y.
38. Crowson, M.G., Bates, D.W., Suresh, K., Cohen, M.S., and Hartnick, C.J. (2023). "Human vs Machine" validation of a deep learning algorithm for pediatric middle ear infection diagnosis. Otolaryngol. Head Neck Surg. *169*, 41–46. https://doi.org/10.1177/01945998221119156.
39. Burk-Rafel, J., Reinstein, I., Feng, J., Kim, M.B., Miller, L.H., Cocks, P.M., Marin, M., and Aphinyanaphongs, Y. (2021). Development and validation of a machine learning-based decision support tool for residency applicant screening and review. Acad. Med. *96*, S54–S61. https://doi.org/10.1097/ACM.0000000000004317.
40. Whang, S.E., Roh, Y., Song, H., and Lee, J.-G. (2023). Data collection and quality challenges in deep learning: A data-centric AI perspective. VLDB J. *32*, 791–813. https://doi.org/10.1007/s00778-022-00775-9.
41. Buckner, C. (2020). Understanding adversarial examples requires a theory of artefacts for deep learning. Nat. Mach. Intell. *2*, 731–736. https://doi.org/10.1038/s42256-020-00266-y.
42. Kadambi, A., de Melo, C., Hsieh, C.-J., Srivastava, M., and Soatto, S. (2023). Incorporating physics into data-driven computer vision. Nat. Mach. Intell. *5*, 572–580. https://doi.org/10.1038/s42256-023-00662-0.
43. Willard, J., Jia, X., Xu, S., Steinbach, M., and Kumar, V. (2023). Integrating scientific knowledge with machine learning for engineering and environmental systems. ACM Comput. Surv. *55*, 1–37. https://doi.org/10.1145/3514228.
44. Mekki, Y.M. (2024). Physicians should build their own machine-learning models. Patterns *5*, 100948. https://doi.org/10.1016/j.patter.2024.100948.
45. Crawford, J., Chikina, M., and Greene, C.S. (2024). Best holdout assessment is sufficient for cancer transcriptomic model selection. Patterns *5*, 101115. https://doi.org/10.1016/j.patter.2024.101115.
46. van der Sluis, F., Ginn, J., and van der Zee, T. (2016). Explaining student behavior at scale: the influence of video complexity on student dwelling time. In Proceedings of the Third (2016) ACM Conference on Learning @ Scale (L@S), V. Aleven, J. Kay, and I. Roll, eds. (ACM), pp. 51–60. https://doi.org/10.1145/2876034.2876051.
47. Collins-Thompson, K., and Callan, J. (2005). Predicting reading difficulty with statistical language models. J. Am. Soc. Inf. Sci. Technol. *56*, 1448–1462. https://doi.org/10.1002/asi.20243.
48. Van der Sluis, F., and Van den Broek, E.L. (2010). Using complexity measures in information retrieval. In ACM Proceedings of the 3th symposium on Information Interaction in Context (IIiX), N.J. Belkin and D. Kelly, eds. (ACM), pp. 383–386. https://doi.org/10.1145/1840784.184084.
49. LaBerge, D., and Samuels, S. (1974). Toward a theory of automatic information processing in reading. Cognit. Psychol. *6*, 293–323. https://doi.org/10.1016/0010-0285(74)90015-2.
50. Collins-Thompson, K. (2014). Computational assessment of text readability. ITL - Int. J. Appl. Linguist. *165*, 97–135. https://doi.org/10.1075/itl.165.2.01col.
51. Balota, D.A., Cortese, M.J., Sergent-Marshall, S.D., Spieler, D.H., and Yap, M. (2004). Visual word recognition of single-syllable words. J. Exp. Psychol. Gen. *133*, 283–316. https://doi.org/10.1037/0096-3445.133.2.283.
52. New, B., Ferrand, L., Pallier, C., and Brysbaert, M. (2006). Reexamining the word length effect in visual word recognition: New evidence from the English lexicon project. Psychon. Bull. Rev. *13*, 45–52. URL:. https://doi.org/10.3758/BF03193811
53. McGinnies, E., Comer, P., and Lacey, O. (1952). Visual-recognition thresholds as a function of word length and word frequency. J. Exp. Psychol. *44*, 65–69. https://doi.org/10.1037/h0063142.
54. Vajjala, S., and Meurers, D. (2014). Assessing the relative reading level of sentence pairs for text simplification. In Proceedings of the 14th Conference of the European Chapter of the Association for Computational Linguistics, S. Goldwater and S. Riezler, eds. (Association for Computational Linguistics), pp. 288–297. https://doi.org/10.3115/v1/E14-1031.
55. Chen, X., and Meurers, D. (2016). Characterizing text difficulty with word frequencies. In Proceedings of the 11th Workshop on Innovative Use of NLP for Building Educational Applications, J. Tetreault, J. Burstein, C. Leacock, and H. Yannakoudakis, eds. (Association for Computational Linguistics), pp. 84–94. https://doi.org/10.18653/v1/W16-0509.
56. Xia, M., Kochmar, E., and Briscoe, T. (2016). Text readability assessment for second language learners. In In: Tetreault J., Burstein J., Leacock C., Yannakoudakis H., editors. Proceedings of the 11th Workshop on Innovative Use of NLP for Building Educational Applications (Association for Computational Linguistics), pp. 12–22. https://doi.org/10.18653/v1/W16-0502.
57. Lee, J., and Vajjala, S. (2022). A neural pairwise ranking model for readability assessment. In Findings of the Association for Computational Linguistics: ACL 2022, S. Muresan, P. Nakov, and A. Villavicencio, eds.

CellPress
OPEN ACCESS

(Association for Computational Linguistics), pp. 3802–3813. https://doi.org/10.18653/v1/2022.findings-acl.300.

58. Madrazo Azpiazu, I., and Pera, M.S. (2020). Is cross-lingual readability assessment possible? J. Assoc. Inf. Sci. Technol. *71*, 644–656. https://doi.org/10.1002/asi.24293.
59. François, T. (2015). When readability meets computational linguistics: A new paradigm in readability. Rev. Fr. Ling. Appl. *20*, 79–97. https://doi.org/10.3917/rfla.202.0079.
60. Martinc, M., Pollak, S., and Robnik-Šikonja, M. (2021). Supervised and unsupervised neural approaches to text readability. Comput. Ling. *47*, 141–179. https://doi.org/10.1162/coli_a_00398.
61. van Oosten, P., Hoste, V., and Tanghe, D. (2011). A posteriori agreement as a quality measure for readability prediction systems. In Computational linguistics and intelligent text processing vol. 6609 of *Lecture notes in computer science*, A. Gelbukh, ed. (Springer Berlin Heidelberg), pp. 424–435. https://doi.org/10.1007/978-3-642-19437-5_35.
62. Crossley, S.A., Skalicky, S., Dascalu, M., McNamara, D.S., and Kyle, K. (2017). Predicting text comprehension, processing, and familiarity in adult readers: New approaches to readability formulas. Discourse Process *54*, 340–359. https://doi.org/10.1080/0163853X.2017.1296264.
63. van den Broek, P. (1995). A 'landscape' model of reading comprehension: Inferential processes and the construction of a stable memory representation. Can. Psychol./Psychol. Canad. *36*, 53–54. https://doi.org/10.1037/h0084723.
64. Hupkes, D., Giulianelli, M., Dankers, V., Artetxe, M., Elazar, Y., Pimentel, T., Christodoulopoulos, C., Lasri, K., Saphra, N., Sinclair, A., et al. (2023). A taxonomy and review of generalization research in NLP. Nat. Mach. Intell. *5*, 1161–1174. https://doi.org/10.1038/s42256-023-00729-y.
65. van der Sluis, F.,, van den Broek E.L. (2025). EnSiWiki-2020 corpus for textual complexity modelling. FigShare. https://doi.org/10.6084/m9.figshare.25676209.
66. van der Sluis, F., and van den Broek, E.L. (2025). The Guardian reading dataset. FigShare. https://doi.org/10.6084/m9.figshare.27057958.
67. van der Sluis, F., and van den Broek, E.L. (2023). Feedback beyond accuracy: Using eye-tracking to detect comprehensibility and interest during reading. J. Assoc. Inf. Sci. Technol. *74*, 3–16. https://doi.org/10.1002/asi.24657.
68. Kaptein, R., Koot, G., Veld, M.A.A.H.i.'., and van den Broek, E.L. (2014). Needle Custom Search: Recall-oriented search on the web using semantic annotations. In Advances in Information Retrieval: Proceedings of the 36th European Conference on IR Research, (ECIR 2014), *8416*, M. de Rijke, T. Kenter, A.P. de Vries, C. Zhai, F. de Jong, K. Radinsky, and K. Hofmann, eds. *(International Switzerland: Springer), pp. 750–753. https://doi.org/10.1007/978-3-319-06028-6_88*.
69. Jawahar, G., Sagot, B., and Seddah, D. (2019). What does BERT learn about the structure of language? In Proceedings of the 57th Annual Meeting of the Association for Computational Linguistics, A. Korhonen and D. Traum, eds. (Association for Computational Linguistics), pp. 3651–3657. https://doi.org/10.18653/v1/P19-1356.
70. Moreno-Torres, J.G., Raeder, T., Alaiz-Rodríguez, R., Chawla, N.V., and Herrera, F. (2012). A unifying view on dataset shift in classification. Pattern Recogn. *45*, 521–530. https://doi.org/10.1016/j.patcog.2011.06.019.
71. Devlin, J., Chang, M.-W., Lee, K., and Toutanova, K. (2019). BERT: pre-training of deep bidirectional transformers for language understanding. In Proceedings of the 2019 Conference of the North American Chapter of the Association for Computational Linguistics: Human Language Technologies, Volume 1 (Long and Short Papers), C. Doran and T. Solorio, eds. (Association for Computational Linguistics), pp. 4171–4186. https://doi.org/10.18653/v1/N19-1423.
72. Daumé, I.I.I.,H. (2007). Frustratingly easy domain adaptation. In Proceedings of the 45th Annual Meeting of the Association of Computational Linguistics, A. Zaenen and A. van den Bosch, eds. (Association for Computational Linguistics), pp. 256–263.
73. Poliak, A., Naradowsky, J., Haldar, A., Rudinger, R., and Van Durme, B. (2018). Hypothesis only baselines in natural language inference. In Proceedings of the Seventh Joint Conference on Lexical and Computational Semantics, J. Berant and A. Lenci, eds. (Association for Computational Linguistics), pp. 180–191. https://doi.org/10.18653/v1/S18-2023.
74. Crossley, S.A., Skalicky, S., and Dascalu, M. (2019). Moving beyond classic readability formulas: new methods and new models. J. Res. Read. *42*, 541–561. https://doi.org/10.1111/1467-9817.12283.
75. Crossley, S., Heintz, A., Choi, J.S., Batchelor, J., Karimi, M., and Malatinszky, A. (2023). A large-scaled corpus for assessing text readability. Behav. Res. Methods *55*, 491–507. https://doi.org/10.3758/s13428-022-01802-x.
76. Lim, H.H., Cai, T., Lee, J.S.Y., and Liu, M. (2022). Robustness of hybrid models in cross-domain readability assessment. In Proceedings of the The 20th Annual Workshop of the Australasian Language Technology Association. Australasian Language Technology Association, P. Parameswaran, J. Biggs, and D. Powers, eds., pp. 62–67.
77. Zhou, Z., and Firestone, C. (2019). Humans can decipher adversarial images. Nat. Commun. *10*, 1334. https://doi.org/10.1038/s41467-019-08931-6.
78. Lee, B.W., Jang, Y.S., and Lee, J. (2021). Pushing on text readability assessment: A transformer meets handcrafted linguistic features. In Proceedings of the 2021 Conference on Empirical Methods in Natural Language Processing, M.-F. Moens, X. Huang, L. Specia, and S.W.-t. Yih, eds. (Association for Computational Linguistics), pp. 10669–10686. https://doi.org/10.18653/v1/2021.emnlp-main.834.
79. Liu, F., and Lee, J. (2023). Hybrid models for sentence readability assessment. In Proceedings of the 18th Workshop on Innovative Use of NLP for Building Educational Applications (BEA 2023), E. Kochmar, J. Burstein, A. Horbach, R. Laarmann-Quante, N. Madnani, A. Tack, V. Yaneva, Z. Yuan, and T. Zesch, eds. (Association for Computational Linguistics), pp. 448–454. https://doi.org/10.18653/v1/2023.bea-1.37.
80. Gill, S.S., and Kaur, R. (2023). ChatGPT: Vision and challenges. Internet of Things and Cyber-Physical Systems *3*, 262–271. https://doi.org/10.1016/j.iotcps.2023.05.004.
81. Shin, D.D., and Kim, S.-i. (2019). Homo curious: Curious or interested? Educ. Psychol. Rev. *31*, 853–874. https://doi.org/10.1007/s10648-019-09497-x.
82. Just, M.A., and Carpenter, P.A. (1980). A theory of reading: From eye fixations to comprehension. Psychol. Rev. *87*, 329–354. https://doi.org/10.1037/0033-295X.87.4.329.
83. Mirman, D., and Magnuson, J.S. (2008). Attractor dynamics and semantic neighborhood density: processing is slowed by near neighbors and speeded by distant neighbors. J. Exp. Psychol. Learn. Mem. Cogn. *34*, 65–79. https://doi.org/10.1037/0278-7393.34.1.65.
84. Pexman, P.M., Hargreaves, I.S., Siakaluk, P.D., Bodner, G.E., and Pope, J. (2008). There are many ways to be rich: Effects of three measures of semantic richness on visual word recognition. Psychon. Bull. Rev. *15*, 161–167. https://doi.org/10.3758/pbr.15.1.161.
85. Ledoux, K., Camblin, C.C., Swaab, T.Y., and Gordon, P.C. (2006). Reading words in discourse: The modulation of lexical priming effects by message-level context. Behav. Cognit. Neurosci. Rev. *5*, 107–127. https://doi.org/10.1177/1534582306289573.
86. Gibson, E., Futrell, R., Piantadosi, S.P., Dautriche, I., Mahowald, K., Bergen, L., and Levy, R. (2019). How efficiency shapes human language. Trends Cognit. Sci. *23*, 389–407. https://doi.org/10.1016/j.tics.2019.02.003.
87. Demberg, V., and Keller, F. (2008). Data from eye-tracking corpora as evidence for theories of syntactic processing complexity. Cognition *109*, 193–210. https://doi.org/10.1016/j.cognition.2008.07.008.
88. Smith, N.J., and Levy, R. (2013). The effect of word predictability on reading time is logarithmic. Cognition *128*, 302–319. https://doi.org/10.1016/j.cognition.2013.02.013.

89. Ferstl, E.C. (2018). Text comprehension. In The Oxford handbook of psycholinguistics, S.-A. Rueschemeyer and M.G. Gaskell, eds. (Oxford University Press), pp. 196–216. https://doi.org/10.1093/oxfordhb/9780198786825.013.9.

90. Sanders, T.J.M., and Noordman, L.G.M. (2000). The role of coherence relations and their linguistic markers in text processing. Discourse Process *29*, 37–60. https://doi.org/10.1207/S15326950dp2901_3.

91. van Silfhout, G., Evers-Vermeul, J., and Sanders, T. (2015). Connectives as processing signals: how students benefit in processing narrative and expository texts. Discourse Process *52*, 47–76. https://doi.org/10.1080/0163853X.2014.905237.

92. Kleijn, S., Pander Maat, H.L., and Sanders, T.J. (2019). Comprehension effects of connectives across texts, readers, and coherence relations. Discourse Process *56*, 447–464. https://doi.org/10.1080/0163853X.2019.1605257.

93. Friedman, J., Hastie, T., and Tibshirani, R. (2010). Regularization paths for generalized linear models via coordinate descent. J. Stat. Software *33*, 1–22. https://doi.org/10.18637/jss.v033.i01.

94. Schwarm, S.E., and Ostendorf, M. (2005). Reading level assessment using support vector machines and statistical language models. In Proceedings of the 43rd Annual Meeting on Association for Computational Linguistics, H.T. Ng and K. Oflazer, eds. (Association for Computational Linguistics), pp. 523–530. https://doi.org/10.3115/1219840.1219905.

95. Bengio, Y., Ducharme, R., Vincent, P., and Janvin, C. (2003). A neural probabilistic language model. J. Mach. Learn. Res. *3*, 1137–1155.

96. Federico, M., Bertoldi, N., and Cettolo, M. (2008). IRSTLM: an open source toolkit for handling large scale language models. In Proceedings of the 9th Annual Conference of the International Speech Communication Association (Interspeech); International Speech Communication Association., D. Burnham, ed., pp. 1618–1621. https://doi.org/10.21437/Interspeech.2008-271.

97. Azpiazu, I.M., and Pera, M.S. (2019). Multiattentive recurrent neural network architecture for multilingual readability assessment. Trans. Assoc. Comput. Linguist. *7*, 421–436. https://doi.org/10.1162/tacl_a_00278.

98. Bojanowski, P., Grave, E., Joulin, A., and Mikolov, T. (2016). Enriching word vectors with subword information. Preprint at arXiv. https://doi.org/10.48550/arxiv.1607.04606.

99. Andor, D., Alberti, C., Weiss, D., Severyn, A., Presta, A., Ganchev, K., Petrov, S., and Collins, M. (2016). Globally normalized transition-based neural networks. In Proceedings of the 54th Annual Meeting of the Association for Computational Linguistics (Volume 1: Long Papers), K. Erk and N.A. Smith, eds. (Association for Computational Linguistics), pp. 2442–2452. https://doi.org/10.18653/v1/P16-1231.

100. van der Sluis, F. (2025). Code for the paper “Model interpretability enhances domain generalization in the case of textual complexity modeling”. Zenodo. (v1.0). https://doi.org/10.5281/zenodo.14359835.

101. van der Sluis, F., and van den Broek, E.L. (2025). Data for the paper “Model interpretability enhances domain generalization in the case of textual complexity modeling. FigShare. https://doi.org/10.6084/m9.figshare.25676394.

Patterns, Volume *6*

Supplemental information

Model interpretability enhances domain generalization in the case of textual complexity modeling

Frans van der Sluis and Egon L. van den Broek

SUPPLEMENTAL NOTES

Interpretability Benefits Generalizability: The Case of Modeling Textual Complexity

Frans van der Sluis and Egon L. van den Broek

PATTERNS
DECEMBER 28, 2024

This document includes supplemental notes, providing detailed insights, methods, and background information beyond the article's main text. The notes contain the following sections:

A AI READABILITY

B MODELS' LINEARITY

C ENSIWIKI2020 DETAILS

D EXPERIMENTAL PARADIGMS OF PROCESSING DIFFICULTY

E HIRST-STONGE NEIGHBORHOOD SIZE

F FEATURE IMPLEMENTATION DETAILS

G BERT STEPWISE TRAVERSAL

H FEATURE SPACE INFLATION

I HYPERPARAMETERS AND CONTROL MECHANISMS

J PROBABLISTIC LANGUAGE MODELS' HYPERPARAMETERS

K CHATGPT PROMPT

Followed by 56 references used in this supplemental notes.

A AI readability

Various tasks and datasets are employed in automatic readability detection. Typical tasks are text classification, rating prediction, and Cloze comprehension tests. other corpora, though not all available in the public domain. Notable data sources include the Common Core Appendix B (168 documents, grade levels 2 – 12) [S1], WeeBit (6 388 documents, 6 grade levels) [S2], CommonLit Ease of Readability (CLEAR) corpus (4 724 excerpts, linear ranking) [S3], and Wikipedia (up to 262 918 documents, binary levels) [S4, S5]. The diversity among these tasks and datasets poses a challenge in determining the overall state-of-the-art approaches. However, recent task- and data-specific performance may still be examined. Following a similar categorization as offered by Françis (2015) [S6] of “AI readability”, three approaches to readability detection will be reviewed: *i)* lexical-syntactic models; *ii)* structure-cognitivist models with either linguistically-motivated or cognitively-motivated features, and; *iii)* non-expert models.

Lexical-syntactic models capture word frequencies as well as syntactic features, such as through word or Part-Of-Speech (POS) n-grams or sentence parse tree height. In particular the mapping of word frequencies to grade levels necessitates the availability of a representative and sufficiently large training corpus. Statistical language models have proven successful in classifying texts of varying degrees of complexity. In Collins-Thompson and Callan [S7] performance ranged from 63% to 67% when trained on as many as 12 grade levels and tested on 6 grade levels using smoothed unigrams. In Schwarm and Ostendorf [S8] classification accuracy peaked at 79% for distinguishing 5 grade levels whilst adding four syntactical features in addition to lexical language models using a Support Vector Machine (SVM) classifier. In Petersen and Ostendorf [S9], classification accuracy reached 63.18% using lexical-syntactic features on 4 grade levels using a SVM classifier. The dependency of a lexical model on a representative training corpus has either been mitigated by more loosely tying the (language) models to its raw inputs [S7] or by generating genre-specific language models [S10]. Within genres, the maximum correlation with a 5-point readability scale was $r = .817$ [S10]. Either way, this dependency necessitates to specialize lexical-syntactic models for particular genres and populations [S6].

Structuro-cognitivist models typically use linguistically-motivated features, benefiting from the availability of contemporary systems for Natural Language Processing (NLP). These models consider all dimensions of a text: lexical, syntactic, semantic, discourse, and even pragmatic aspects [S6]. Feng et al. [S11] combined a total 273 linguistically-motivated features to achieve a classification accuracy of 74.01% on a Weekly Reader data set with 4 grade levels using Logistic Regression Model (LRM) and SVM classifiers. Ding et al. [S12] achieved 83.72% accuracy over 5 imbalanced classes from the Common Standard Corpus with a Random Forest classifier. The CAREC model [S13] achieved a correlation of $r = .537$ with crowd-sourced readability judgements for 600 excerpts from Wikipedia using stepwise regression from an initial 63 to a final 13 features. Finally, Xia et al. [S14] achieved a classification accuracy of 80.3% and correlation of $r = .900$ on a modified version of the WeeBit corpus using a wide range of linguistically-motivated features with a SVM classifier. These models typically start out with several hundreds of features before feature selection. Their results suggests a strong performance for such all-encompassing, linguistically-motivated models of readability.

Whereas most structuro-cognitivist models are primarily linguistically motivated, several systems appeal to a cognitive motivation. Coh-Metrix is a well-known system, applying cognitively-inspired indexes to indicate the cohesion of a text [S15]. Applied to readability research, Crossley et al. [S16] shows how a regression model based on three criteria of the Coh-Metrix system correlates highly ($r = .925$) with Cloze test results for 32 academic reading texts, in comparison to $r = .691$ for the Dale-Chall formula. Another system is DeLite [S17]. It uses 48 morphological, lexical, syntactic, and semantic cognitively-motivated indicators to increase its content validity.

Compared to the traditional Flesch–Kincaid formula, DeLite's readability predictions correlated more highly with participants' difficulty ratings ($r = .43$ vs. $r = .53$, respectively) for German administrative texts. Another system, TextEvaluator, employs 43 cognitively-motivated features and achieves correlations of up to $r = .81$ for readability of informational texts and of $r = .78$ for literary texts [S18] with a linear regression model. And, Vajjala and Meurers [S2] employed a total of 46 features, derived from second language acquisition research, achieving 93.3% accuracy on five classes from the WeeBit corpus using a multi-layer perceptron. These results suggest a possible performance gain for psychologically valid models that employ fewer, generic features of readability.

A recent development is by non-expert models which employ little feature engineering. Whereas feature engineering requires some level of (linguistic or cognitive) expertise and applies some form of filter on the data, non-expert models use network or graph-based machine learning to infer a data representation from raw inputs. Cha et al. [S19] achieves correlation of $r = .825$ on 5 grade levels for the Common Core Standards corpus using character n-gram word embeddings in combination with K-Means clustering and a Support Vector Regression (SVR) model. Vec2Read [S20] achieves 91.8% binary accuracy on a Wikipedia dataset using character n-gram word embeddings and morphological and syntactic (POS tags) embeddings with a recurrent neural network (Vec2Read). And, Jiang et al. [S21] achieves $F1 = 92.38$ average binary accuracy for 4 grade levels of an English New Concept textbook corpus using tf-idf features with a graph propagation method. These models typically use auxillary tasks and corpora to train their data representations. For example, word embeddings are typically trained using a skipgram task in which a neural network predicts a withheld word. Such word embeddings and neural networks, possibly in combination with linguistically-motivated features [S22, S23, S24, S25], often achieve state-of-the-art performances making them a promising avenue for readability modelling.

The current overview of "AI readability" showed an evolution in modelling approaches, ranging from language models based on rudimentary representations (word n-grams), via extensive feature engineering, to recent word embeddings and neural networks. Nonetheless, strong variations in performance exist within each approach, likely due to variations between data sets and measurements used for evaluation. Even though many studies share the same source of documents (e.g. Weekly Reader, Wikipedias) their selections typically differ. Moreover, concerns about the validity of these corpora have been raised, as they result from the judgements of a small set of experts with often low levels of inter-rater agreement [S6]. This limits the generalisability of resulting models, both to other expert-assessed corpora and to subjective, user-generated ratings. It highlights a tradeoff between domain specificity and generalisability, where the former relies on representative and sufficient training data and the latter on generic principles of readers' processing difficulty.

B Models' linearity

Probablistic Language Models and Generalized Linear Models can be classified as linear models due to their reliance on the additive combination of model terms. In these models, predictions are generated through the linear summation of features weighted by coefficients. Even though these models, through the inclusion of multiplicative terms, can introduce some level of non-linearity, their ability to capture more complex non-linear patterns remains limited. In contrast, neural networks transcend these limitations using their multi-layer architecture and non-linear activation functions. These components allow neural networks to capture a wide range of complex nonlinear patterns in addition to multiplicative interactions. The division between nonlinear and linear methodologies in Table 1 reflects this distinction between the linear and non-linear nature of these models, whilst acknowledging that linear models can capture some degree of

nonlinear relationships.

C EnSiWiki2020 details

The EnSiWiki2020 is based on the Wikipedia dump dated April $1, 2020$, processed to plain text using JWPL [S26], with the removal of templates and links to files and images. The final selection of articles is described in Table S1. Articles were filtered on having a minimal length of 30 words and 5 sentences as determined by a Lucene tokenizer [S27] and Stanford CoreNLP sentence splitter [S28]. Article pairs were established through following language links, which linked Simple English articles to their corresponding English Wikipedia counterparts. This linkage occasionally resulted in multiple Simple English articles being connected to a single English Wikipedia article, contributing to the slight difference in set sizes in Table S1. The oldest $50\,000$ articles were selected that were neither a special, redirect, or disambiguation page. This selection was based on the age rank of the pairs. The pair age rank was determined by the mean age rank of a pair's articles, as determined by ranking both the English and Simple English articles on their first revision date. A non-parametric ranking was preferred over an arithmetic mean of first revision date given the different growth rates of the Wikipedia versions.

		Counts			
Data set	Selection*	Documents	Sentences**	Words**	Characters**
EnWiki	Articles	24 943	176.61	3 943,23	19 762,73
	Sections	138 668	31.17	697,21	3 493,79
	Paragraphs	352 606	7.49	159,92	799,27
SiWiki	Articles	25 057	21.79	335,74	1 582,74
	Sections	39 078	12.86	197,37	930,96
	Paragraphs	40 208	7.04	102,22	479,81
The Guardian		18	11.11	203.61	968,56

Note. *After filtering on texts with a minimum of 5 sentences and 30 words. *After truncation at 1 200 characters. **Average count per document.

Table S1: Descriptive statistics per data set and selection.

D Experimental paradigms of processing difficulty

Building generic models of textual complexity requires capturing fundamental principles of reading. These principles are actively investigated in controlled settings through diverse experimental tasks, each using distinct measures of processing difficulty [S29]. This involves tasks in which word, sentence, and discourse features are compared to readers' ability to process and comprehend the presented information. Analogous to auxiliary tasks for generating embedding models, these tasks can be considered auxiliary to the task of predicting textual complexity. This section will detail the most relevant tasks employed, providing a framework for an external theory of textual complexity.

Typical word-level tasks are the lexical decision and naming tasks [for a review, see S30]. In the lexical decision task participants choose whether a word is an actual word or rather a nonword. The decision speed on this task is affected by a range of word-level characteristics and has contributed to theory development on visual word recognition. In the naming task the rapid

pronunciation of words and pseudowords is tested. Variants on these tasks include (masked) priming, where the relation between a (subconscious) prime and its target are explored. These tasks have been successful in evaluating features of and relations between words on decision speed, such as word length and frequency and semantic relatedness [e.g., S31, S32, S33], and given rise to various models of visual word recognition [S30].

Typical sentence-level tasks involve self-paced reading tasks with sentences that involve some level of ambiguity or complexity, such as the case with garden path sentences [for a review, see S34]. During reading, online measures of reading times and fixation durations are able to reveal subtle changes in language processing using eye-tracking. An alternative task is for participants to complete sentence fragments. When combined with offline measures, such as final comprehension questions or judgments, these tasks explore 'linking hypotheses' between processing and comprehension difficulty. These tasks have successfully shown effects of within-sentence dependencies and word-sequence probabilities on sentence processing difficulty [e.g., S35, S36, S37] and led to related, contrasting theories of sentence processing [S38, S39].

Discourse-level tasks typically involve comprehension tests after self-paced reading of a text [for a review, see S40]. Comprehension is assessed using various comprehension tests, including immediate recall tests, Cloze tests, and tests with multiple choice or open-ended questions. In combination with online measures including reading time and eye-tracking measures, these measures allow the investigation of inferences made during processing. Optimal understanding is thought to be achieved when a coherent mental representation of a text is formed by making inferencing from and integrating individual discourse elements and sentences. The creation of this mental representation is an interplay between the information provided by the text and the background knowledge of a user [S41]. Text comprehension tasks have revealed, among other findings, the interplay between prior knowledge, textual cohesion, and the use of connectives on comprehension [e.g., S42, S43, S44].

The relationship between processing difficulty and comprehension is somewhat equivocal: less-than-optimal word processing may already be sufficient for good discourse comprehension [S45]. This indicates a distinction between processing difficulty and comprehension, suggesting that word-level effects may contribute to processing difficulty without necessarily affecting comprehensibility. As demonstrated by the broad applications of a metric of textual complexity, comprehensibility is just one possible consequence of processing difficulty. Learning outcomes, interest [S46], and relevance judgments [S47] all relate to the ease or difficulty with which a text can be processed. This underscores the need for generalizable models of textual complexity that extend to other corpora and tasks [e.g., interest prediction S46]. To this end, the described experimental paradigms and tasks can be viewed as 'auxiliary tasks' to the target task of predicting textual complexity. Utilizing the findings from these tasks as a foundation for a feature set effectively provides an explicit form of external theory-ladenness, ensuring the relevance of textual features to the phenomenon of textual complexity.

E Hirst-StOnge neighborhood size

With function $r(\varphi, \varphi')$ in Equation 2 we limit the growth of related synsets following the Hirst-StOnge measure of semantic relatedness. This method constrains the paths that can be traversed between nodes to a set of allowable patterns, such as first upward (e.g., hypernymy) followed by horizontal (e.g., similar to) steps [see Fig 13.2a; S48]. The intuition behind these constraints is that both path length and direction changes influence semantic relatedness, the value of which is confirmed through comparisons with human judgements [S49].

F Feature implementation details

For features Dep (dependency length) and $\mathrm{Coh}^{\mathrm{ref}}$ (referential cohesion), the Stanford CoreNLP annotator was used with the tokenize, ssplit, pos, and depparse components for dependency parsing and an additional lemma, ner, and coref component for statistical coreference resolution [S50].

For feature LogPr_n, the CommonCrawl 5-gram language model is used [S51]. Derived from over 9 billion Web pages of the CommonCrawl corpus, making it one of the largest conventional models currently available.

For features $\mathrm{Ent}^{\mathrm{sem}}$ (semantic entropy) and $\mathrm{Coh}^{\mathrm{sem}}$ (semantic cohesion), an Explicit Semantic Analyses (ESA) topic model was created from a Lucene [S27] index of English Wikipedia. Stopwords were removed and all terms were lemmatized using the Snowball stemmer [S52]. This led to a total of 3 734 199 articles or topic dimensions after L2-normalization and pruning [see S53, p. 453]. Each dimension in the ESA model corresponds to a Wikipedia article, rendering the dimensions readily interpretable as topics. This high dimensionality, coupled with the interpretability of its dimensions, sets ESA apart from traditional embedding spaces. Specifically, the calculation of entropy benefits from the granularity afforded by the vast number of dimensions in this high-dimensional topic space. Topical entropy is influenced by both the number of dimensions activated and the heterogeneity of their activation. In a high-dimensional topic space, there's a greater variance in the number of dimensions activated, enabling more precise and fine-grained activation of specific topics. This characteristic offers a particular advantage for calculating topical activation and dispersion for feature $\mathrm{Coh}^{\mathrm{sem}}$ (semantic entropy).

For feature Con (connectives), the AltLex parser [S54] was employed. This parser identifies a wide variety of discourse markers from a parallel sentence Wikipedia corpus by matching phrases with known explicit connectives to parallel phrases that instead contain an alternative lexicalization (AltLex). This method achieves an automatic detection accuracy of 79.58%, navigating the linguistic variation in markers. Explicit markers, though rare, can be identified with high precision, whereas implicit relations, being more prevalent, are harder to automatically recognize. AltLexes fall between these extremes, offering a solution for automatic detection due to their commonality and significant linguistic variety.

G BERT stepwise traversal

A constraint inherent in embedding models, such as BERT, pertains to the scope of context they can encompass. This scope is confined to a span of 512 words, less than the typical length of our training texts (Wikipedia articles). Truncating articles at 512 tokens would amount to a substantial loss of information, which has been related to performance losses [S5]. This limitation is resolved by a sliding window approach involving overlapping windows. As depicted in Figure S2, this approach involves the stepwise traversal of an area of interest consisting of 256 tokens. Preceding and subsequent tokens are added to form a contiguous input window of size 512. This approach guarantees that each token in a text is included in an area of interest once. The inclusion of appropriate leading and trailing context enables BERT to generate accurate and contextually informed word embeddings.

H Feature space inflation

The inclusion of interaction terms results in the expansion of the feature space. The number of feature combinations reaches 325 second-order and 2600 third-order combinations for Feature

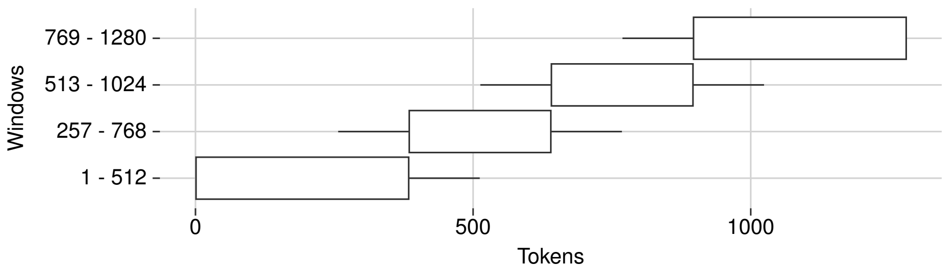

Figure S2: Sliding window approach applied to accommodate for BERT's input size restriction, illustrated for a text of 1280 tokens. Boxes indicate the area of interest used for further processing. Whiskers indicate leading and trailing tokens ensuring properly contextualized word embeddings.

Set I, and 294 528 and $7.52\mathrm{e}{-7}$ combinations for Feature Set II. The latter upper limit surpasses the total number of observations (50 000), raising concerns about the potential for spurious relationships. Additionally, the computational complexity $\mathcal{O}(np^2+p^3)$ of regression modeling, where n represents the number of observations and p signifies the number of features, escalates rapidly beyond reasonable computational capacity.

I Hyperparameters and control mechanisms

For FNN (Feedforward Neural Network) training, the following hyperparameters and control mechanisms were used:

Adam optimizer The Adam optimizer was used as an adaptive learning algorithm that self-tunes during training. It combines the first moment (mean) and second moment (uncentered variance) of gradients to adaptively adjust the learning rate during training, promoting swift convergence and robustness [S55]. These characteristics make it a standard choice for optimization tasks.

Early stopping Test performance was validated every 10 training iterations (epochs) starting from epoch 20. If, in a window of 50 epochs, test performance did not increase, the best performing model from that window was selected and training was stopped. These stopping criteria were chosen after initial inspection of validation error curves, suggesting a patience of 50 epochs was needed for consistent and robust results. Early stopping serves to prevent overfitting by terminating training when a divergence between improvements in training and validation errors emerges [S56].

Learning rate The initial learning rate set for the Adam optimizer was considered a hyperparameter. The values of *lr_rate* $= 1\mathrm{e}{-5} \ldots 1\mathrm{e}{-1}$ were evaluated. A range of learning rates was employed to empirically strike a balance between training speed and smooth and consistent convergence of the training process.

Weight decay The weight decay hyperparameter controls the magnitude of the L2 regularization term within the Adam optimizer. L2 regularlization constrains model parameters ensuring that all network nodes contribute to some extent. Contrary to the L1 (Lasso) regularlization term customary with GLM, this regularlization does not eliminate terms from the model. The values of *weight_decay* $= 1\mathrm{e}{-4} \ldots 1\mathrm{e}{-1}$ were evaluated.

Dropout rate The dropout rate dictates the proportion of neurons that are temporarily deactivated during training iterations. This technique introduces a degree of randomness, preventing specific neurons from becoming overly dependent on others and promoting more robust and generalized network representations. To enhance the capacity to generalize to unseen data, dropout values of *dropout* $= 0, 0.1, 0.2, 0.3, 0.4$ were tuned.

Activation function A neural network derives its nonlinear capacity from its activation function. Various non-linear activation functions, including the hyperbolic tangent (tanh), rectified linear unit (ReLU), softmax, and sigmoid were explored for this hyperparameter. Each function imparts distinct assumptions regarding the relations between neurons in the model, enabling it to capture intricate patterns within the data.

J Probablistic language models' hyperparameters

For probablistic language models, n-grams provide an intuitive way to control the 'depth' of the model, whilst the number of included words offers further control of the number of parameters. A total of 25 distinct PLMs were evaluated, spanning n-gram ranges from 1 to 5, and vocabulary extents of 250, 500, 1000, 10000, and 100000. The vocabulary selection process followed an information-gain ranking methodology as outlined in [S9]. This ranking strategy assigns higher priority to terms that exhibit a high frequency within a specific class while maintaining relative rarity in other classes. This approach is designed to capture terms that offer significant discriminatory power across readability classes. Terms that exceeded the prescribed vocabulary limits were replaced with their corresponding POS tags. This supplementary procedure incorporates syntactic features into the Probablistic Language Model (PLM) against only a marginal expansion of the model's parameter count. Early developments on Language Model (LM)-based readability classification showed that this integration of syntactic features has a positive impact on classification performance and enhances the model's ability to generalize to previously unseen texts [S8, S9].

K ChatGPT prompt

The prompt given to ChatGPT was:

> "*Below is an instruction that describes a task, paired with an input that provides further context. Write a response that appropriately completes the request. Instruction: Detect the reading level of the text. Write the reading level on a scale of "very difficult", "difficult", "fairly difficult", "standard", "fairly easy", "easy", "very easy". Also, give the reading level on a continuous scale from 0-1, where 0 denotes very easy and 1 very difficult.*
> *Input: "[text]"*
> *Reading level (label): ?*
> *Reading level (0-1): ?*"

The placeholder "[text]" was substituted by a target text.

References

S1. Vajjala, S., and Meurers, D. (2014). Assessing the relative reading level of sentence pairs for text simplification. In: Proceedings of the 14th Conference of the European Chapter of the Association for Computational Linguistics. Association for Computational Linguistics (288–297). doi:10.3115/v1/E14-1031.

S2. Vajjala, S., and Meurers, D. (2012). On improving the accuracy of readability classification using insights from second language acquisition. In: Proceedings of the Seventh Workshop on Building Educational Applications Using NLP. NAACL HLT '12 Association for Computational Linguistics (163–173).

S3. Crossley, S., Heintz, A., Choi, J. S., Batchelor, J., Karimi, M., and Malatinszky, A. (2023). A large-scaled corpus for assessing text readability. Behav. Res. Methods *55*, 491–507. doi:10.3758/s13428-022-01802-x.

S4. Madrazo Azpiazu, I., and Pera, M. S. (2020). Is cross-lingual readability assessment possible? J. Assoc. Inf. Sci. Technol. *71*, 644–656. doi:10.1002/asi.24293.

S5. Martinc, M., Pollak, S., and Robnik-Šikonja, M. (2021). Supervised and unsupervised neural approaches to text readability. Comput. Linguist. *47*, 141–179. doi:10.1162/coli_a_00398.

S6. François, T. (2015). When readability meets computational linguistics: A new paradigm in readability. Revue Française de Linguistique Appliquée *20*, 79–97. doi:10.3917/rfla.202.0079.

S7. Collins-Thompson, K., and Callan, J. (2005). Predicting reading difficulty with statistical language models. J. Assoc. Inf. Sci. Technol. *56*, 1448–1462. doi:10.1002/asi.20243.

S8. Schwarm, S. E., and Ostendorf, M. (2005). Reading level assessment using support vector machines and statistical language models. In: Proceedings of the 43rd Annual Meeting on Association for Computational Linguistics. ACL '05 Association for Computational Linguistics (523–530).

S9. Petersen, S. E., and Ostendorf, M. (2009). A machine learning approach to reading level assessment. Computer Speech & Language *23*, 89–106. doi:10.1016/j.csl.2008.04.003.

S10. Kate, R. J., Luo, X., Patwardhan, S., Franz, M., Florian, R., Mooney, R. J., Roukos, S., and Welty, C. (2010). Learning to predict readability using diverse linguistic features. In: Proceedings of the 23rd International Conference on Computational Linguistics. COLING '10 Association for Computational Linguistics (546–554).

S11. Feng, L., Jansche, M., Huenerfauth, M., and Elhadad, N. (2010). A comparison of features for automatic readability assessment. In: Proceedings of the 23rd International Conference on Computational Linguistics: Posters. Association for Computational Linguistics (276–284).

S12. Ding, H., Zhong, Q., Zhang, S., and Yang, L. (2022). Text difficulty classification by combining machine learning and language features. In: Xie, Q., Zhao, L., Li, K., Yadav, A., and Wang, L., eds. Advances in Natural Computation, Fuzzy Systems and Knowledge Discovery: Proceedings of the ICNC-FSKD 2021 vol. 89 of *Lecture Notes on Data Engineering and Communications Technologies*. Springer International Publishing (1055–1063). doi:10.1007/978-3-030-89698-0_108.

S13. Crossley, S. A., Skalicky, S., and Dascalu, M. (2019). Moving beyond classic readability formulas: new methods and new models. J. Res. Read. *42*, 541–561. doi:10.1111/1467-9817.12283.

S14. Xia, M., Kochmar, E., and Briscoe, T. (2016). Text readability assessment for second language learners. In: Proceedings of the 11th Workshop on Innovative Use of NLP for Building Educational Applications. Association for Computational Linguistics (12–22). doi:10.18653/v1/W16-0502.

S15. Graesser, A., McNamara, D., Louwerse, M., and Cai, Z. (2004). Coh-Metrix: Analysis of text on cohesion and language. Behav. Res. Methods *36*, 193–202.

S16. Crossley, S., Greenfield, J., and McNamara, D. (2008). Assessing text readability using cognitively based indices. TESOL Quarterly *42*, 475–493.

S17. Vor der Brück, T., Hartrumpf, S., and Helbig, H. (2008). A readability checker with supervised learning using deep indicators. Informatica *32*, 429–435.

S18. Sheehan, K. M., Kostin, I., Napolitano, D., and Flor, M. (2014). The TextEvaluator tool. Elementary School Journal *115*, 184–209. doi:10.1086/678294.

S19. Cha, M., Gwon, Y., and Kung, H. T. (2017). Language modeling by clustering with word embeddings for text readability assessment. In: Proceedings of the 2017 ACM on Conference on Information and Knowledge Management - CIKM '17. ACM Press (2003–2006). doi:10.1145/3132847.3133104.

S20. Azpiazu, I. M., and Pera, M. S. (2019). Multiattentive recurrent neural network architecture for multilingual readability assessment. Trans. Assoc. Comput. Linguist. *7*, 421–436. doi:10.1162/tacl_a_00278.

S21. Jiang, Z., Gu, Q., Yin, Y., Wang, J., and Chen, D. (2019). GRAW+: A two-view graph propagation method with word coupling for readability assessment. J. Assoc. Inf. Sci. Technol. *70*, 433–447. doi:10.1002/asi.24123.

S22. Lee, B. W., Jang, Y. S., and Lee, J. (2021). Pushing on text readability assessment: A transformer meets handcrafted linguistic features. In: Moens, M.-F., Huang, X., Specia, L., and Yih, S. W.-t., eds. Proceedings of the 2021 Conference on Empirical Methods in Natural Language Processing. Association for Computational Linguistics (10669–10686). doi:10.18653/v1/2021.emnlp-main.834.

S23. Imperial, J. M. (2021). BERT embeddings for automatic readability assessment. In: Mitkov, R., and Angelova, G., eds. Proceedings of the International Conference on Recent Advances in Natural Language Processing (RANLP 2021). INCOMA Ltd. (611–618).

S24. Lee, J., and Vajjala, S. (2022). A neural pairwise ranking model for readability assessment. In: Muresan, S., Nakov, P., and Villavicencio, A., eds. Findings of the Association for Computational Linguistics: ACL 2022. Association for Computational Linguistics (3802–3813). doi:10.18653/v1/2022.findings-acl.300.

S25. Liu, F., and Lee, J. (2023). Hybrid models for sentence readability assessment. In: Kochmar, E., Burstein, J., Horbach, A., Laarmann-Quante, R., Madnani, N., Tack, A., Yaneva, V., Yuan, Z., and Zesch, T., eds. Proceedings of the 18th Workshop on Innovative Use of NLP for Building Educational Applications (BEA 2023). Association for Computational Linguistics (448–454). doi:10.18653/v1/2023.bea-1.37.

S26. Zesch, T., Müller, C., and Gurevych, I. (2008). Extracting lexical semantic knowledge from Wikipedia and Wiktionary. In: Proceedings of the Sixth International Conference on Language Resources and Evaluation (LREC'08). European Language Resources Association (ELRA).

S27. Hatcher, E., Gospodnetic, O., and McCandless, M. Lucene in Action. Second revised ed. Manning Publications Co. (2010).

S28. Manning, C., Surdeanu, M., Bauer, J., Finkel, J., Bethard, S., and McClosky, D. (2014). The Stanford CoreNLP natural language processing toolkit. In: Bontcheva, K., and Zhu, J., eds. Proceedings of 52nd Annual Meeting of the Association for Computational Linguistics: System Demonstrations. Baltimore, Maryland: Association for Computational Linguistics (55–60). doi:10.3115/v1/P14-5010.

S29. Rueschemeyer, S.-A., and Gaskell, M. G., eds. The Oxford handbook of psycholinguistics. Oxford University Press (2018). doi:10.1093/oxfordhb/9780198786825.001.0001.

S30. Rastle, K. (2018). Visual word recognition. In: Rueschemeyer, S.-A., and Gaskell, M. G., eds. The Oxford handbook of psycholinguistics. Oxford University Press (47–70). doi:10.1093/oxfordhb/9780198786825.013.3.

S31. McGinnies, E., Comer, P., and Lacey, O. (1952). Visual-recognition thresholds as a function of word length and word frequency. J. Exp. Psychol. *44*, 65–69. doi:10.1037/h0063142.

S32. Balota, D. A., Cortese, M. J., Sergent-Marshall, S. D., Spieler, D. H., and Yap, M. J. (2004). Visual word recognition of single-syllable words. J. Exp. Psychol. Gen. *133*, 283–316. doi:10.1037/0096-3445.133.2.283.

S33. New, B., Ferrand, L., Pallier, C., and Brysbaert, M. (2006). Reexamining the word length effect in visual word recognition: New evidence from the English lexicon project. Psychon. B. Rev. *13*, 45–52. URL: 10.3758/BF03193811. doi:10.3758/BF03193811.

S34. MacDonald, M. C., and Hsiao, Y. (2018). Sentence comprehension. In: Rueschemeyer, S.-A., and Gaskell, M. G., eds. The Oxford handbook of psycholinguistics. Oxford University Press (170–196). doi:10.1093/oxfordhb/9780198786825.013.8.

S35. Boston, M. F., Hale, J., Kliegl, R., Patil, U., and Vasishth, S. (2008). Parsing costs as predictors of reading difficulty: An evaluation using the Potsdam sentence corpus. Journal of Eye Movement Research *2*. doi:10.16910/jemr.2.1.1.

S36. Liu, H., Xu, C., and Liang, J. (2017). Dependency distance: A new perspective on syntactic patterns in natural languages. Phys. Life Rev. *21*, 171–193. doi:10.1016/j.plrev.2017.03.002.

S37. Rajkumar, R., van Schijndel, M., White, M., and Schuler, W. (2016). Investigating locality effects and surprisal in written English syntactic choice phenomena. Cognition *155*, 204–232. doi:10.1016/j.cognition.2016.06.008.

S38. Smith, N. J., and Levy, R. (2013). The effect of word predictability on reading time is logarithmic. Cognition *128*, 302–319. doi:10.1016/j.cognition.2013.02.013.

S39. Gibson, E., Futrell, R., Piantadosi, S. P., Dautriche, I., Mahowald, K., Bergen, L., and Levy, R. (2019). How efficiency shapes human language. Trends Cogn. Sci. *23*, 389–407. doi:10.1016/j.tics.2019.02.003.

S40. Ferstl, E. C. (2018). Text comprehension. In: Rueschemeyer, S.-A., and Gaskell, M. G., eds. The Oxford handbook of psycholinguistics. Oxford University Press (196–216). doi:10.1093/oxfordhb/9780198786825.013.9.

S41. Kintsch, W., and van Dijk, T. A. (1978). Toward a model of text comprehension and production. Psychol. Rev. *85*, 363 – 394. doi:10.1037/0033-295X.85.5.363.

S42. Haberlandt, K. (1982). Reader expectations in text comprehension. In: Language and Comprehension vol. 9 of *Advances in Psychology*. Elsevier (239–249). doi:10.1016/S0166-4115(09)60055-8.

S43. Graesser, A. C., Singer, M., and Trabasso, T. (1994). Constructing inferences during narrative text comprehension. Psychol. Rev. *101*, 371–395. doi:10.1037/0033-295x.101.3.371.

S44. Kleijn, S., Pander Maat, H. L., and Sanders, T. J. (2019). Comprehension effects of connectives across texts, readers, and coherence relations. Discourse Process. *56*, 447–464. doi:10.1080/{0163853X}.2019.1605257.

S45. Long, D. L., Wilson, J., Hurley, R., and Prat, C. S. (2006). Assessing text representations with recognition: The interaction of domain knowledge and text coherence. J. Exp. Psychol. Learn. Mem. Cogn. *32*, 816–827.

S46. van der Sluis, F., and van den Broek, E. L. (2023). Feedback beyond accuracy: Using eye-tracking to detect comprehensibility and interest during reading. J. Assoc. Inf. Sci. Technol. *74*, 3–16. doi:10.1002/asi.24657.

S47. Collins-Thompson, K., Bennett, P. N., White, R. W., de la Chica, S., and Sontag, D. (2011). Personalizing web search results by reading level. In: Berendt, B., de Vries, A., Fan, W., Macdonald, C., Ounis, I., and Ruthven, I., eds. Proceedings of the 20th ACM International Conference on Information and Knowledge Management. CIKM '11 ACM (403–412). doi:http://doi.acm.org/10.1145/2063576.2063639.

S48. Miller, G. A. (1995). WordNet: A lexical database for English. Commun. ACM *38*, 39–41. doi:http://doi.acm.org/10.1145/219717.219748.

S49. Budanitsky, A., and Hirst, G. (2006). Evaluating WordNet-based measures of lexical semantic relatedness. Comput. Linguist. *32*, 13–47. doi:10.1162/coli.2006.32.1.13.

S50. Clark, K., and Manning, C. D. (2015). Entity-centric coreference resolution with model stacking. In: Zong, C., and Strube, M., eds. Proceedings of the 53rd Annual Meeting of the Association for Computational Linguistics and the 7th International Joint Conference on Natural Language Processing (Volume 1: Long Papers). Association for Computational Linguistics (1405–1415). doi:10.3115/v1/P15-1136.

S51. Buck, C., Heafield, K., and van Ooyen, B. (2014). N-gram counts and language models from the Common Crawl. In: Calzolari, N., Choukri, K., Declerck, T., Loftsson, H., Maegaard, B., Mariani, J., Moreno, A., Odijk, J., and Piperidis, S., eds. Proceedings of the Ninth International Conference on Language Resources and Evaluation (LREC'14). European Language Resources Association (ELRA) (3579–3584).

S52. Porter, M. F. (2001). Snowball: A language for stemming algorithms. Available online at http://snowball.tartarus.org/texts/introduction.html [Last accessed on December 17, 2024].

S53. Gabrilovich, E., and Markovitch, S. (2009). Wikipedia-based semantic interpretation for natural language processing. J. Artif. Intell. Res. *34*, 443–498.

S54. Hidey, C., and McKeown, K. (2016). Identifying causal relations using parallel Wikipedia articles. In: Proceedings of the 54th Annual Meeting of the Association for Computational Linguistics (Volume 1: Long Papers). Association for Computational Linguistics (1424–1433). doi:10.18653/v1/P16-1135.

S55. Kingma, D. P., and Ba, J. (2014). Adam: A method for stochastic optimization. Preprint at arXiv. doi:10.48550/arxiv.1412.6980.

S56. Prechelt, L. (2012). Early stopping —but when? In: Montavon, G., Orr, G. B., and Müller, K.-R., eds. Neural networks: tricks of the trade vol. 7700 of *Lecture notes in computer science*. Springer Berlin Heidelberg (53–67). doi:10.1007/978-3-642-35289-8_5.
